# Comprehensive Chemical Characterisation of Byzantine Glass Weights

**DOI:** 10.1371/journal.pone.0168289

**Published:** 2016-12-13

**Authors:** Nadine Schibille, Andrew Meek, Bendeguz Tobias, Chris Entwistle, Mathilde Avisseau-Broustet, Henrique Da Mota, Bernard Gratuze

**Affiliations:** 1 IRAMAT-CEB, UMR 5060, CNRS, Orléans, France; 2 Department of Scientific Research, The British Museum, London, United Kingdom; 3 Universität Innsbruck, Institut für Archäologien, Fachbereich Ur- und Frühgeschichte sowie Mittelalter und Neuzeitarchäologie, Innsbruck, Austria; 4 Department of Britain, Europe and Prehistory, The British Museum, London, United Kingdom; 5 Départment des monnaies, médailles et antiques, Bibliothèque nationale de France, Paris, France; University of Oxford, UNITED KINGDOM

## Abstract

The understanding of the glass trade in the first millennium CE relies on the characterisation of well-dated compositional groups and the identification of their primary production sites. 275 Byzantine glass weights from the British Museum and the Bibliothèque nationale de France dating to the sixth and seventh century were analysed by LA-ICP-MS. Multivariate statistical and graphical data analysis discriminated between six main primary glass types. Primary glass sources were differentiated based on multi-dimensional comparison of silica-derived elements (MgO, Al_2_O_3_, CaO, TiO_2_, Fe_2_O_3_, ZrO_2_) and components associated with the alkali source (Li_2_O, B_2_O_3_). Along with Egyptian and Levantine origins of the glassmaking sands, variations in the natron source possibly point to the exploitation of two different natron deposits. Differences in strontium to calcium ratios revealed variations in the carbonate fractions in the sand. At least two cobalt sources were employed as colouring agents, one of which shows strong correlations with nickel, indicating a specific post-Roman cobalt source. Typological evidence identified chronological developments in the use of the different glass groups. Throughout the sixth century, Byzantine glass weights were predominately produced from two glasses that are probably of an Egyptian origin (Foy-2 and Foy-2 high Fe). Towards the second half of the sixth century a new but related plant-ash glass type emerged (Magby). Levantine I was likewise found among the late sixth- to early seventh-century samples. The use of different dies for the same batch testifies to large-scale, centralised production of the weights, while the same die used for different primary production groups demonstrates the co-existence of alternative sources of supply. Given the comprehensive design of our study, these results can be extrapolated to the wider early Byzantine glass industry and its changes at large.

## Introduction

The history of Byzantine glass-making and working as a distinct category remains largely unwritten, despite recent scholarly attention [1–8]. Analytical and archaeological evidence testifies to on-going primary production in the Levant and Egypt from the Roman period and throughout the Middle Ages, and continuing after the Arab conquest [9–13]. Different primary glass production groups have been identified in the Mediterranean, and beyond, dating to the late antique and early medieval periods [7, 14, 15]. They differ in their compositional characteristics, indicating the use of different raw materials, particularly silica sources, and by extension different primary production locations. Two Levantine groups (Levantine I & II) tend to have relatively high alumina and lime concentrations, while they are lower in heavy minerals compared to Egyptian glasses (Egypt I & II) (for a comprehensive discussion of the chemical characteristics of these groups see [16]). Even higher quantities of iron, titanium and zirconium oxides characterise the so-called Foy-2 and HIMT (High Iron, Manganese and Titanium) glasses that are believed to have likewise been produced from an Egyptian silica source [15, 17–20]. Large primary glassmaking furnaces have been discovered in Egypt [13] and on the Levantine coast [11, 21], but only the Levantine groups could be unequivocally related to these sites due to the archaeological remains. All other groups were defined by their compositional characteristics in relation to what we know about distribution patterns and Egyptian and Levantine sand sources [12, 17]. The two Egyptian groups, for instance, were originally recognised by Gratuze and Barrandon on the basis of the analytical study of seventy Islamic glass weights from successive dynasties from the Umayyad through to the Mamluk period [22, 23]. The majority of the Islamic glass weights can be precisely dated to within a few years, either due to the occasional date or, more frequently, the name of the governor who was responsible for their commission, being embossed on the glass weight. The discrete chronological attribution has enabled the authors to trace the developments of medieval and early Islamic glass compositions in great detail [23].

**Fig 1:**
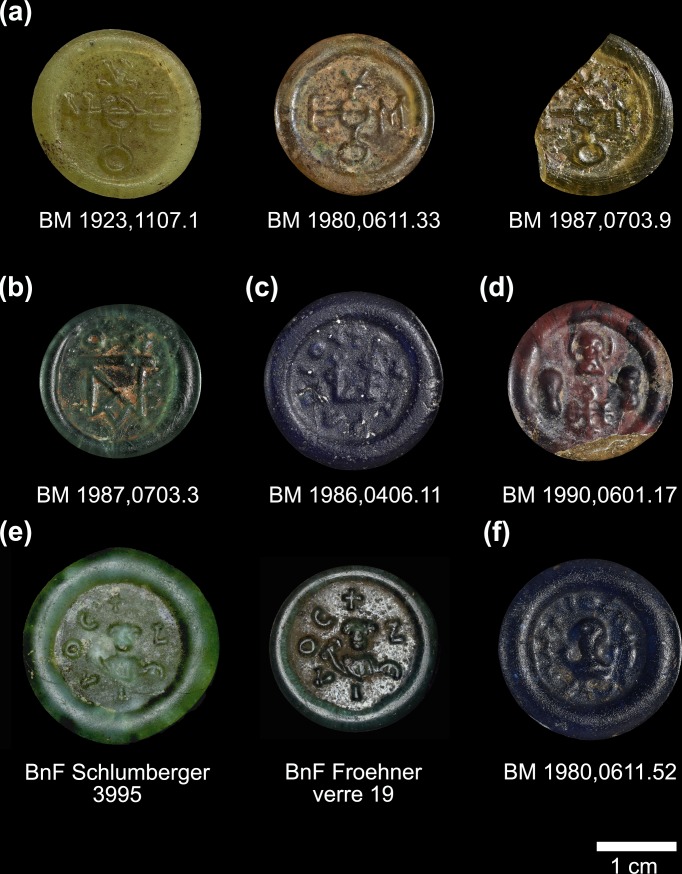
Selection of Byzantine Glass weights representing the different typologies. (a) Weights with cruciform monogram, belonging to batch 11. (b) Weight with box monogram. (c) Weight with box monogram enclosed by an inscription. (d) Imperial weight with imperial busts and box monogram; (e) Bust of an eparch enclosed by an inscription. Two weights produced with identical die, but different base glasses. (f) So-called Arab-Byzantine weight with pseudo-Cufic inscription. Objects and images under a CC BY-NC-SA 4.0 license with permission from the British Museum, original copyright *The Trustees of the British Museum* (*a-d*, *f*); and from the Bibliothèque nationale de France (*e*), original copyright IRAMAT-CEB Orléans.

Despite the important information that the typological and analytical studies of glass weights can offer about the metrological and fiscal systems and the technological evolution in relation to geopolitical changes, Byzantine glass weights have hardly been investigated (for an exception see [24]). The present paper presents new analytical data for 275 Byzantine glass weights in order to explore the characteristics and distribution of primary glass in relation to secondary workshop practices during the early Byzantine period. The glass weights under investigation are from the medieval collection in the British Museum (n = 179 samples; henceforth BM) and the Cabinet des monnaies, médailles et antiques in the Bibliothèque nationale de France (n = 96; henceforth BnF) in Paris. Neither collection of weights is particularly well provenanced and the history of the individual pieces prior to their purchase from public and private collections is largely unknown (e.g. [24–26]). With few exceptions, the Byzantine glass weights are discoidal in shape with bulging rim and a diameter ranging from 12 mm to 30 mm, a thickness of about 3 mm to 6 mm, and a weight between approximately 0.4 g and 4.5 g. They were produced in a variety of colours, with dark cobalt blue, olive green, aqua bluish and pale green prevailing (S1 Table). There is a small number (n = 8) of large, heavy weights, the heaviest weighing 316 g. Different iconographic types are represented among the two assemblages and include box and cruciform monograms, monograms enclosed by an inscription, one or more imperial busts sometimes accompanied with a monogram, an imperial bust or a bust of an eparch surrounded by an inscription, and finally what was described as ‘Arab-Byzantine’ with debased monograms or busts with no identifying inscription (Fig 1). These ‘Arab-Byzantine’ weights are generally dated to the time immediately following the Fall of Egypt to the Arabs in the 640s and before purely Arabic glass weights were issued by Abd al-Malik in 691 CE [24, 27, 28].

The original function of the small glass discs was most certainly to verify and control the weight of gold coinage, while the large, heavy weights might have been used as commodity weights [29]. Although no denominational marks are usually given on the Byzantine glass weights, an exact calibration is often inscribed on their early Islamic counterparts. The masses of early Islamic glass weights correspond accordingly to those of the dinar, the dirham or their divisions ([27, 30] p. 33, 37). What is more, a statistical study of the mass of 650 Byzantine glass weights conducted at the British Museum in 1994 demonstrated a distribution centred on the theoretical weight units of the gold solidus nomisma (4.50 g) and its divisions, the semissis (2.25 g) and tremissis (1.50 g) [31–33]. Hence, there is good reason to believe that Byzantine glass weights were used as coin weights. As such, their production and administration might have been under the direct jurisdiction of the *Comes Sacrarum Largitionum*, who was, according to the Codex Justinianus (Nov. 128, cap. 15), among the most important financial officers in the Byzantine Empire responsible for the weight of gold, silver and other metals [34]. It is therefore likely that the manufacture of glass weights was closely linked to the early Byzantine imperial mints at Constantinople, Alexandria, Carthage and/or Ravenna [35].

In terms of chronological attribution, Byzantine glass weights can be dated to the sixth and seventh century CE. Well-dated archaeological finds unambiguously show that glass weights were in use during the reign of Justinian I (527–565 CE) (e.g. Hajdučka Vodenica and Bet Shean: [36] p. 179 with footnote 1; [37] p. 79 Nr. 236; [38] pp. 492–498; [39] pp. 451f). The latest weights were found in contexts dated to the reign of Constans II (641–668 CE) (e.g. Katalimata: [40] pp. 85–87). The dating of individual weights can sometimes be gleaned from the names given in the form of inscriptions or monograms (S1 Table). The names on Byzantine glass weights are those of officials of the Byzantine financial system such as the Pretorian Prefect (especially for Oriens), the above mentioned *Comes Sacrarum Largitionum*, the office of the *res private and patrimonia*, the city prefect or the *Quaestor Iustianus Exercitus* [41]. The Byzantine financial system changed at the turn from the sixth to the seventh century CE, when the city prefect obtained a more central role. During the course of the first half of the seventh century, the offices of the Pretorian Prefect and the *Comes Sacrarum Largitionum* eventually disappeared or were transferred to other financial offices.

Through the analysis of major, minor and trace elements by LA-ICP-MS, this paper seeks to shed light on the processes and organisation of the production of Byzantine glass weights, including the supply of raw materials and their colorants, and the extent to which recycled material was used. In conjunction with the typological data, our results reveal distinctive compositional patterns and identify several glass batches that are crucial to the understanding of the secondary working traditions and the scale of production. The majority of the Byzantine glass weights were made from two different types of natron glass. As will be discussed, one of these natron-type glasses was probably produced in Egypt, the other on the Levantine coast. This paper makes a substantial contribution to unravelling the ‘medieval mystery’ of Byzantine glass by identifying fundamental geographical and chronological parameters in relation to the base glass composition, additives and secondary working practices of these specifically Byzantine artefacts [4, 42].

## Methods

179 Byzantine glass weights housed in the medieval collection of the British Museum and 96 specimens now in the Cabinet des monnaies, médailles et antiques in the Bibliothèque nationale de France in Paris were analysed without prior sample preparation. Analysis by Laser Ablation Inductively Coupled Plasma Mass Spectrometry (LA-ICP-MS) was carried out at the Centre Ernest-Babelon of the IRAMAT (Orléans), using an Element XR mass spectrometer (Thermofisher) and a RESOlution M50e ArF excimer laser probe ablation device (Resonetics) as described in [43]. The operating conditions of the 193 nm laser were set at an energy of 4 to 6 mJ, with a repetition rate of 10 Hz and a spot size diameter of 100 μm. Where necessary, laser repetition rate and spot size were reduced to 6 Hz and 60 μm to avoid manganese saturation. The pre-ablation time of 20 seconds was occasionally increased, depending on the thickness of the surface corrosion layer. The analytical time was set at 50 seconds and the measurements were carried out in peak jump acquisition mode on a list of pre-selected isotopes, defined so as to avoid isobaric interferences. Fifty-eight elements were measured by spot analysis, including major and minor glass constituents, colouring and opacifying agents as well as other trace and rare earth elements. For silicon, the ^28^Si isotope was determined and used as internal standard.

An average response factor K_Y_ that allows the signals to be converted into fully quantitative data was calculated using a combination of five different standard reference materials (SRM) that cover the range of archaeological glass compositions: NIST SRM610 (soda-lime-silica glass), Corning Glass Standards B (plant ash glass), C (lead-barium-potash glass) and D (lime-potash glass) and an in-house archaeological glass standard analysed by fast neutron activation analysis for the calculation of chlorine concentrations. Corning A and NIST SRM612 were analysed at regular intervals throughout the analytical sequence to establish the accuracy and precision of the data. The correspondence between certified values and measured values for the two reference materials is generally good with an accuracy better than 5% for most major and minor elements and within 5–10% for minor and trace elements. The relative standard deviation calculated from repeated measurements is typically ≤ 5% (Table 1). Detection limits vary according to the ablation parameters and the optimisation parameter of the mass spectrometer. Typical detection limits for soda-lime and potassium-lime glasses are listed elsewhere ([44] Table 13.4). In our measurements, Cr, Se, Sb, Pt and Au occasionally fall below the detection limits of 1 ppm (< 10 ppm for Cr).

**Table 1:** LA-ICP-MS data of glass standards in comparison with published values. Corning A corresponds to given data from [45], Nist 612 corresponds to data in [46], ^a^ after [47] and ^b^ after [48].

	**Li**_**2**_**O**	**B**_**2**_**O**_**3**_	**Na**_**2**_**O**	**MgO**	**Al**_**2**_**O**_**3**_	**SiO**_**2**_	**P**_**2**_**O**_**5**_	**Cl**	**K**_**2**_**O**	**CaO**	**TiO**_**2**_	**V**_**2**_**O**_**5**_	**MnO**	**Fe**_**2**_**O**_**3**_	**CoO**	**NiO**	**CuO**	**Rb**_**2**_**O**	**SrO**	**ZrO**_**2**_	**SnO**_**2**_	**Sb**_**2**_**O**_**3**_	**BaO**	**PbO**	**Bi**														
Corning A (measured)	0.01	0.21	13.89	2.53	0.93	66.69	0.12	0.14	2.83	5.81	0.78	0.01	1.02	1.10	0.17	0.02	1.18	0.01	0.10	0.01	0.17	1.64	0.46	0.06	0.00														
relative σ (n = 30)	2.70	3.55	1.58	2.82	3.53	0.56	10.25	4.52	1.10	3.12	2.35	2.93	2.01	1.96	2.81	2.54	1.88	2.28	1.72	3.98	2.14	3.14	2.79	3.30	2.38														
Vicenzi	0.01	0.20	14.30	2.66	1.00	66.56	0.13	0.10	2.87	5.03	0.79	0.01	1.00	1.09	0.17	0.02	1.17	0.01	0.10	0.01	0.19	1.75	0.56	0.073^a^	0.00														
accuracy	7.78	3.99	-2.88	-4.70	-6.59	0.19	-9.81	41.98	-1.34	15.45	-1.46	4.38	2.36	1.23	-0.83	13.45	0.93	-6.70	3.21	5.44	-8.79	-6.37	-17.58	-17.83	-14.93														
	**V**	**Cr**	**Mn**	**Co**	**Ni**	**Cu**	**Zn**	**Ga**	**As**	**Rb**	**Sr**	**Y**	**Zr**	**Nb**	**Mo**	**In**	**Sn**	**Sb**	**Cs**	**Ba**	**La**	**Ce**	**Pr**	**Nd**	**Sm**	**Eu**	**Gd**	**Tb**	**Dy**	**Ho**	**Er**	**Tm**	**Yb**	**Lu**	**Hf**	**Ta**	**Pb**	**Bi**	**U**
NIST 612 (measured)	38.25	32.55	40.24	34.83	37.89	35.85	38.11	36.96	35.19	32.18	75.86	37.21	37.62	34.73	33.72	37.58	34.94	37.19	41.71	36.97	36.80	37.53	37.07	35.95	37.62	36.08	35.21	36.80	34.29	37.00	35.14	34.53	38.24	35.16	35.49	30.73	32.10	31.13	36.62
relative σ (n = 31)	1.59	25.11	1.61	2.09	2.31	2.65	3.93	1.63	3.65	1.52	2.05	2.89	2.70	3.57	3.74	1.58	1.52	7.37	1.93	1.94	6.51	6.44	4.28	2.38	1.82	2.67	7.41	2.77	1.61	2.97	1.81	3.05	1.35	3.42	2.21	2.95	10.81	3.74	4.53
Jochum	39.90	36.26	39.40	34.82	37.90	38.70	41.20	34^b^	36.80	31.07	78.51	40.14	40.36	41.54	35.79	41^b^	40.90	36.40	42.14	39.37	34.65	37.25	37.31	34.96	37.15	34^b^	38.56	39.96	36.15	38.69	38.86	36*	39.84	35*	37.13	38.17	38.60	40*	37.68
accuracy	-4.13	-10.23	2.13	0.03	-0.03	-7.35	-7.49	8.69	-4.36	3.59	-3.37	-7.30	-6.80	-16.38	-5.77	-8.34	-14.58	2.17	-1.02	-6.09	6.20	0.76	-0.63	2.84	1.28	6.12	-8.69	-7.91	-5.15	-4.37	-9.57	-4.07	-4.01	0.46	-4.43	-19.49	-16.84	-22.19	-2.81

**Fig 2:**
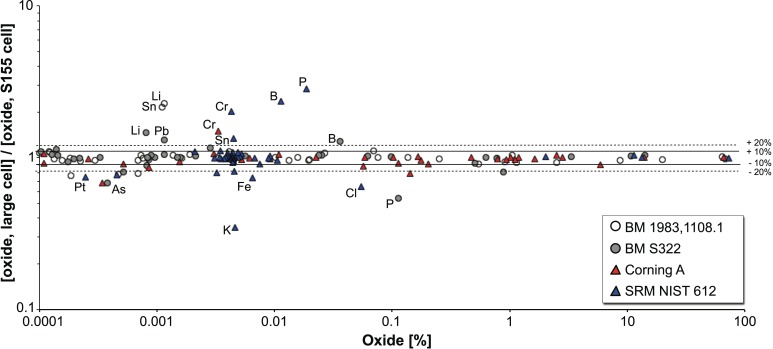
Comparison of the results obtained with the big cell and the standard S155 cell. The graph shows the correspondence of the experimental results for Corning A, NIST SRM612 and the two Byzantine glass weights BM S322 and BM 1983,1108.1 obtained in the big cell and the standard Resonetic S155 analytical cell. The deviation between the two analytical conditions generally remains below 10%, but exceeds 20% at low concentrations of light elements, where the sensitivity of the big cell is notably compromised.

The majority of the glass weights was analysed using a standard Resonetic S155 analytical cell with a maximum size of 15 x 11 x 3 cm. Objects that exceeded these dimensions were analysed in a new purpose-built cell designed for the study of large objects (up to 40 x 40 x 13 cm). As this cell was used here for the first time, the spectrometer tuning parameters had to be optimised to strike a compromise between sensitivity, background intensity level and stability of the signal. To ensure the comparability of the data between the two cells, two of the glass weights analysed in the standard S155 cell were analysed also in the large cell alongside the reference material (Corning A, NIST SRM612). Comparison of the results (Fig 2) shows very good agreement for oxides with concentrations above the ppm level (0.9 > [Large cell]/[Cell S155] > 1.1). However, the results for some elements clearly diverge (mainly Li, B, P, Cl, Cr and to a lesser extent Sn), possibly as a result of a loss in sensitivity for light elements and a considerable increase in the background level for some masses (Cr). These two factors drastically increase the detection limits for chromium and phosphorus.

Ten diagnostic base glass element oxides (Na, Mg, K, Ca, Al, Fe, Ti, Zr, Sr, B) of the main group of Byzantine glass weights (n = 255, excluding all heavy weights and outliers) were subjected to multidimensional principal component analysis (PCA), using Matlab version 2015b. The principal component scores were calculated directly on the mean-scaled LA-ICP-MS data. Principal components 1 and 2 account for almost 60% of the variability and allowed for the separation of the data into distinct primary production groups.

## Results

**Fig 3:**
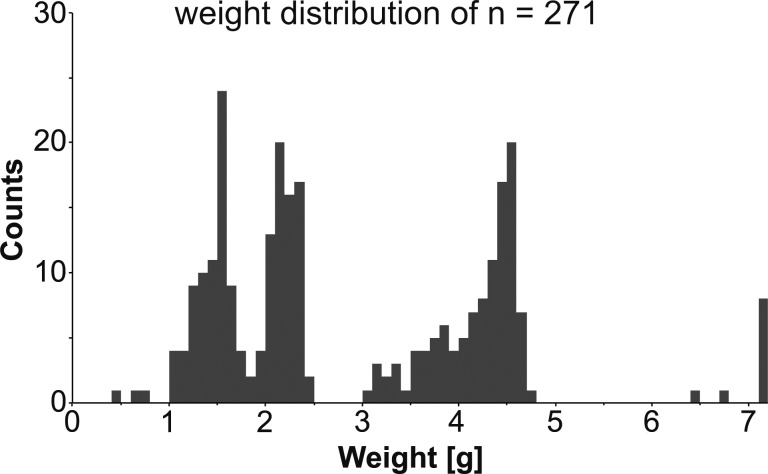
Histogram of the mass of 271 Byzantine glass weights. The mass distribution of Byzantine glass weights from the British Museum (BM) and the Biblothèque nationale de France (BnF) coincide roughly with the weight values of Byzantine gold coinage: 4.5 g (nomisma), 2.25 g (semissis), 1.5 g (tremissis). Data were binned at 0.1 g intervals.

The distribution of the masses of the analysed glass weights is consistent with the expected weight standards for Byzantine gold coinage, showing clear peaks at around 4.5 g (nomisma), at 2.25 g (semissis) and at 1.5 g (tremissis) (Fig 3). However, it is inherently difficult to assign modern units to historical artefacts and it can be assumed that the actual weight of Byzantine coinage fluctuated within an accepted margin of tolerance and that the glass weights reflect this variability [31, 49]. Hahn and Metlich proposed that a deviation of 47 mg for a gold coin was tolerable [32], while one of the present authors advocates a more precise value of 20 mg [35].

Interestingly, the distribution is skewed towards lower values relative to the standard coin units. Each of the main peaks has a long tail to the left, whereas there is an immediate drop to the right. This effect could be related to weight loss due to corrosion and damage, the attempt to prevent the underestimation of the value of gold coins, or the precision of the production process. One theory, first discussed by Matson [50, 51], assumes that Islamic glass weights were made by separating molten glass dollops of about the right size and imprinting them with a die while still hot, creating the typical bulging, rounded rim. Weights produced in this way would have deviated from the intended standards and those too far removed could then have been immediately recycled in the furnace [33]. Although experienced glassmakers are able to come close to the desired weight, the scrap rate must have been rather high considering the low weights and minor differences between, for example, the tremissis and semissis. Another possible scenario is that glass chunks were weighed in cold condition and then melted and imprinted with a die as soon as viscous. In this way, an exact weight could be guaranteed and recycling would be limited. A total of 10 glass weights exceed the expected weight standards outlined above, ranging from 6.66 g (BM 1984,0108.2) to as high as 314 g (BM 1986,0602.1) (S1 Table). The two weights with masses of just over 6 g might, in fact, have been used for hexagramme silver coins minted during the reign of Heraclius [35].

**Fig 4:**
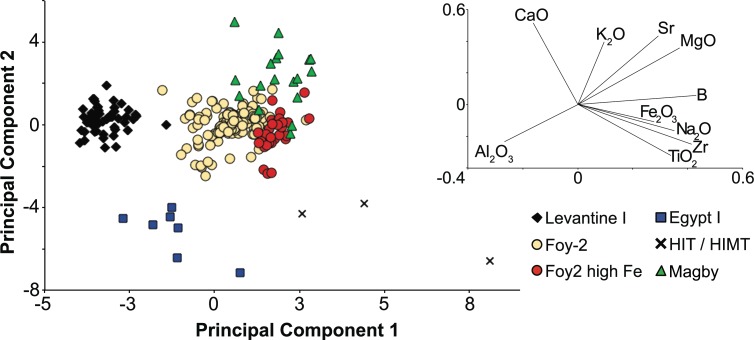
Principal component analysis of LA-ICP-MS data. Six compositional groups can be separated based on silica-derived elements (Mg, K, Ca, Al, Fe, Ti, Zr, Sr) as well as varying concentrations of Li and B that are associated with the fluxing agent. Principal components 1 and 2 amount to approximately 60% of the overall variability. The contribution of each element to PC1 and PC2 is indicated by the vectors (represented on an enlarged scale).

Excluding the heavy weights and the outliers that obscure the group affiliations, the Byzantine glass weights can be attributed to 6 different compositional groups (S1 Table, Fig 4): Levantine I, Foy-2 (including série 2.1 and série 3.2), Foy-2 high Fe, Egypt I, HIMT and a group designated as Magby due to its notable magnesia levels (Magnesium Byzantine glass). All groups have a soda-lime-silica composition, five of which correspond to a natron-type base glass. In contrast, the elevated magnesia and potash levels of the Magby group indicate the additional use of plant ash rather than mineral soda alone. The compositional differences relate generally to the primary glassmaking components, such as aluminium, calcium, iron, potassium, magnesium, strontium, titanium and zirconium reflective of the silica source, as well as boron and lithium that are primarily related to the fluxing agent (Fig 4). Further variations were found in relation to the silica to soda ratio and the cobalt source. In addition to these six groups, there are 12 outliers that will be discussed separately and that are not included in the binary graphs.

**Fig 5:**
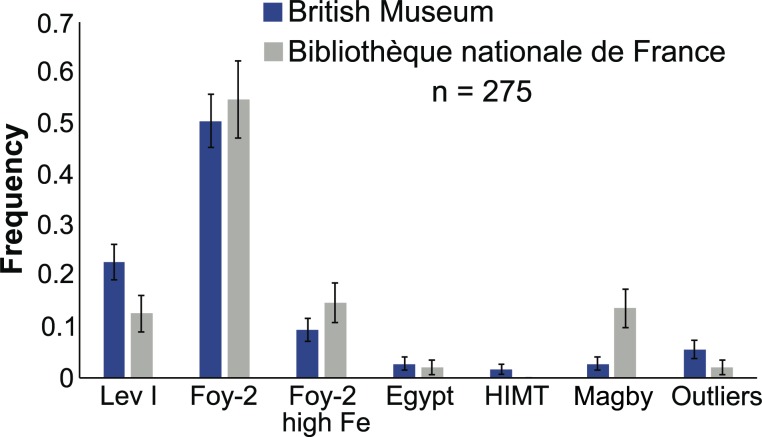
Frequency of the different production groups represented among the BM and BnF collections. The distribution of artefacts according to compositional categories is similar in the two national collections, demonstrating that this is a representative cross-section of known Byzantine glass weights. Error bars indicate the standard deviation derived from the variance of a Poisson distribution.

The largest group by far, representing more than 50% of both the BM as well as the BnF assemblages, is the Foy-2 compositional group, followed by Levantine I that makes up approximately 22% and 12.5% of the BM and BnF collections, respectively (Fig 5). Only a limited number of Egypt I (2–3%) was identified, and HIMT was detected solely in 4 samples from the BM. There is a discrepancy between the BM and the BnF collections with respect to the Magby group. Whereas Magby glasses make up less than 3% of the BM glass weights, about 13.5% of the BnF weights can be attributed to this category. Overall, however, there is a very good correspondence between the two assemblages, making this a comprehensive study of Byzantine glass weights in terms of their glass compositions, weight values and typologies.

### Levantine I (n = 53 samples)

**Fig 6:**
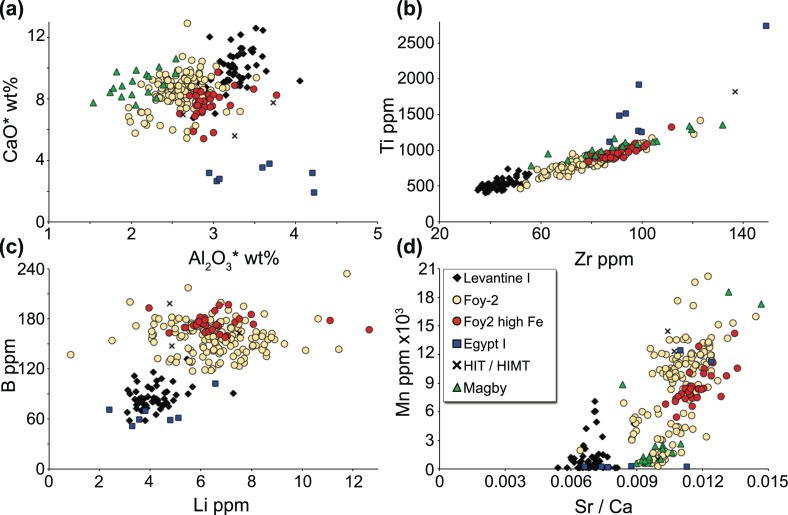
Base glass characteristics of the identified compositional groups. (a) Lime and alumina concentrations of the Byzantine glass weights (excluding outliers and large weights, S1 Table) indicate differences in the silica source. Asterisks indicate reduced and normalised data. (b) Correlation between zirconium and titanium. (c) Different lithium and boron levels suggest differences in the natron source. Note that the Magby group is not included in this graph due to the significant plant ash component. (d) Strontium to calcium ratios point to different strontium concentrations in the carbonate fraction of the silica source. The correlation between Mn and Sr/Ca provides evidence that manganese minerals present an additional but highly variable source of strontium in the Foy-2 and Foy-2 high Fe glasses.

The Levantine I group consists of mostly naturally coloured blue-green / ‘aqua’ glass as well as cobalt blue specimens. Levantine I has been widely recognised among glass finds from the fourth/fifth to the seventh century CE [6, 9, 17, 21, 52–54]. It is a fairly coherent group with relatively high alumina and lime concentrations, alongside low contaminations with heavy minerals such as titanium, zirconium or iron oxides (Figs 4 and 6). Compared to the Foy-2 group, the Levantine I samples have lower magnesium, boron and lithium levels as well as lower strontium to calcium ratios (Fig 6C and 6D). They generally contain no or low quantities of manganese (MnO < 0.5 wt%). The low levels of manganese support a sixth- to seventh-century date for these glasses, because earlier Levantine I assemblages typically contain higher quantities of manganese (e.g. [9, 17, 20, 21, 52, 54–56]). In fact, a substantial number of Byzantine glass weights with a Levantine I composition can be dated to the late sixth and early seventh century CE based on their inscriptions and design (S1 Table).

### Foy-2 (n = 143)

Foy-2 as here defined corresponds to the Foy-2 group from Carthage [20] and includes série 2.1 and série 3.2 as originally described by Foy and colleagues [17]. This type of glass has since been variably re-branded HLIMT [55], weak HIMT [57], and HIMT 2 [53, 58]. However, it has recently been shown that an association of Foy-2 with HIMT glass is misleading [20, 55, 59–61]. We therefore refer to this group as Foy-2. A fifth-century date has been proposed for the série 3.2 sub-type [6, 56, 59], and a sixth-century date for série 2.1 [20, 55, 59]. While the majority of Foy-2 glass weights are indeed attributed to the sixth century, a significant number of the weights date to the seventh century, confirming that production and circulation of Foy-2 (série 2.1) extended into the seventh (S1 Table).

**Fig 7:**
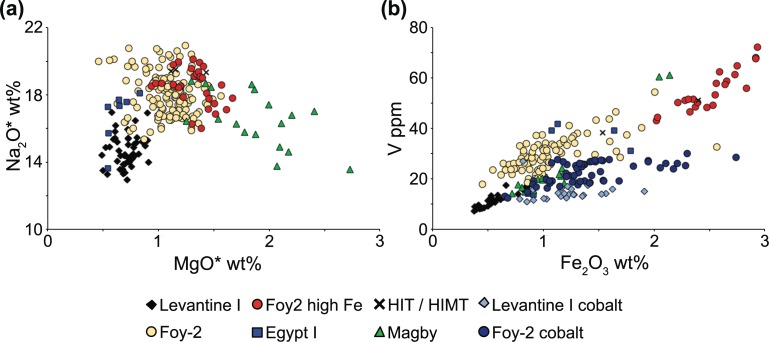
Soda, magnesia and iron oxide variations. (a) The higher soda content in Foy-2 and Foy-2 high Fe possibly reflects a closer proximity to the mineral soda deposits in Egypt, whereas the elevated levels of magnesia in the Magby samples indicate the addition of a plant-ash component. Asterisks indicate reduced and normalised data. (b) Correlation between vanadium and iron of the six glass groups with the cobalt samples singled out.

Foy-2 is the largest compositional group among the analysed Byzantine glass weights and encompasses colourless to greenish glass as well as amber, purple, dark green and cobalt blue samples. It is a relatively heterogeneous group, but tends to have lower lime and alumina contents than Levantine I glass, even though there is some overlap (Fig 6A). Foy-2 separates from Levantine I more clearly on grounds of higher magnesium and sodium concentrations (Fig 7A) as well as higher levels of the heavy minerals titanium, zirconium and iron (Figs 4, 6B and 7B). Trace elements associated with iron such as vanadium and lanthanum are increased, too. Noteworthy are the higher strontium to calcium ratios, reflecting a different lime and by extension different silica source compared to the Levantine I group (Fig 6D). Slightly higher boron and lithium levels are most likely indicative of variations in the natron source (Fig 6C). Manganese is highly variable and ranges from as little as 0.02 wt% (BM 1983,1108.4) up to 2.6 wt% (BM 1980,0611.24) where it expresses a purple colour (S1 Table). Since the base glass composition of these samples is otherwise very similar, implying a shared provenance, the Foy-2 group was not further sub-divided (compare e.g. [62]), with the exception of a group of glasses with high iron concentrations.

### Foy-2 high Fe (n = 31)

**Fig 8:**
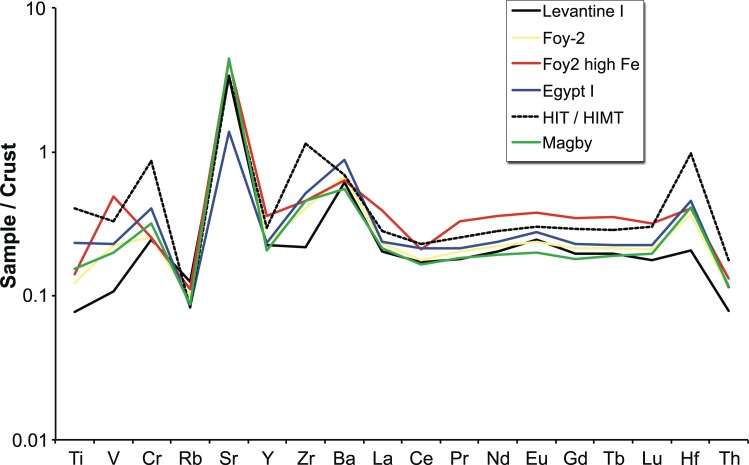
Trace element patterns of the primary production groups. Average trace element concentrations for the six production groups, normalised to the mean values in the continental crust [65].

The Foy-2 high Fe samples resemble the sixth-century high iron group from the Lower Danube [59]. It closely matches the Foy-2 group in almost all aspects, except for its significantly higher iron, vanadium and lanthanum contents (Fig 7B). The Foy-2 high Fe group displays slightly different trace element patterns to the Foy-2 glass type (Fig 8). This is significant, because rare earth elements (REE) do not typically differ between the known first millennium primary glass production groups (e.g. [63, 64]). Although some contamination from crucible clay cannot entirely be ruled out, the effects of a typical glass-melting pot with about 7 wt% Fe_2_O_3_ and 1 wt% TiO_2_ (e.g. [59]) on the titanium concentrations of the glass melt would be far more pronounced than what we see in the Foy-2 high Fe group. Hence, the elevated REE traces of the Foy-2 high Fe glasses compared to all other groups including Foy-2 indicate the use of a different yet related silica source. The Foy-2 high Fe samples furthermore display a very distinct colour that can be described as olive or yellowish green, with the exception of one amber, one aqua and one bluish sample (S1 Table, Fig 1A). A mid to late sixth-century date can be firmly established for some of the Foy-2 high Fe weights (S1 Table). Its production is thus more or less contemporary to the Foy-2 main group.

### Egypt I (n = 7)

Seven samples are compositionally consistent with an Egypt I production group [23], due to their very low lime (< 4 wt%) and relatively high alumina (3 wt%– 4 wt%) concentrations. Given the small number of samples and considerable variability, the composition of this group cannot be closely defined. On the whole, the Egyptian samples have magnesium, lithium and boron contents comparable to those of the Levantine I group, elevated heavy minerals like titanium, zirconium and hafnium similar to or higher than Foy-2, while displaying the lowest concentrations of rubidium and strontium (Figs 4, 6 and 8). The samples of this group are either bluish-green or purple and no firm date can be ascribed (S1 Table).

### HIT & HIMT (n = 3)

Three glass weights from the BM can be classified as HIMT (High Iron, Manganese and Titanium) or HIT (High Iron and Titanium) glass (série 1 [15, 17–19]). The samples have been clustered together on account of their high aluminium and sodium contents, in addition to exceptionally high iron, titanium, zirconium, chromium, and hafnium levels (Figs 4, 6, 7 and 8). Manganese varies between 0.13 wt% and 1.86 wt% and is taken here, as in the case of Foy-2, to be an optional component that does not necessarily betray differences in the silica source (see e.g. [66]). The HIMT/HIT glasses share with the Foy-2 group the strontium to calcium ratios as well as their boron and lithium concentrations (Fig 6C and 6D). The weights are olive green, green and cobalt blue.

### Magby glasses (n = 18)

The assemblage from the BnF in Paris comprises several weights that have been produced from glass that is clearly distinct from the other groups. This group is defined based on the magnesium and potassium oxide levels approaching and in excess of 1.5 wt% (Fig 7A), which is commonly seen as cut-off line between natron-type glass and glass produced from a plant-ash based flux [67]. The increased phosphorus concentrations and the correlation between the three elements are concurrent with the use of a plant-ash component (S1 Table). At the same time, the Magby glasses have the lowest concentrations of alumina, while they exhibit levels of titanium, zirconium, hafnium and iron similar to the Foy-2 group (Figs 4, 6 and 8). With the exception of three amber coloured samples, the manganese levels remain below 0.4 wt% (S1 Table, Fig 6D). The other colours represented among this group are bluish aqua, yellowish green and cobalt blue. Typological evidence points to a late sixth- to early seventh-century date for this group (S1 Table).

### Outliers (n = 12)

Twelve outliers are excluded from further discussion based on their chemical composition and/or iconographic features (S1 Table). Judging from the exceptional levels of arsenic (> 2 wt%) or chlorine (≤ 0.1 wt%), four glass weights in the BM collection were excluded as modern (possibly eighteenth- or nineteenth-century) copies (BM 1983,1108.7° & 8°, BM 1980,0611.60, BM 1980,1204.3°).

Two samples (BM 1980,0611.35 & 40) with very similar cruciform monograms have unusually high potash concentrations (4.25 wt% and 5.95 wt%). Neither magnesium, phosphorus or calcium oxides are particularly increased. Since the use of wood ash usually results in higher values for these elements, it is not entirely clear what might have caused the high potassium contents. In contrast, the addition of plant ash presumably underlies the elevated magnesium, phosphorus and potassium levels of two further glass weights (BM 1981,0601.6, BM 1986,0406.20). One sample is a high lead glass (Pb > 70 wt%), while one specimen (BnF Froehner 44) has exceptionally high alumina contents (9.05 wt%) coupled with elevated boron (0.24 wt%). Except for its comparably low lime and high potassium levels, this glass resembles the high alumina coloured samples identified amongst the eighth- to ninth-century Byzantine glass fragments from Pergamon [7, 68]. The last two outliers are of an indistinct design. Sample BnF Schlumberger 3997 appears to correspond roughly to a Levantine I glass composition despite its low alumina concentrations. BM 1983,1108.4, in contrast, has high contents of titanium, zirconium and hafnium, linking it to an Egyptian origin. In its major and minor element make-up, namely its relatively low potassium and aluminium oxides, high lime and moderate soda, this glass corresponds to the Egypt II group of Gratuze and Barrandon [23], the glasses from the eighth- to ninth-century Islamic workshop at Tel el Ashmunein in Middle Egypt [14] and some seventh- to eighth-century glass working debris from Tel Aviv (group C in [69]). Similar to the Tel el Ashmunein glass, the Byzantine glass weight has low strontium contents (166 ppm), indicative of limestone-bearing sand [2]. This outlier is part of the so-called ‘Arab-Byzantine’ weights that were linked to Coptic merchants [27, 28].

### Large weights (n = 8)

Of the eight large, heavy glass weights, one square sample with a gamma and alpha mark (BM 2002,1001.1) agrees with the Levantine I type (S1 Table). Due to its similarities with the large weights found in one of the shops on Monument Street in Bet Shean that were destroyed by fire around 540 CE ([39] p. 449), it presumably dates to the first half of the sixth century CE. The low soda content (12.5 wt%) may be due to heavy surface weathering. Another large square-shaped weight (BM 1946,0406.1) with a central cross and various Greek letters in low relief is a HIMT glass with unusually high zinc levels (237 ppm). All other heavy weights are of the Foy-2 type, covering virtually the full range of compositional variations typical for this glass group. They are all decorated with one or more box monograms. No colorants or decolorants other than iron and manganese were detected at sufficient levels to suggest their deliberate addition, resulting in a natural greenish colour.

### Colorants

Surprisingly few intentionally added colorants were detected. Most colours such as aqua, bluish, yellowish and olive green are the result of the presence of iron at various concentrations and oxidation states. Manganese is responsible for purple and in combination with iron for some of the strong amber colours. The manganese of the Foy-2 glasses is associated with barium and strontium, providing evidence that these trace elements were not introduced exclusively with the feldspars or the carbonate fraction of the silica source but with the manganese-bearing mineral as well (Fig 6D). Copper at levels indicating its intentional use as colorant (> 0.5 wt%) was detected in only 3 samples: 1 turquoise specimen (BnF Schlumberger 3997), 1 green sample (BM 1980,0611.69), 1 weight with red streaks (BM 1980,0611.64). Copper tends to be slightly elevated in the cobalt blue glasses (S1 Table).

**Fig 9:**
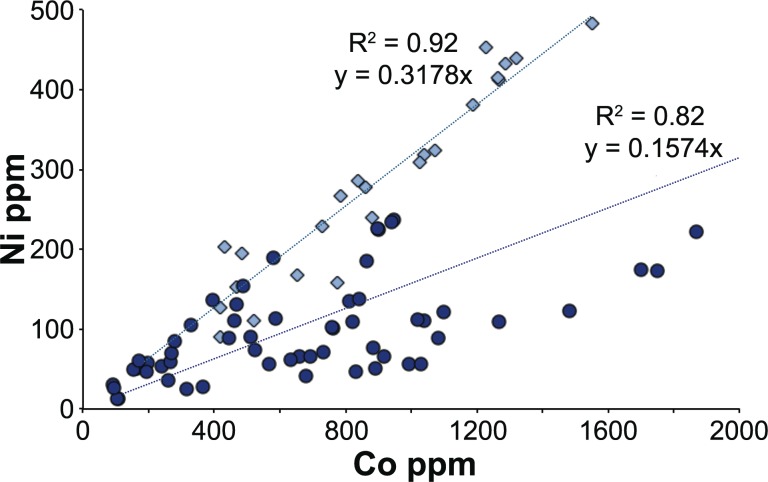
Chemical characterisation of the cobalt colorant in Levantine I and Foy-2 glass weights. Cobalt concentrations are correlated with nickel to a varying degree in Levantine I and Foy-2 glasses. The high correlation coefficient of the linear regression of the Levantine I group reflects the use of a single, homogenous cobalt source.

82 glass weights are dark cobalt blue, with cobalt contents ranging from about 90 ppm to 6000 ppm (S1 Table). The nature of the cobalt colorant between the Levantine I and the Foy-2 groups differs in terms of its correlation with nickel. The cobalt of the Levantine I glasses is very homogeneous and appears to derive from a single source. It is strongly correlated with nickel and to a lesser extent with molybdenum, indium and lead (Fig 9). Some of the blue Foy-2 samples show the same correlation as the Levantine I group, however, the cobalt colorant here is more diverse, resulting in a different correlation between cobalt and nickel.

### Batches

**Fig 10:**
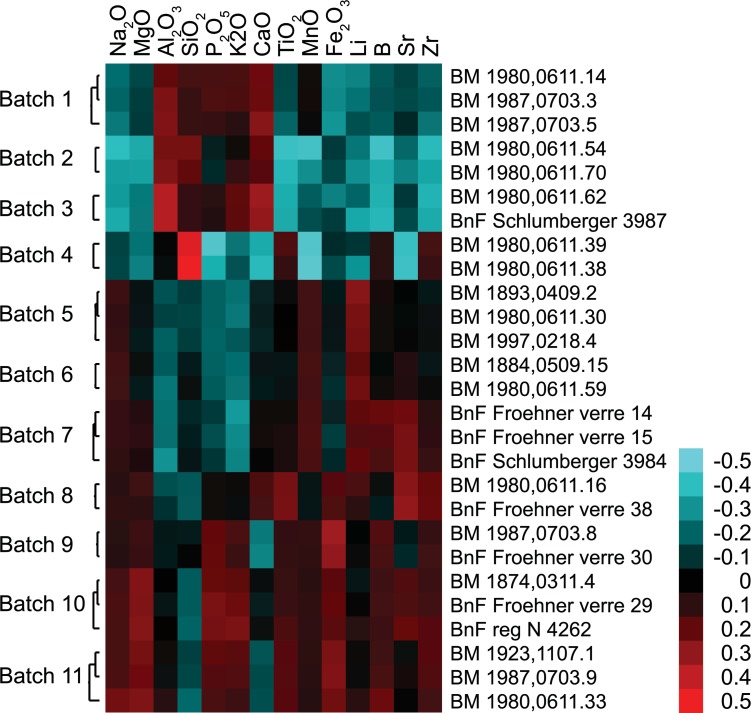
Hierarchical clustering reveals production batches. Batches were identified by hierarchical clustering with the programme Cluster 3.0 [71], and visualised using Treeview [72]. Pearson correlation (uncentered, with average linkage) was carried out on log_2_-transformed, mean-centered data that had been normalised for each element, and are shown on a false colour scale (right). Samples belonging to the same batch display highly correlated compositional profiles.

**Fig 11:**
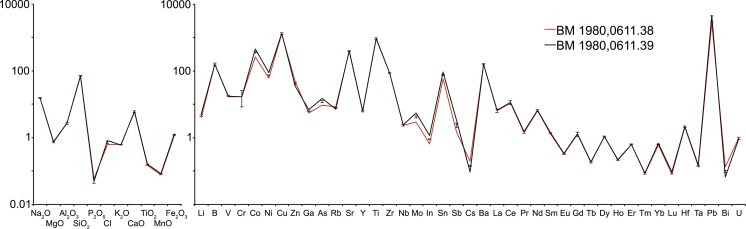
Trace element patterns of two cobalt blue samples of batch 4. The two cobalt blue samples (BM 1980,0611.38/red; BM 1980,0611.39/black) have identical base glass compositions, while minor variations in Co, Ni, Mo and As relate to the cobalt colorant. Concentrations are given as wt% (left) and ppm (right panel) on a logarithmic scale. Error bars reflect twice the relative standard deviation of the Corning A standard measurements applied to the average value of the two samples.

Glass samples can be considered to belong to the same batch or melting event if the concentrations of all elements measured are within the experimental error [69, 70]. Groups of glass weights with iconographic similarities were thus compared and a number of batches identified where the compositional data of the different samples were within two standard deviations of the repeated measurements of Corning A and NIST 612. Additionally, hierarchical clustering was performed that confirmed the batches and revealed several more highly correlated clusters. Altogether, at least eleven very tight compositional batches were thus identified, each containing two to three individual weights that are highly correlated across 58 elements analysed (Fig 10). Minor variations shown in cobalt and associated elements within the same batch provide evidence for the addition of the colorant at the secondary working stage (Fig 11).

## Discussion

### Raw materials and provenance

The compositional groups of the Byzantine glass weights are typical of late antique glass assemblages and differ in their raw materials, most notably silica, soda and calcium sources. Silica sources have traditionally been differentiated based on the lime and alumina contents (e.g. [69, 70]). However, it becomes increasingly clear that in order to define group affiliations and differences between primary production groups a multi-dimensional approach is needed that takes into account several components and can resolve partial overlaps (e.g. [20]). The examination of minor and trace element patterns such as aluminium, titanium, zirconium and strontium demonstrates that the different production groups were made using silica sources with a distinct mineralogy. While aluminium reflects the presence of feldspars, titanium and zirconium represent the heavy minerals and strontium mostly the carbonate fraction in the sand. Levantine I glasses are on average richer in feldspars and poorer in heavy minerals than the other compositional groups. We believe that taken together, these compositional characteristics point to an Egyptian origin for all but the Levantine I silica sources and by extension to a primary production located in Egypt. This is further substantiated by the higher soda content of these glasses that may reflect the local availability of natron in closer proximity to the well-known deposits in the Wadi Natrun [69].

The strontium to calcium ratios suggest the use of shell as the source of lime in all the natron-based glasses in this study [2, 69]. The difference between the Levantine I and the Foy-2 groups indicate that shells with different strontium contents dominated the respective silica sources. The unusually high concentrations of strontium oxide in some of the Foy-2 samples (up to about 1000 ppm) may in part be related to the presence of manganese. A correlation of strontium and manganese has been observed elsewhere (e.g. [59, 73]). Nonetheless, given that even the low manganese Foy-2 samples have higher strontium to calcium ratios compared to the Levantine I group, the strontium bearing component of the silica source must necessarily vary between the two glass groups. While the strontium signature of Egypt I is consistent with Levantine I, that of HIMT and Magby glasses are closer to the Foy-2 group.

Judging from the boron and lithium contents, the flux used for the Levantine I production group differs from that of the Foy-2, HIMT and Magby groups, but is related to the natron of Egypt I (Fig 6C). Although some contribution of the silica source to the final concentrations of boron and lithium cannot be excluded, the two elements derive predominantly from the flux due to their chemical affinities (e.g. [7, 74]). This implies that the differences in the boron and lithium levels indicate variations in the natron source. While these discrepancies could result from natural variations in the mineralogical composition of the evaporite deposits that change through the seasons and vary between different lakes as well as within the same lake [74–76], it is perfectly plausible that natron from more than one source with different lithium and boron signatures was exploited. Possible sources close to the Wadi Natrun are the deposits at al-Barnuj in the western Nile Delta and nearby Nitria where natron is known to have been collected in the fourth century CE [75, 77]. However, variations in the natron sources of first millennium primary production groups, particularly their absolute boron and lithium values have not been sufficiently differentiated to draw any further historical or archaeological conclusions.

One of the interesting finds is the Magby group with elevated magnesium, potassium and phosphorus concentrations. Values of potassium and magnesium in excess of 1.5 wt% are commonly taken as an indication of the introduction of organic material in the form of plant or wood ash. Hence, there is good evidence that the Magby glasses were produced using a plant ash in addition to or instead of natron as the fluxing agent. It is thought that the change from natron to plant ash occurred in the early ninth century CE in Syria-Palestine and possibly slightly later in Egypt [23, 75, 78, 79]. Earlier glasses with compositions indicative of the addition of plant ash have since been identified, for example, in some emerald green glasses from first-century France and Britain [80], first- to third-century CE vessels from Egypt [57, 77, 81, 82], Byzantine glass from Caričin Grad / Iustiniana Prima in Serbia [1], as well as sixth- to seventh-century Anglo-Saxon glass from the British Museum collections [60].

The incidence of plant ash in some Anglo-Saxon glass has been interpreted by Freestone and colleagues [60] as the result of mixing natron-rich material with plant-ash or wood-ash rich material. Specifically, the authors proposed that an ash-rich material was added to the available (Period I) glass to augment a limited glass supply. The situation with respect to the Magby glass weights from Byzantium is somewhat different. A comparison of the compositional data of the Foy-2 and the Magby glasses suggests that they are closely related in terms of the silica source, including the strontium bearing components of the glass melt. The aluminium contents of the Magby samples, however, are on average significantly lower, ruling out a simple mixture of plant ash and Foy-2 glass. The negligible contamination with colorants (Co, Cu, Zn, Sb, Pb) suggests limited recycling, even though it does not exclude recycling processes as such [60]. Instead, the Magby glasses were presumably manufactured from a different silica source, richer in quartz and poorer in feldspars. Judging from their titanium, zirconium and hafnium profiles (S1 Table; Figs 6 and 8), their production location is likewise to be sought in Egypt.

The second- to third-century plant-ash glasses from the Wadi Natrun have a very distinct composition rich in lime (ca. 9.5 wt%– 16 wt%) and alumina (ca. 4 wt%– 6.7 wt%) and low soda levels (ca. 9.5 wt% - 12.5 wt%) and thus offer no viable comparison for our Magby glass weights [82]. Compositionally, the Magby weights seem closest to the four plant ash glasses reported from Bubastis and dated, with the exception of one sample, to the first to second century CE [57], and to the two sixth- to seventh-century specimens from Caričin Grad / Iustiniana Prima [1]. Whereas the earlier Bubastis glasses tend to have lower lime and higher manganese concentrations, the Serbian samples and Bubastis PA 04 (first to fifth century), resemble the Magby glass weights in most respects. The Byzantine weights have marginally higher titanium, iron and strontium levels. Strontium in Syria-Palestinian soda-rich plant ash glasses is typically in the order of 400–600 ppm [2]. In contrast, the strontium levels of the Magby glass weights range from approximately 480 ppm to 900 ppm and is comparable to the Foy-2 group. What this reveals is that the Magby glass weights were produced from a silica source with Egyptian characteristics, an alkali that is rich in magnesium, potassium as well as lithium and boron and a strontium to calcium ratio pointing to the use of either shell or an additional wood ash component.

### Recycling and secondary production

One of our working hypotheses was that the majority of weights might have been produced from recycled glass due to the low quantities of glass needed for their production. The unintentional introduction of colouring or decolouring elements with concentrations between 100 and 1000 ppm for lead and copper and > 30 ppm for antimony have been considered diagnostic for recycling (e.g. [3, 83–90]. In addition to the increase in colouring agents, more extensively recycled glass tends to accumulate elements associated with fuel ash, primarily potassium and phosphorus [20, 85, 91].

The different compositional groups of the Byzantine glass weights indeed exhibit signs of recycling with lead, copper and antimony usually between 100 and 200 ppm and elevated potassium and phosphorus. It appears, though, that while some recycled material was incorporated in most cases, including the large weights, the degree of recycling remained limited, especially as regards the Levantine I and Egypt I glasses. Antimony contaminations, for example, are almost exclusively found in the Foy-2 and Foy-2 high Fe groups and only in a handful of Levantine I samples. The geographical pattern of antimony as a marker for recycling is informative in other ways. The higher rate of antimony in what we believe to be Egyptian glasses might reflect the relative abundance of antimony-decolourised material available for recycling, thus substantiating an Egyptian origin for Roman antimony decolourised glass [20, 57, 85]. The mixing of antimony into the Foy-2 glass at low levels either implies a location for the secondary production of Byzantine glass weights of this composition in a place where both types of glasses were readily available, or that antimony-decoloured glass cullet had been used as a starter material during the primary manufacture of the Foy-2 type glass. The addition of scrap glass to a batch of raw materials has been previously proposed as it lowers the overall melting temperature, meaning less fuel expenditure ([92, 93] p. 39).

The overwhelming presence of Foy-2 among the BM and the BnF assemblages, and its compositional and chronological spread, supports the view that weights produced from this type of glass were made over a longer period of time and/or at multiple sites. The Levantine I group is generally much more homogeneous. Whether or not these variations are related to any specific Byzantine mint or the financial reforms is difficult to tell, but worth considering. The greatest changes in the production of Byzantine glass weights seems to have coincided with the changes of the coin mints that began towards the end of the reign of Phocas (602–610 CE). The late antique administrative structures started to disintegrate and the decentralised organisation of the financial system of the late sixth century was abandoned in favour of a more centralised administration, especially after the reforms of Heraclius in 629 ([94] pp. 501ff; [95] p. 414). As a consequence, almost all of the provincial mints were closed in the east and only Constantinople remained active. In the west, on the other hand, the mints of Ravenna and Carthage continued to work [96]. If the production of glass weights was indeed intimately connected to the imperial mints, then the centralisation of the financial system might explain the relative homogeneity of the Levantine I weights that are generally attributed to the later part of the sixth and early decades of the seventh century.

### Batches and dies

At least eleven batches were identified across the three main compositional groups Levantine I, Foy-2 and Foy-2 high Fe that encompass the different typologies: box and cruciform monograms, busts and inscriptions (S1 Table, Fig 10). In most cases, the batches correspond to weights on which the same or a similar die was used. Batch 1 is consistent with a Levantine I composition and includes three pale bluish green weights from the BM with very similar but not identical box monograms that resolve to ‘of Pakianos’. Judging from the elevated copper and lead concentrations, this batch was produced from recycled cullet rather than raw glass.

Different dies were used for the weights belonging to batch 11. The olive green weights produced from Foy-2 high Fe glass are all imprinted with a cruciform monogram that reads ‘of Timotheos’, but the ‘M’ and the ‘E’ on the crossbar are inverted (Fig 1A). This provides strong evidence that more than one die was employed in the same workshop at the same time. Strictly speaking, batch 11 could be merged with batch 10. The two batches differ mostly in their degree of contamination with lead and antimony of the otherwise identical base glass compositions (S1 Table). The explanation is likely to be that these minor variations resulted from the introduction of small amounts of recycled material to the same batch. Intriguingly, one of the weights (BnF reg N 4262) belonging to batch 10 bears the imprint of a very different die. This example has a bust and an inscription that reads ‘Kyrionymos’, illustrating once more that different dies were used in the same workshop concurrently.

The reverse was also found where the same die was used in connection with different base glasses, pointing to the use of different glasses and colours in the same secondary workshop. An example of this can be seen in two glass weights from the BnF (Schlumberger 3995 & Froehner verre 19) with the bust of an eparch holding a mappa in his right hand and an inscription encircling the bust that reads ‘Neilos’ (Fig 1E). As far as can be discerned from the worn imprints, both weights appear to have been stamped with an identical die. Whereas the Schlumberger 3995 weight has an unadulterated Levantine I signature, Froehner 19 is manufactured from a Magby glass that likewise shows no signs of recycling. Where these glass weights were manufactured must remain uncertain, but given the pristine quality of both the Egyptian and the Levantine material, we can exclude Egypt as well as the Levant and tentatively propose one of the other imperial mints in Carthage, Ravenna or Constantinople.

### Cobalt-blue pigment

Some of the identified batches bear witness to the addition of the colouring agent during the secondary working stage. The two cobalt blue samples of batch 4 (BM 1980,0611.38 & 39), for example, resemble each other in terms of the cruciform monogram that signifies ‘of Ioulianos’ and their base glass composition, but exhibit minor variations in elements associated with the colouring agent. More specifically, there is a slight discrepancy in their cobalt, nickel, arsenic and molybdenum concentrations (Fig 11). This has two fundamental implications: first, the cobalt colorant was evidently added at the secondary production stage and was not uniformly distributed throughout the glass batch; secondly, the differences in the trace element patterns identify those elements that are associated with the cobalt source. This means that the identification of batches among artefact assemblages not only gives insights into workshop practices but potentially also the geochemical nature of additives such as colorants.

The cobalt blue samples have on average higher concentrations of iron oxide compared to the non-coloured glasses of the same group. The elevated levels can to a certain extent be explained by the addition of an iron-containing cobalt ore such as skutterudite, where cobalt is associated with nickel, iron and arsenic ([97] p. 69–74). That the excess of iron in the cobalt blue weights is independent of the silica source is exemplified by the altered correlation between iron and vanadium (Fig 7B). Vanadium in the cobalt blue samples does not increase to the same degree as a function of iron as in the other glasses. This variance in the iron to vanadium ratio is indicative of a different type of iron compound and by extension a difference in the iron as part of the silica source versus iron associated with the cobalt-bearing mineral. The ratio of iron to cobalt can be estimated by linear regression of the cobalt containing samples (approximately 6 Fe_2_O_3_ / Co). Generally, the ratios tend to be lower than those of Roman glass, suggesting that the cobalt colour for the Byzantine glass weights derived from a different mineral source [80, 98].

The nature of cobalt colorants has previously been characterised based on the trace elements associated with cobalt. In a study of western European glass Gratuze and colleagues established four distinct cobalt-bearing minerals with varying impurities that were used for the colouration of glass from the late Bronze Age through to the 18^th^ century [99, 100]. The majority of cobalt used prior to the twelfth century showed no distinct connection with any particular trace element, even though elevated levels of manganese, antimony and copper are often evident [100]. Cobalt associated with higher than normal impurities of nickel were identified among the fourth- to eighth-century Merovingian glass finds from Larina [99], the sixth- to seventh-century samples from Xanthos [101], and the seventh-century remains from a secondary glass workshop at Beirut [102], but no provenance for this group of cobalt has been proposed. The presence of nickel in the cobalt pigment has in the past been linked to the well-known cobalt mines at Qamsar in central Iran [103]. Due to the lack of systematic analytical data of the mineralogical and chemical composition of the cobalt deposits at Qamsar, no firm conclusion can be drawn at this point. Taken together, the trace element patterns of the Byzantine glass weights point to the exploitation of a cobalt deposit during the Byzantine period that differs from the Roman source of cobalt and is characterised by lower iron to cobalt ratios and elevated trace element contaminations, particularly nickel. Given the homogeneity of the cobalt used for the Levantine I glass weights, it can be assumed that the Byzantines had a more or less direct access to a specific cobalt source. Its strong correlation with nickel and similar compositional patterns in cobalt blue glass finds from other late antique sites in the eastern Mediterranean testify to a distinctly post-Roman, possibly specifically Byzantine, cobalt source.

## Conclusion—Byzantine glass

This study established the main patterns and developments in the production of early Byzantine glass weights based on about 20% of all known specimens (about 1,300 in total). Overall there is good agreement between the BM and the BnF assemblages in terms of the group distributions (Fig 5). Especially the relative quantities of Foy-2, Foy-2 high Fe and Egypt I are well within the margins of error. The lack of HIMT samples among the BnF assemblage can be explained by the stochastic nature of sampling, as the expected number would amount to only one sample. There is some discrepancy between the two collections as regards the Levantine I group, but the estimated standard deviation ($\sqrt{n}$) for the respective frequencies (*n*) are relatively close. Part of the difference might be due to different sampling strategies and histories of the two collections. This most certainly applies to the mismatch in the frequency of the Magby weights, which is not accounted for by statistical variations. However, given the relatively high chronological resolution and the significant number of samples, our study allows for statistically meaningful conclusions to be drawn about Byzantine glass more generally.

What of Byzantine glass as a distinct category and its raw materials then? Consistencies in the base glass composition and limited recycling indicate a continuous supply of raw glass, whilst inconsistencies in the cobalt colorant among the Foy-2 samples demonstrate a range of supply options for additives. The principal glass group used for the Byzantine glass weights throughout the sixth century was the Egyptian Foy-2 type alongside an iron-rich subgroup (Foy-2 high Fe). Towards the later part of the sixth century or the first decades of the seventh, this appears to be complemented by a related primary production group also from Egypt with higher magnesium levels (Magby). The use of Levantine I in the manufacture of Byzantine glass weights is similarly attributed to the late sixth and the first decades of the seventh century. These Levantine I weights reveal a much more controlled manufacturing procedure with little recycling and the exploitation of a single, novel cobalt source, indicating an increasing centralisation of the financial system.

The discovery of a substantial number of weights where the alkali source was a plant ash instead of mineral soda is somewhat surprising but not unprecedented. It is not clear why the change in fluxing agent from natron to soda-rich plant ash in the late eighth or early ninth century took place. Of the different factors that have been proposed, including political unrest in the Nile delta or increased demand for glass, environmental factors seem the most likely reason for the use of plant ash in the late sixth century [75, 82]. The extraction of natron from mineral deposits such as Wadi Natrun is susceptible to climatic fluctuations. Scientific data and contemporary written sources reveal major climatic instabilities in the sixth century, with an increase in annual precipitation and wetter conditions in the eastern Mediterranean during its last quarter [104–106]. An increase in humidity would have reduced the amount of natron available in the evaporitic deposits of the Wadi Natrun [75, 79, 82]. This may have inspired the use of plant ash to augment or replace mineral soda as observed in a number of sixth- to seventh-century Byzantine glass weights.

The comprehensive scope of our project and the firm chronological attribution of the glass weights give these observations high relevance, even though we cannot fully explain the developments in the production and use of Byzantine glass. There is still much to learn in terms of the chronological and geographical trends and the commercial processes through which secondary workshops were supplied with raw glass and additives. Unravelling these complex environmental, economic, geo-political and cultural developments requires an interdisciplinary approach as the one applied to this study.

## Supporting Information

S1 Table LA-ICP-MS data of the Byzantine glass weights. Major and minor oxides [wt%], including chlorine, and trace elements [ppm].
(PDF)
