# Supplementary material for: Comprehensive Chemical Characterisation of Byzantine Glass Weights
Source: PLoS One. 2016 Dec 13;11(12):e0168289. doi: 10.1371/journal.pone.0168289 (PMC5154553; doi:10.1371/journal.pone.0168289)
Supplement: S1 Table — Major and minor oxides [wt%], including chlorine, and trace elements [ppm]. (PDF) [file pone.0168289.s001.pdf]

S1 Table: LA-ICP-MS data of the Byzantine glass weights. Major and minor oxides [wt%], including chlorine, and trace elements [ppm]; where the monograms are ambiguous, multiple names are given (Tobias, forthcoming).

Sample Number	colour	type	names	weight [g]	Ø [mm]	Date	Na_2O	MgO	Al_2O_3	SiO_2	P_2O_5	Cl	K_2O	CaO	TiO_2	MnO	Fe_2O_3	Li	B	V	Cr	Co	Ni	Cu	Zn	Ga	As	Rb	Sr	Y	Zr	Nb	Mo	Ag	Cd	In	Sn	Sb	Cs	Ba	La	Ce	Pr	Nd	Sm	Eu	Gd	Tb	Dy	Ho	Er	Tm	Yb	Lu	Hf	Ta	W	Au	Pb	Bi	Th	U
Levantine I																																																														
BM 1872,1201.9	blue-green	bust & crosses		2.04	19.3	6th - 7th	13.3	0.64	3.26	69.7	0.10	0.94	0.59	10.7	0.08	0.02	0.46	3.68	82.0	8.57	4.86	1.80	4.42	7.80	8.48	4.29	2.47	8.85	544	7.23	38.7	1.50	0.28	0.04	0.01	0.02	4.82		0.08	226	6.63	12.5	1.59	7.07	1.44	0.38	1.37	0.20	1.17	0.25	0.69	0.09	0.63	0.08	1.02	0.09	0.04		29.8	0.01	0.81	0.66
BM 1879,0522.49	blue-green	bust & inscription	Rogatos	1.82	20.8	610/612	14.0	0.72	2.91	68.0	0.22	0.66	0.86	11.8	0.09	0.02	0.53	4.91	82.0	13.4	19.6	1.88	7.24	14.4	15.2	3.71	2.25	9.69	456	7.21	42.1	1.56	0.64	0.43	0.06	0.01	2.52	0.11	0.08	199	6.74	12.0	1.50	6.66	1.32	0.36	1.13	0.19	1.05	0.22	0.62	0.09	0.58	0.09	1.04	0.08	0.07	0.10	8.50	0.01	0.84	1.13
BM 1884,0509.14	blue-green	bust & inscription	Ioannos	1.45	17.4	6th - 7th	13.1	0.67	3.11	71.7	0.09	0.93	0.60	9.06	0.09	0.02	0.56	5.50	89.8	10.7	5.71	2.19	5.49	16.0	12.0	3.94	2.04	9.15	461	7.22	41.9	1.72	0.27	3.59	0.03	0.02	2.51		0.08	233	6.73	12.5	1.56	6.93	1.36	0.38	1.23	0.19	1.14	0.24	0.64	0.09	0.61	0.09	1.05	0.09	0.06	0.01	15.3	0.02	0.84	0.65
BM 1891,0512.13	blue-green	cruciform monogram	Akakios/Kaios/ Akakos	4.46	25.2	6th - 7th	13.3	0.49	2.78	73.8	0.08	0.96	0.64	7.41	0.08	0.02	0.43	3.18	58.3	8.14	6.56	1.40	3.84	6.86	8.14	3.81	2.03	8.86	378	6.37	44.1	1.44	0.36	0.19	0.04	0.01	0.75		0.09	209	6.07	11.2	1.44	6.13	1.26	0.34	1.19	0.18	1.00	0.21	0.61	0.08	0.55	0.07	1.12	0.08	0.07		5.15		0.76	0.58
BM 1893,0205.75	blue-green	box monogram & inscription*	Ioannos*	4.31	25.1	1. half 7th	13.8	0.71	3.20	70.3	0.12	0.92	0.62	9.52	0.09	0.02	0.58	3.91	98.3	11.7	15.1	2.15	6.29	7.19	10.7	4.05	2.92	7.45	453	7.15	36.9	1.51	0.51	0.06	0.00	0.01	0.81	0.02	0.06	267	6.15	11.4	1.49	6.61	1.31	0.40	1.44	0.19	1.14	0.23	0.67	0.08	0.61	0.08	0.92	0.09	0.05		7.06		0.79	0.68
BM 1896,0616.2	olive green	cruciform monogram		4.35	24.5	6th - 7th	13.9	0.74	3.02	69.2	0.20	0.65	0.89	10.2	0.12	0.13	0.77	4.31	79.0	13.9	7.63	3.97	7.62	42.8	22.9	4.15	2.92	10.2	440	7.09	51.2	2.00	0.56	0.13	0.04	0.07	16.3	5.12	0.10	212	6.79	12.4	1.53	6.69	1.35	0.38	1.24	0.19	1.14	0.22	0.62	0.08	0.60	0.09	1.23	0.11	0.07	0.05	90.4	0.02	0.94	1.21
BM 1980,0611.7	blue-green	box monogram	Eudaimonos / Methodios	4.22	23.8	6th - 7th	16.5	0.88	2.84	68.1	0.10	0.91	0.65	8.51	0.10	0.61	0.66	5.46	132	17.3	14.5	5.31	9.55	33.3	15.8	3.98	3.49	8.21	539	6.98	54.3	1.84	1.82	0.14	0.04	0.03	4.14	36.5	0.10	260	6.42	11.2	1.50	6.49	1.23	0.35	1.35	0.19	1.19	0.22	0.64	0.08	0.64	0.08	1.36	0.12	0.11	0.01	53.4	0.02	0.95	0.75
BM 1980,0611,14	blue-green	box monogram	Pakianos	1.33	17.7	6th - 7th	14.7	0.83	3.04	67.6	0.19	0.66	0.83	9.96	0.11	0.78	0.86	4.25	106	16.8	25.1	12.5	12.4	311	45.2	4.24	6.01	11.1	506	7.26	53.9	1.91	1.56	6.16	0.09	0.66	169	34.4	0.12	279	6.95	12.6	1.59	7.05	1.44	0.38	1.40	0.20	1.21	0.24	0.65	0.08	0.67	0.09	1.33	0.11	0.62	0.33	1297	0.18	1.01	0.88
BM 1980,0611.17	olive green	box monogram	Pakianos	1.72	17.8	6th - 7th	16.7	0.57	2.77	68.3	0.21	0.96	0.97	8.91	0.09	0.02	0.44	3.47	105	8.22	16.1	2.17	4.19	14.1	8.04	3.55	3.86	8.45	371	6.01	52.2	1.51	0.35	0.07	0.02	0.01	0.83		0.05	186	5.74	10.5	1.32	5.92	1.21	0.34	1.17	0.17	1.04	0.20	0.58	0.07	0.59	0.08	1.33	0.09	0.06		8.05	0.01	0.81	0.54
BM 1980,0611.18	blue-green	box monogram	Pelagios	2.09	19.5	6th - 7th	13.8	0.62	2.92	71.1	0.12	0.89	0.77	9.09	0.07	0.02	0.44	3.46	76.9	8.52	15.0	1.76	4.54	8.67	9.01	3.72	2.08	10.1	411	6.44	38.8	1.33	0.38	0.09	0.03	0.01	1.42	0.47	0.09	206	5.78	10.4	1.30	5.97	1.24	0.33	1.24	0.17	1.04	0.20	0.59	0.08	0.53	0.08	0.99	0.07	0.05	0.03	24.6	0.01	0.73	0.48
BM 1980,0611.21	blue-green	cruciform monogram	Philippos	4.33	25.2	1. half 7th	13.1	0.53	3.09	72.1	0.15	0.73	0.81	8.83	0.09	0.02	0.50	3.85	73.2	11.4		1.83	5.55	9.81	13.6	4.00	2.22	10.5	400	6.84	46.0	1.60	0.44	0.20	0.03	0.01	5.78		0.11	245	6.55	11.6	1.47	6.34	1.25	0.35	1.22	0.18	1.09	0.22	0.64	0.08	0.60	0.09	1.23	0.10	0.07		6.14	0.01	0.92	0.87
BM 1980,0611.68	blue-green	bust		1.94	19.5	650 - 700	14.2	0.48	3.07	71.3	0.15	0.76	0.81	8.60	0.07	0.02	0.42	3.75	77.4	9.60	8.96	1.22	4.59	9.62	10.3	3.81	2.89	10.8	402	6.50	42.7	1.37	0.39	0.29	0.04	0.00	0.61		0.10	248	6.10	10.9	1.42	6.08	1.23	0.35	1.15	0.17	1.08	0.21	0.61	0.08	0.59	0.08	1.08	0.08	0.05	0.02	4.16	0.01	0.81	0.92
BM 1980,0611.69	green	bust		1.47	17.9	650 - 700	14.1	0.73	3.01	65.6	0.12	0.84	0.69	9.42	0.09	0.20	0.61	4.54	84.3	11.9	12.4	13.7	43.3	27130	1297	4.00	53.1	8.97	489	6.92	44.9	1.59	0.80	41.7	0.11	6.19	1659	46.7	0.10	232	6.45	11.8	1.49	6.62	1.29	0.39	1.17	0.18	1.06	0.22	0.65	0.09	0.56	0.09	1.18	0.10	0.36	0.69	5585	3.37	0.86	0.74
BM 1986,0406.9	blue-green	cruciform monogram	Akakios/Kaios/ Akakos	2.19	20.0	6th - 7th	12.7	0.70	3.97	70.8	0.11	0.83	0.71	8.99	0.12	0.02	0.84	7.27	90.6	16.7	19.6	4.50	7.93	215	56.8	4.95	3.68	16.7	418	7.04	47.0	2.31	0.39	4.40	0.02	0.03	4.68		0.76	220	7.77	14.4	1.72	7.37	1.41	0.38	1.39	0.20	1.21	0.22	0.70	0.09	0.69	0.08	1.22	0.15	0.12	0.03	103	0.20	1.29	0.96
BM 1986,0406.10	blue-green	box monogram & inscription*	Ioannos*	1.45	18.6	1. half 7th	15.1	0.58	3.11	70.7	0.12	0.69	0.80	8.11	0.09	0.02	0.53	3.76	84.5	12.7	17.4	1.55	5.97	7.99	11.3	3.86	2.11	11.2	393	6.24	46.1	1.61	0.59	0.07	0.01	0.01	0.64	0.00	0.11	335	5.81	10.3	1.37	5.83	1.17	0.34	1.21	0.17	0.97	0.20	0.59	0.08	0.59	0.08	1.19	0.10	0.06		3.51		0.84	1.21
BM 1987,0703.3	blue-green	box monogram	Pakianos	2.11	20.6	6th - 7th	14.9	0.85	3.14	67.4	0.20	0.70	0.84	9.92	0.11	0.78	0.87	4.45	108	16.7	25.9	12.3	12.2	289	38.3	4.35	6.21	10.7	500	7.40	55.3	1.90	1.58	0.71	0.05	0.70	184	35.1	0.12	274	7.04	12.5	1.59	7.16	1.45	0.39	1.34	0.21	1.25	0.26	0.75	0.09	0.64	0.10	1.45	0.11	0.61	0.26	1332	0.19	1.02	0.89
BM 1987,0703.5	blue-green	box monogram	Pakianos	1.29	17.1	6th - 7th	14.6	0.85	3.15	67.4	0.18	0.74	0.78	10.4	0.10	0.64	0.82	4.89	105	15.5	39.7	11.1	13.0	321	31.9	4.36	4.97	9.93	532	7.31	50.4	1.78	1.40	0.88	0.07	0.92	235	26.4	0.11	258	6.67	11.9	1.57	6.81	1.42	0.40	1.38	0.21	1.23	0.25	0.68	0.09	0.61	0.09	1.25	0.11	0.81	0.22	1656	0.34	0.95	0.84
BM 1987,0703.15	blue-green	cruciform monogram	Aristomachos	4.32	25.2	6th - 7th	14.6	0.51	2.89	70.9	0.06	0.84	1.24	8.34	0.07	0.02	0.37	3.80	57.9	7.29	9.00	1.16	3.24	4.14	7.22	3.56	1.77	7.76	444	6.47	38.2	1.35	0.30	0.11	0.03	0.01	0.44		0.05	202	6.23	11.7	1.44	6.35	1.26	0.35	1.10	0.17	1.00	0.22	0.61	0.08	0.58	0.07	0.92	0.08	0.03				0.77	1.90
BM 1987,0703.17	blue-green	bust & inscription	Kosmas	1.35	17.5	608/609	13.5	0.75	3.26	70.9	0.07	0.91	0.56	9.28	0.09	0.02	0.52	3.19	90.3	10.1	6.06	1.62	4.53	10.8	8.68	4.03	1.83	8.22	504	7.53	42.5	1.69	0.37	0.13	0.02	0.01	0.68		0.06	222	6.94	12.7	1.63	6.97	1.39	0.38	1.27	0.19	1.15	0.24	0.66	0.09	0.63	0.09	1.04	0.09	0.05		0.55	0.01	0.88	0.72
BM S 322	blue-green	bust & inscription	Kosmas	4.41	25.1	608/609	14.2	0.81	3.27	68.2	0.11	0.84	0.49	11.1	0.10	0.03	0.64	4.13	116	12.6	15.4	2.67	7.18	76.7	16.2	4.09	2.46	6.98	531	7.92	42.8	1.81	0.47	2.91	0.08	0.06	10.7	0.19	0.06	220	7.45	13.6	1.68	7.38	1.47	0.40	1.32	0.21	1.25	0.26	0.71	0.09	0.68	0.10	1.08	0.10	0.05	1.20	47.3	0.03	0.93	0.80
BnF Froehner verre 09	green	bust & inscription	Ioannos	1.98	20.6	6th - 7th	14.1	0.77	3.27	67.6	0.12	0.88	0.52	12.0	0.08	0.02	0.50	4.28	83.2	9.10	19.3	3.22	5.65	10.6	8.46	69.6	2.24	7.27	580	8.07	40.8	1.57	0.40	0.16	0.05	0.01	1.95		0.05	238	7.21	13.4	1.67	6.96	1.43	0.43	1.29	0.22	1.23	0.26	0.73	0.09	0.67	0.10	1.02	0.09	0.06		21.7	0.02	0.90	0.73
BnF Froehner verre 11	aqua	bust & inscription	Rogatos	4.36	25.0	1.00	14.9	0.62	3.27	69.8	0.17	0.64	0.81	8.91	0.10	0.02	0.59	5.03	88.7	13.5	23.0	1.83	6.82	8.15	12.6	102	2.33	10.4	406	7.24	48.4	1.79	0.54	0.13	0.02	0.01	0.79		0.09	349	6.76	12.0	1.52	6.47	1.31	0.38	1.20	0.20	1.09	0.24	0.65	0.09	0.60	0.09	1.20	0.10	0.06	0.01	5.39		0.97	1.27
BnF inv 65.5420	aqua greenish	cruciform monogram		1.34	16.7	6th - 7th	15.2	0.71	3.14	69.3	0.15	0.83	0.97	9.07	0.07	0.02	0.39	3.36	111	7.81	13.6	1.29	3.66	6.54	7.43	63.6	2.14	11.9	446	6.99	39.2	1.38	0.60	0.24	0.01	0.00	0.46		0.10	211	6.13	11.4	1.45	6.13	1.27	0.40	1.22	0.19	1.11	0.24	0.64	0.09	0.58	0.09	1.00	0.08	0.05	0.06	13.4		0.79	0.53
BnF reg L 380	aqua greenish	box monogram & inscription*	Ioannos*	2.33	21.2	1. half 7th	16.0	0.60	2.98	71.3	0.06	0.81	0.68	6.94	0.08	0.02	0.41	3.11	94.4	9.28	14.3	1.28	3.84	4.74	10.9	68.1	0.37	9.83	397	6.24	45.4	1.48	0.45	4.45	0.01	0.00	0.74	1.06	0.07	234	5.88	10.8	1.37	5.63	1.13	0.33	1.00	0.17	0.97	0.21	0.57	0.08	0.53	0.08	1.17	0.09	0.05	0.03	5.34	3.18	0.83	1.07
BnF reg M 2542	aqua bluish	cruciform monogram & inscription	Sergios	1.42	17.9	610-641	15.6	0.71	3.02	68.6	0.10	1.01	0.53	9.56	0.09	0.02	0.54	4.56	91.7	10.6	22.3	2.99	5.98	7.71	10.6	68.7	2.31	7.52	468	7.21	45.1	1.67	0.26	0.14	0.03	0.01	1.53		0.07	241	6.70	12.5	1.58	6.51	1.35	0.40	1.18	0.20	1.19	0.25	0.66	0.09	0.63	0.09	1.12	0.10	0.04		17.8	0.04	0.85	1.04
BnF reg N 4263	greenish	cruciform monogram	Gregorios	2.24	19.9	6th - 7th	15.1	0.60	3.27	69.6	0.12	0.94	0.88	8.70	0.09	0.02	0.54	4.66	78.9	11.5	15.5	1.77	7.95	113	28.0	77.7	2.34	8.65	423	7.29	39.0	1.58	0.36	31.9	0.05	0.02	4.88		0.06	280	6.88	12.5	1.56	6.49	1.35	0.40	1.14	0.20	1.14	0.24	0.65	0.09	0.58	0.09	0.99	0.09	0.06	0.64	10.4	0.04	0.88	0.74
BnF Schlumberger 3976	colourless	box monogram & inscription*	Ioannos*	3.20	26.6	1. half 7th	14.7	0.55	2.72	73.4	0.06	0.76	0.58	6.68	0.07	0.01	0.38	3.55	84.2	8.98	12.8	1.19	3.66	3.43	9.50	3.60	1.35	8.86	387	6.23	44.7	1.45	0.47	0.04	0.02	0.00	0.42		0.07	230	6.26	11.4	1.41	5.75	1.12	0.34	0.94	0.17	0.98	0.21	0.57	0.07	0.51	0.08	1.11	0.08	0.05		5.10		0.88	1.06
BnF Schlumberger 3978	aqua greenish	bust & inscription	Droserios	4.43	25.7	ca. 600	13.3	0.66	3.07	71.8	0.07	0.93	0.57	8.95	0.07	0.02	0.43	3.39	83.3	8.20	12.7	2.25	4.36	7.97	7.90	4.01	2.71	9.52	494	7.31	39.0	1.50	0.50	0.06	0.01	0.02	2.51		0.06	219	6.98	12.7	1.58	6.56	1.33	0.40	1.14	0.19	1.16	0.24	0.66	0.09	0.59	0.08	0.99	0.08	0.08		16.4	0.01	0.86	0.66
BnF Schlumberger 3995	aqua bluish	bust & inscription	Neilos	4.25	25.1	6th - 7th	14.8	0.71	3.16	67.4	0.11	0.95	0.53	11.7	0.07	0.02	0.44	3.52	78.5	8.48	17.4	2.33	5.11	5.50	8.23	4.08	1.69	7.38	585	7.93	38.7	1.47	0.33	0.04	0.02	0.01	1.03		0.06	231	7.10	13.1	1.55	6.80	1.41	0.41	1.27	0.21	1.23	0.25	0.69	0.10	0.62	0.09	0.96	0.09	0.05		11.0		0.84	0.68
Levantine I cobalt blue																																																													
BM 1980,0611.12	cobalt blue	box monogram* & inscription	Anikios / Nikolaos / Kallinikos* & eparchos poleos	2.16	21.4	578-582	14.8	0.78	3.10	68.1	0.13	0.86	0.68	9.90	0.09	0.06	0.91	4.29	82.5	11.5	15.9	433	203	1433	27.6	5.19	9.94	8.68	480	6.88	39.5	1.51	2.54	0.13	0.01	0.48	35.4	3.00	0.09	230	6.08	11.4	1.47	6.58	1.29	0.39	1.29	0.19	1.16	0.22	0.65	0.08	0.63	0.09	1.01	0.09	0.09	0.01	2445	0.06	0.75	0.66
BM 1980,0611.13	cobalt blue	box monogram	Methodios / Theodomos	2.08	20.0	6th	15.9	0.76	3.48	63.7	0.10	0.69	0.69	12.0	0.09	0.17	1.16	3.43	92.8	13.1	19.8	784	266	2371	30.4	8.62	32.8	9.98	597	8.19	47.0	1.61	4.95	0.10	0.03	1.45	78.9	8.31	0.07	241	7.11	13.4	1.66	7.57	1.60	0.44	1.47	0.22	1.28	0.26	0.78	0.10	0.73	0.10	1.17	0.10	0.09	0.01	6246	0.07	0.91	1.01
BM 1980,0611.22	cobalt blue	cruciform monogram	Eupothios / Eupeithios	4.38	25.5	6th - 7th	14.7	0.86	3.46	66.1	0.11	0.93	0.62	10.3	0.09	0.16	1.57	4.14	85.7	14.2	22.1	1040	319	1963	52.8	9.76	17.5	7.83	527	7.46	42.9	1.63	6.72	0.28	0.05	1.40	87.8	8.37	0.08	243	6.70	12.2	1.56	6.96	1.39	0.41	1.44	0.22	1.32	0.26	0.70	0.10	0.70	0.09	1.13	0.09	0.07	0.01	4688	0.08	0.88	0.67
BM 1980,0611.27	cobalt blue	cruciform monogram	Theodoros / Dorotheos	4.49	24.7	6th - 7th	13.5	0.68	3.32	69.0	0.11	0.95	0.76	9.74	0.08	0.13	0.85	4.23	65.2	10.9	19.4	417	127	1788	25.3	5.06	9.35	11.1	492	7.08	37.1	1.43	2.57	2.43	0.07	1.07	218	3.46	0.10	266	6.27	11.7	1.52	6.65	1.29	0.41	1.38	0.19	1.14	0.24	0.67	0.08	0.59	0.08	0.96	0.09	2.21	0.33	3978	0.33	0.80	0.59
BM 1980,0611.29	cobalt blue	cruciform monogram	Eusippos / Eupothios / Eupeithios	4.41	25.1	6th - 7th	14.5	0.83	3.46	66.4	0.09	1.08	0.56	9.32	0.09	0.19	1.91	4.09	80.7	14.9	21.9	1551	483	2402	67.5	12.3	27.6	7.90	496	7.09	39.1	1.49	8.62	0.13	0.01	2.14	131	11.6	0.09	227	6.19	11.6	1.48	6.53	1.35	0.40	1.33	0.21	1.16	0.24	0.69	0.09	0.65	0.08	1.01	0.09	0.07		8190	0.10	0.79	0.60
BM 1980,0611.36	cobalt blue	cruciform monogram		2.16	20.0	6th - 7th	14.7	0.86	3.22	65.8	0.16	0.84	0.61	10.9	0.10	0.14	1.63	4.25	99.7	16.0	15.8	1027	309	2235	62.0	8.55	14.5	7.35	477	7.65	44.8	1.80	10.8	0.12	0.04	1.59	119	3.38	0.09	270	6.97	12.4	1.55	6.96	1.34	0.40	1.23	0.20	1.26	0.25	0.68	0.10	0.65	0.09	1.13	0.10	0.14		4475	0.07	0.95	0.69
BM 1980,0611.43	cobalt blue	cruciform monogram		2.24	19.2	6th - 7th	13.7	0.71	3.17	67.5	0.13	0.81	0.79	10.4	0.09	0.09	1.28	4.50	76.6	13.7	18.5	1267	412	2216	45.1	8.02	41.8	10.7	476	7.12	39.4	1.47	8.83	4.87	0.05	1.68	102	7.79	0.12	233	6.46	12.2	1.51	6.56	1.31	0.38	1.21	0.20	1.20	0.24	0.69	0.08	0.67	0.08	1.01	0.08	0.11	0.07	5831	0.13	0.84	0.72
BM 1980,0611.49	cobalt blue	bust & inscription		4.53	24.6	6th - 7th	13.9	0.64	3.09	69.0	0.13	0.80	0.79	9.40	0.08	0.08	1.03	3.65	70.2	12.0	13.6	838	286	2011	41.5	6.09	25.7	11.0	478	7.07	39.3	1.45	5.42	0.32	0.04	1.11	83.5	4.58	0.10	223	6.87	12.5	1.50	6.61	1.37	0.38	1.12	0.18	1.16	0.24	0.65	0.09	0.60	0.08	1.01	0.08	0.10	0.01	4858	0.08	0.82	0.72
BM 1980,0611.50	cobalt blue	bust & inscription		4.43	24.4	6th - 7th	14.8	0.77	3.13	67.0	0.10	1.00	0.57	9.65	0.09	0.16	1.53	3.65	85.7	14.0	13.9	1072	324	1976	53.0	10.1	20.1	8.23	528	7.31	43.3	1.66	7.72	0.20	0.01	1.53	94.0	8.82	0.07	232	7.26	13.2	1.59	6.98	1.38	0.37	1.12	0.20	1.08	0.23	0.66	0.09	0.60	0.08	1.01	0.10	0.07	0.03	5549	0.07	0.86	0.68
BM 1980,0611.53	cobalt blue	bust & inscription		2.25	20.6	6th - 7th	13.8	0.67	3.29	68.4	0.11	0.85	0.80	9.11	0.08	0.10	1.36	3.72	72.3	13.5	19.5	1321	439	2392	42.0	8.28	45.4	11.7	442	6.82	37.1	1.36	11.0	0.08	0.04	1.73	103	8.54	0.11	223	5.83	10.8	1.39	6.12	1.23	0.37	1.24	0.19	1.12	0.23	0.60	0.09	0.58	0.08	0.94	0.08	0.10		6832	0.14	0.72	0.64
BM 1980,0611.54	cobalt blue	bust & inscription	Theodosios	2.25	21.5	6th - 7th	13.4	0.66	3.11	68.6	0.12	0.81	0.75	9.78	0.08	0.09	1.23	4.40	71.9	13.7	16.1	1289	432	2393	41.7	7.78	50.0	10.8	473	7.20	40.8	1.49	9.81	0.21	0.07	1.68	97.5	8.98	0.10	230	6.82	12.3	1.53	6.77	1.32	0.37	1.22	0.20	1.20	0.22	0.66	0.08	0.60	0.08	1.04	0.09	0.11	0.02	6631	0.15	0.86	0.71
BM 1980,0611.56	cobalt blue	bust & inscription	Theodosios	1.54	18.6	6th - 7th	13.5	0.71	3.24	67.6	0.15	0.77	0.80	10.6	0.08	0.09	1.27	3.46	72.7	13.8	19.5	1265	414	2249	39.2	7.85	37.3	10.4	462	7.15	38.1	1.43	8.44	0.07	0.02	1.58	93.4	7.62	0.11	224	6.12	11.2	1.46	6.27	1.25	0.39	1.26	0.18	1.08	0.22	0.64	0.09	0.61	0.08	0.96	0.08	0.09		5232	0.09	0.76	0.67
BM 1980,0611.58	cobalt blue	bust & inscription		1.52	18.1	6th - 7th	14.2	0.62	3.29	69.0	0.10	0.88	0.84	8.42	0.08	0.11	1.35	3.10	67.9	13.2	13.9	861	277	2211	49.6	8.27	28.6	11.9	439	6.60	35.6	1.37	12.7	0.15	0.09	1.40	64.8	5.87	0.12	221	5.87	10.9	1.42	6.27	1.26	0.38	1.15	0.17	1.08	0.21	0.62	0.08	0.57	0.08	0.94	0.08	0.11	0.01	5891	0.26	0.73	0.68
BM 1980,0611.61	cobalt blue	bust with crown		4.31	23.6	650 - 700	14.5	0.82	3.09	66.7	0.14	0.83	0.91	9.11	0.10	0.44	1.40	5.66	98.6	17.3	16.1	1228	453	7186	258	7.76	27.7	10.3	484	6.91	47.9	1.70	4.62	2.70	0.02	1.52	189	33.4	0.11	241	6.45	11.6	1.50	6.56	1.37	0.36	1.27	0.19	1.15	0.23	0.63	0.09	0.65	0.09	1.20	0.11	0.12	0.09	5754	0.36	0.90	0.87
BM 1980,0611.62	cobalt blue	bust		4.37	25.6	650 - 700	14.0	0.73	3.36	67.3	0.17	0.84	0.86	10.9	0.09	0.28	0.90	4.62	78.6	15.7	13.7	522	110	1152	33.0	5.89	9.50	11.0	515	7.46	41.9	1.66	2.81	0.10	0.03	0.72	32.1	1.63	0.11	238	6.59	12.3	1.60	6.81	1.38	0.41	1.39	0.20	1.20	0.23	0.68	0.09	0.62	0.09	1.05	0.10	0.09		2643	0.09	0.87	0.92
BM 1980,0611.70	cobalt blue	bust with crown		1.45	16.8	650 - 700	13.9	0.67	3.14	68.2	0.11	0.82	0.81	9.58	0.09	0.17	1.16	4.11	80.2	16.4	8.06	468	153	1865	40.1	7.86	33.8	9.13	448	7.62	43.7	1.48	3.88	0.12	0.07	2.06	152	10.3	0.09	215	6.80	11.6	1.58	6.77	1.46	0.41	1.25	0.22	1.26	0.25	0.68	0.09	0.67	0.09	1.07	0.09	0.08		8135	0.11	0.84	0.76
BM 1987,0703.14	cobalt blue	cruciform monogram	Phokas	2.21	19.7	6th - 7th	12.9	0.68	3.41	68.8	0.16	0.87	0.98	9.85	0.10	0.10	1.19	4.73	75.0	14.2	14.4	654	167	2163	33.9	7.24	12.7	12.5	454	7.15	47.4	1.85	3.82	0.71	0.04	1.07	74.1	2.15	0.14	265	6.73	12.3	1.57	6.80	1.40	0.36	1.28	0.20	1.15	0.23	0.66	0.08	0.61	0.10	1.19	0.10	0.11	0.08	4316	0.11	0.95	0.65

S1 Table: LA-ICP-MS data of the Byzantine glass weights. Major and minor oxides [wt%], including chlorine, and trace elements [ppm]; where the monograms are ambiguous, multiple names are given (Tobias, forthcoming).

Sample Number	colour	type	names	weight [g]	Ø [mm]	Date	Na_2O	MgO	Al_2O_3	SiO_2	P_2O_5	Cl	K_2O	CaO	TiO_2	MnO	Fe_2O_3	Li	B	V	Cr	Co	Ni	Cu	Zn	Ga	As	Rb	Sr	Y	Zr	Nb	Mo	Ag	Cd	In	Sn	Sb	Cs	Ba	La	Ce	Pr	Nd	Sm	Eu	Gd	Tb	Dy	Ho	Er	Tm	Yb	Lu	Hf	Ta	W	Au	Pb	Bi	Th	U
**BM 1990,0601.14**	cobalt blue	cruciform monogram	Antiochos / Antilochos	4.40	25.4	6th - 7th	14.5	0.76	3.14	68.0	0.13	0.73	0.80	10.6	0.09	0.07	0.79	4.65	92.6	12.0	16.4	188	55.7	544	21.4	4.62	4.70	9.83	531	7.22	45.2	1.68	2.19	0.09	0.04	0.22	14.3	1.75	0.10	251	6.84	12.6	1.57	7.04	1.56	0.41	1.40	0.20	1.17	0.22	0.65	0.09	0.66	0.09	1.19	0.10	0.09	0.01	1139	0.03	0.93	0.95
**BnF Froehner verre 03**	cobalt blue	box monogram* & inscription	Anikios / Nikolaos / Kallinikos* & eparchos poleos	2.08	19.0	578-582	13.6	0.88	3.41	66.0	0.20	0.74	0.77	12.2	0.11	0.16	1.25	4.05	96.1	16.0	23.1	486	194	1299	32.3	85.4	7.22	9.67	523	8.31	50.5	2.05	4.76	0.14	0.07	0.82	92.7		0.08	277	7.41	13.3	1.68	7.19	1.48	0.43	1.36	0.22	1.33	0.27	0.74	0.11	0.65	0.10	1.20	0.12	0.31	0.01	2429	0.08	1.03	0.75
**BnF Froehner verre 36**	cobalt blue	box monogram	Methodios / Theodomos	3.10	22.6	6th	14.8	0.70	3.36	65.8	0.12	0.76	0.73	10.8	0.09	0.55	1.13	4.22	84.3	14.3	20.0	730	229	1737	33.4	76.6	30.3	10.7	518	8.27	50.5	1.81	6.54	0.75	0.06	1.31	76.3	8.64	0.11	260	7.72	13.9	1.71	7.29	1.47	0.43	1.27	0.21	1.30	0.27	0.71	0.10	0.67	0.10	1.23	0.10	0.15	0.15	5330	0.09	1.01	1.01
**BnF Schlumberger 3987**	cobalt blue	cruciform monogram	Megethios / Theognios	4.48	24.3	6th - 7th	13.7	0.74	3.36	67.3	0.16	0.79	0.86	10.7	0.09	0.32	1.22	3.84	76.4	17.0	26.9	775	159	1632	37.7	7.32	9.32	11.5	500	7.73	43.0	1.71	6.89	0.23	0.03	0.95	44.0	0.43	0.10	244	7.12	13.2	1.61	6.88	1.36	0.41	1.29	0.21	1.17	0.25	0.69	0.10	0.62	0.09	1.05	0.10	0.09	0.01	3091	0.15	0.93	0.93
**Foy-2**																																																														
**BM 1892,0613.59**	olive green	bust & inscription	Flavios Gerontios	4.27	25.0	560/562	16.9	1.06	2.59	67.6	0.15	0.78	0.81	7.39	0.15	1.41	0.95	6.57	171	33.6	14.3	8.84	16.0	32.0	23.2	4.14	4.75	8.90	619	7.72	90.0	2.55	3.71	0.14	0.01	0.02	3.32	55.5	0.13	381	7.68	12.4	1.76	7.39	1.51	0.39	1.42	0.20	1.28	0.25	0.72	0.10	0.76	0.11	2.09	0.14	0.24	0.01	27.5	0.02	1.24	0.99
**BM 1892,0613.60**	blue-green	bust & inscription	Flavios Gerontios	2.21	21.6	560/562	16.1	1.17	2.69	67.7	0.18	0.76	0.86	8.06	0.17	1.09	1.03	6.55	153	27.2	13.8	5.81	12.5	36.5	25.3	4.32	5.45	8.79	639	7.47	97.0	2.97	3.34	0.19	0.07	0.03	6.36	199	0.12	256	7.54	13.6	1.80	7.49	1.56	0.38	1.34	0.21	1.22	0.25	0.70	0.10	0.71	0.10	2.28	0.16	0.19	0.01	57.9	0.03	1.41	1.04
**BM 1892,0613.61**	clear / olive green	bust & inscription	Ioannos	4.43	25.0	538-541	17.6	1.15	2.86	65.3	0.15	0.85	0.84	8.24	0.14	1.54	1.04	6.58	149	28.8	11.8	10.7	21.5	135	36.8	4.33	6.16	15.2	681	7.41	77.7	2.49	4.84	2.10	0.12	0.06	5.66	52.2	0.25	259	7.33	12.4	1.69	7.34	1.47	0.35	1.35	0.22	1.26	0.24	0.73	0.09	0.72	0.10	1.89	0.14	0.24	0.26	128	0.09	1.34	1.02
**BM 1892,0613.62**	yellowish / olive green	box monogram	Pelagios / Paeitos	2.10	19.2	6th	16.7	1.36	2.62	64.0	0.18	0.76	0.73	10.1	0.17	1.75	1.23	7.36	162	41.1	14.2	12.5	11.9	160	38.3	4.59	5.68	6.33	777	8.04	93.4	2.86	2.02	0.64	0.05	0.19	44.1	224	0.08	412	8.19	14.1	1.86	8.02	1.65	0.38	1.54	0.22	1.43	0.27	0.80	0.10	0.78	0.11	2.25	0.18	0.14	0.04	1189	0.23	1.40	1.25
**BM 1892,0613.63**	clear with rose tinge	box monogram	Pelagios / Paeitos	1.35	18.6	6th	19.4	0.85	2.18	64.8	0.07	0.67	0.51	8.91	0.12	1.55	0.79	5.58	166	33.2	10.7	5.79	8.29	35.0	16.6	3.72	4.24	5.94	658	7.38	69.0	2.04	2.75	0.71	0.05	0.02	2.17	13.1	0.08	403	7.11	11.8	1.60	6.96	1.45	0.36	1.43	0.20	1.28	0.24	0.74	0.10	0.72	0.09	1.64	0.12	0.14	0.07	8.27	0.02	1.09	1.32
**BM 1892,0613.64**	rose	box monogram	Pelagios / Paeitos	1.07	16.6	6th	16.9	1.02	2.63	65.8	0.08	0.81	0.56	8.31	0.14	2.28	1.15	7.17	170	34.0	15.2	6.99	20.3	117	34.3	4.49	7.21	8.54	645	8.13	74.1	2.38	3.03	2.40	0.08	0.05	11.0	91.5	0.23	334	8.36	12.4	1.77	7.85	1.68	0.39	1.53	0.23	1.34	0.28	0.78	0.10	0.75	0.11	1.79	0.14	0.19	0.10	109	0.04	1.31	1.14
**BM 1893,0426.2**	green	box monogram	Symeonos	2.28	19.8	6th	17.2	0.96	2.27	66.3	0.09	0.79	0.62	9.28	0.14	1.34	0.81	6.06	169	23.3	6.55	5.03	7.46	30.5	16.9	4.03	4.08	7.05	665	7.47	80.3	2.38	1.91	0.11	0.06	0.04	8.73	136	0.11	224	7.23	12.5	1.63	7.15	1.44	0.33	1.32	0.21	1.25	0.25	0.71	0.10	0.67	0.10	1.88	0.13	0.11	0.01	55.9	0.03	1.23	1.22
**BM 1920,1104.1**	olive green	bust of emperor	Iustinianus	4.17	25.0	538-565	17.1	1.10	2.51	66.2	0.10	0.88	0.63	8.19	0.15	1.54	1.31	6.76	174	38.2	7.91	8.37	23.0	49.6	35.2	4.27	7.13	8.04	697	8.33	83.2	2.60	4.66	0.40	0.10	0.07	15.1	164	0.12	265	8.32	12.8	1.92	8.26	1.66	0.40	1.58	0.24	1.38	0.28	0.81	0.11	0.79	0.11	2.02	0.15	0.25	0.02	112	0.08	1.22	1.09
**BM 1980,0611.2**	blue-green	box monogram & bust	Martinos / Aristomachos	4.21	27.0	after 582	18.0	1.31	3.05	64.7	0.20	0.77	0.95	8.45	0.17	0.47	1.66	7.23	160	31.0	18.1	16.8	19.0	88.4	39.9	4.85	7.23	9.53	632	9.46	89.9	2.95	1.47	0.39	0.02	0.09	15.9	115	0.24	218	9.70	15.6	2.23	9.75	1.98	0.47	1.96	0.29	1.64	0.34	0.93	0.12	0.96	0.13	2.19	0.17	0.37	0.01	570	0.08	1.52	1.33
**BM 1980,0611.4**	greenish tinge	box monogram	Paulos	4.35	25.8	6th	16.9	0.96	2.22	67.7	0.19	0.76	0.88	8.01	0.12	1.36	0.72	6.46	120	21.8	12.0	6.25	8.60	77.3	23.1	3.68	3.46	8.16	604	6.45	76.2	2.00	2.30	0.47	0.04	0.13	30.0	59.3	0.08	253	6.24	10.6	1.40	6.31	1.29	0.32	1.24	0.18	1.12	0.22	0.64	0.08	0.61	0.09	1.84	0.12	0.16	0.03	115	0.03	1.07	0.98
**BM 1980,0611.5**	green	box monogram		3.08	23.3	650 - 700	19.0	0.70	2.13	67.3	0.06	0.94	0.50	6.91	0.11	1.49	0.66	5.03	179	23.4	11.9	4.55	8.27	29.8	15.6	3.73	4.59	6.09	524	6.18	62.7	1.81	2.51	0.11	0.04	0.03	6.23	8.05	0.08	271	6.05	10.1	1.40	6.02	1.23	0.33	1.10	0.18	1.05	0.21	0.59	0.09	0.59	0.08	1.51	0.10	0.16		25.6	0.03	0.92	1.16
**BM 1980,0611.6**	greenish tinge	box monogram		4.40	24.6	6th	18.0	1.18	2.59	64.9	0.15	0.75	0.74	8.66	0.15	1.67	0.93	6.49	141	28.3	17.4	7.27	14.0	51.2	27.5	4.25	4.69	7.48	734	7.47	88.9	2.59	4.96	0.50	0.12	0.04	7.47	115	0.12	301	7.56	12.7	1.68	7.40	1.45	0.38	1.45	0.22	1.33	0.25	0.73	0.10	0.71	0.10	2.12	0.14	0.30	0.08	90.6	0.03	1.35	0.97
**BM 1980,0611.8**	greenish tinge	box monogram	Addaeos / Aidesios	3.90	21.2	6th	18.1	0.79	2.23	66.7	0.05	0.87	0.77	7.84	0.12	1.63	0.75	5.41	179	24.3	5.74	4.80	7.99	45.3	18.3	3.83	4.09	6.41	538	7.18	71.7	2.12	2.42	3.98	0.10	0.03	4.77	78.4	0.09	267	7.29	12.1	1.59	6.86	1.43	0.34	1.20	0.19	1.21	0.23	0.65	0.09	0.63	0.10	1.73	0.12	0.14	0.06	29.9	0.08	1.17	1.32
**BM 1980,0611.19**	greenish tinge	box monogram	Addaeos / Aidesios	1.22	17.4	6th	17.1	1.02	2.37	66.0	0.11	0.83	0.61	8.67	0.13	1.75	1.16	6.05	161	30.4	15.3	6.05	12.0	43.3	23.1	4.13	7.38	6.71	689	8.22	79.1	2.39	2.33	0.11	0.04	0.03	3.28	290	0.10	305	8.62	12.8	1.86	7.92	1.65	0.40	1.55	0.24	1.41	0.30	0.80	0.11	0.81	0.11	1.93	0.14	0.19	0.01	43.1	0.02	1.30	1.32
**BM 1980,0611.23**	olive green	cruciform monogram	Nikolaos / Nikaios / Anikios	3.43	26.1	6th - 7th	17.1	1.49	2.55	64.5	0.23	0.75	0.95	8.48	0.24	1.75	1.65	6.38	177	46.5	20.8	13.0	19.7	68.1	48.4	4.76	5.77	6.57	686	8.57	123.0	3.29	2.67	0.25	0.06	0.04	6.67	23.5	0.12	508	8.80	13.7	1.98	8.49	1.71	0.44	1.64	0.26	1.52	0.32	0.90	0.12	0.92	0.14	2.93	0.20	0.50	0.01	93.5	0.03	1.60	1.37
**BM 1980,0611.24**	purple	cruciform monogram		3.29	22.3	6th - 7th	16.9	1.10	2.49	63.4	0.12	0.72	0.68	10.2	0.15	2.61	1.27	8.80	162	37.0	11.4	9.86	14.4	64.5	32.4	4.75	7.98	6.55	892	8.85	87.0	2.68	3.84	0.54	0.12	0.05	9.07	216	0.09	391	9.06	13.7	1.96	8.62	1.70	0.44	1.64	0.24	1.49	0.31	0.87	0.12	0.86	0.12	2.13	0.15	0.25	0.04	81.5	0.03	1.33	1.36
**BM 1980,0611.44**	blue-green	cruciform monogram	Dometios / Modestos	1.29	18.0	6th - 7th	16.6	0.99	2.78	67.3	0.15	0.76	0.88	8.66	0.12	0.70	0.93	6.31	128	21.6	19.9	9.69	12.2	161	28.5	4.19	4.36	9.77	551	6.99	63.8	2.07	1.82	0.28	0.07	0.06	12.7	40.1	0.12	262	6.89	11.9	1.62	6.99	1.44	0.42	1.37	0.20	1.20	0.24	0.68	0.09	0.68	0.10	1.61	0.12	0.17	0.05	141	0.03	1.05	0.99
**BM 1980,0611.46**	greenish tinge	cruciform monogram	Eusebios / Bessos	1.19	17.4	6th - 7th	20.1	1.19	2.42	63.4	0.21	0.85	0.88	7.75	0.13	0.98	1.89	5.61	143	40.4	16.8	13.9	23.9	52.4	33.1	4.30	9.47	7.42	619	9.02	78.6	2.24	2.66	0.14	0.05	0.03	3.23	25.2	0.16	242	9.14	11.7	2.14	9.39	1.89	0.48	2.11	0.29	1.65	0.33	0.92	0.12	0.89	0.13	1.91	0.13	0.30	0.01	34.9	0.03	1.15	1.32
**BM 1980,0611.64**	translucent blue	bust with crown		4.17	23.1	650 - 700	16.7	1.13	2.57	66.5	0.18	0.80	0.97	7.90	0.14	1.53	1.03	7.33	150	30.5	11.0	64.6	36.7	1888	31.7	4.88	8.55	9.14	648	7.19	82.7	2.45	4.92	0.15	0.08	0.16	8.73	101	0.13	324	7.45	12.7	1.74	7.18	1.38	0.36	1.35	0.20	1.22	0.26	0.68	0.10	0.69	0.09	2.10	0.14	0.26	0.01	1220	0.03	1.24	1.01
**BM 1980,0611.67**	greenish tinge	bust		2.06	18.5	650 - 700	17.3	1.05	2.77	66.3	0.17	0.80	0.76	7.48	0.15	1.46	1.48	8.49	143	43.3	14.0	11.4	17.4	114	29.7	4.48	7.24	8.30	607	8.09	81.9	2.35	3.34	0.19	0.04	0.03	6.04	53.7	0.12	303	7.89	11.9	1.78	7.98	1.63	0.43	1.54	0.23	1.31	0.29	0.77	0.11	0.84	0.11	1.96	0.14	0.24	0.01	121	0.03	1.12	0.96
**BM 1980,0611.90**	blue-green	bust & inscription	Mousilios	3.56	26.0	615-641	16.3	0.97	2.60	68.3	0.15	0.69	0.77	8.23	0.15	0.66	0.95	7.52	123	23.5	16.5	8.99	11.4	103	34.3	3.93	6.17	9.01	533	7.33	72.3	2.37	1.81	0.74	0.06	0.06	14.0	46.6	0.14	252	7.70	13.2	1.74	7.48	1.45	0.38	1.36	0.19	1.19	0.24	0.67	0.09	0.64	0.10	1.67	0.14	0.17	0.14	173	0.05	1.14	1.02
**BM 1981,0601.1**	green	box monogram & letters	Nikolaos	1.43	21.0	6th - 7th	16.6	1.00	2.63	66.1	0.19	0.60	0.72	9.54	0.17	1.07	1.07	11.4	144	25.7		9.93	10.1	118	50.8	4.34	7.53	6.30	718	7.59	92.4	2.69	1.90	0.46		0.28	66.2	129	0.10	264	7.57	12.5	1.74	7.44	1.56	0.36	1.28	0.22	1.27	0.25	0.75	0.09	0.76	0.10	2.33	0.18	0.18	0.45	536	0.23	1.45	1.23
**BM 1981,0601.2**	clear / greenish tinge	cruciform monogram		1.41	18.8	6th - 7th	16.3	1.31	2.73	66.3	0.22	0.76	1.03	7.96	0.16	1.69	1.26	9.31	150	36.2	8.64	11.3	21.5	91.2	36.7	4.64	5.66	9.43	700	8.23	89.0	2.77	5.20	0.37	0.12	0.04	6.13	129	0.16	294	8.80	14.1	1.90	8.18	1.56	0.40	1.41	0.22	1.41	0.28	0.80	0.11	0.77	0.10	2.15	0.15	0.24	0.04	105	0.05	1.42	1.03
**BM 1981,0601.3**	clear	box monogram	Dikoros / Dioskoros	0.93	16.2	6th	19.5	0.58	2.05	67.3	0.05	0.93	0.45	6.94	0.09	1.29	0.64	4.06	156	26.2	12.0	5.37	8.66	28.7	16.6	3.44	3.44	5.64	482	6.42	54.2	1.58	2.43	0.05	0.03	0.01	3.44		0.06	260	6.27	9.61	1.39	6.09	1.29	0.31	1.22	0.17	1.17	0.24	0.63	0.08	0.60	0.09	1.39	0.10	0.33		12.9		0.89	1.07
**BM 1981,0601.4**	greenish tinge	box monogram	Mounios / Mousilios	2.01	19.3	6th	17.7	1.08	2.47	66.3	0.15	0.78	0.70	8.03	0.14	1.57	0.88	5.70	139	26.6	16.1	4.98	14.7	39.0	25.3	4.20	4.05	7.89	660	7.03	82.7	2.48	4.96	0.26	0.16	0.02	4.72	39.9	0.10	234	7.08	12.2	1.61	7.01	1.43	0.34	1.32	0.20	1.23	0.23	0.66	0.09	0.70	0.09	2.00	0.14	0.20	0.01	31.7	0.02	1.24	1.03
**BM 1981,0601.5**	clear / greenish tinge	cruciform monogram	Addaeos	1.15	16.1	551/565	16.8	1.22	2.83	66.2	0.18	0.75	0.86	7.79	0.16	1.77	1.19	7.19	156	34.5	13.7	11.6	20.4	68.3	32.2	4.52	5.75	8.47	732	7.93	86.0	2.69	5.45	0.13	0.12	0.03	4.81	69.3	0.14	271	8.26	13.2	1.79	7.98	1.67	0.40	1.50	0.23	1.32	0.26	0.78	0.10	0.79	0.11	2.15	0.15	0.23	0.05	78.0	0.03	1.32	1.10
**BM 1981,0601.7**	cobalt blue	bust & inscription	Demosthenos	1.35	18.2	6th - 7th	18.7	0.75	1.88	66.2	0.07	0.90	0.43	7.07	0.10	2.53	1.15	5.12	181	31.0	12.1	6.85	12.7	46.5	20.1	3.91	9.14	5.68	588	7.61	59.2	1.63	3.75	0.14		0.02	1.31	20.2	0.07	343	7.55	10.2	1.68	7.45	1.45	0.37	1.48	0.21	1.39	0.27	0.71	0.10	0.80	0.10	1.45	0.08	0.23		14.2	0.02	0.82	1.13
**BM 1983,1108.2**	blue-green	bust & inscription	Tiberios	4.46	26.6	6th - 7th	15.3	0.90	2.60	69.4	0.15	0.73	1.00	7.80	0.14	0.75	0.97	5.61	128	24.2	16.1	8.59	16.8	304	56.4	4.02	4.78	10.2	557	7.02	84.6	2.41	1.80	2.62	0.11	0.05	12.2	65.2	0.11	265	7.46	12.6	1.65	6.99	1.42	0.37	1.22	0.20	1.21	0.24	0.69	0.10	0.66	0.09	1.97	0.15	0.20	0.06	204	0.27	1.26	1.11
**BM 1984,0108.2b**	blue-green	2 busts & cruciform monogram	Genethlios	6.66	29.8	615-641	16.2	1.10	2.79	67.4	0.16	0.79	0.86	8.86	0.13	0.62	0.99	5.65	130	22.0	17.3	9.75	12.3	67.9	34.6	3.97	4.35	9.28	565	7.48	64.5	2.15	1.66	0.42	1.22	0.04	8.58	43.2	0.10	251	7.00	11.9	1.66	7.24	1.53	0.39	1.41	0.22	1.25	0.25	0.69	0.10	0.68	0.10	1.56	0.13	0.14	0.03	96.0	0.03	1.06	0.99
**BM 1984,0109.1**	blue-green	cruciform monogram	Aristomachos	4.48	26.7	6th - 7th	16.3	1.06	2.64	67.4	0.17	0.68	1.06	8.51	0.15	0.77	1.04	8.59	135	24.5	17.0	10.4	13.3	73.3	31.2	4.22	4.79	9.52	561	7.28	73.6	2.36	2.01	0.22	0.10	0.06	10.9	51.0	0.14	272	7.41	12.7	1.72	7.42	1.53	0.35	1.38	0.22	1.30	0.25	0.67	0.10	0.70	0.10	1.80	0.13	0.22	0.08	123	0.04	1.16	1.04
**BM 1986,0406.6**	green	cruciform monogram	Ioulianos	1.10	18.3	580	17.2	1.17	2.65	64.9	0.18	0.78	0.74	8.74	0.15	1.65	1.59	8.47	163	43.8	17.5	12.8	22.7	71.6	36.5	4.84	7.42	7.61	769	8.38	84.3	2.62	4.65	0.21	0.12	0.04	5.41	66.9	0.13	269	8.43	12.9	1.90	8.39	1.79	0.43	1.71	0.24	1.51	0.29	0.81	0.11	0.81	0.12	2.14	0.15	0.19	0.04	80.0	0.03	1.29	1.16
**BM 1986,0406.7**	greenish tinge	cruciform monogram	Nikolaos / Nikaios / Anikios	3.88	25.8	6th - 7th	16.8	1.15	2.66	66.6	0.09	0.84	0.64	8.35	0.14	1.49	0.98	7.02	154	27.5	14.5	6.30	15.9	43.9	24.4	4.51	5.93	8.11	671	7.57	74.1	2.43	3.98	0.31	0.06	0.03	4.79	462	0.15	336	7.88	12.9	1.78	8.00	1.50	0.41	1.58	0.22	1.34	0.25	0.75	0.10	0.75	0.11	1.85	0.14	0.37	0.03	66.7	0.04	1.26	1.06
**BM 1986,0406.8**	colourless	cruciform monogram	Megethios	0.94	18.3	6th - 7th	19.6	1.25	2.57	64.6	0.20	0.84	0.93	7.10	0.14	1.32	1.28	6.16	155	34.1	10.1	11.9	18.7	59.8	30.1	4.41	5.48	7.53	579	7.88	77.6	2.40	2.76	0.11	0.05	0.03	4.92	115	0.14	308	8.44	12.5	1.86	8.15	1.78	0.41	1.56	0.23	1.39	0.28	0.81	0.10	0.78	0.11	1.85	0.13	0.30	0.01	61.6	0.03	1.24	1.13
**BM 1986,0406.15**	blue-green	bust & inscription	Theodoros	1.19	17.5	6th - 7th	16.3	1.02	2.59	67.8	0.18	0.83	0.84	8.76	0.12	0.52	0.86	4.75	129	20.0	14.6	11.0	11.3	56.0	23.3	3.81	5.74	8.07	562	7.14	67.8	2.06	1.65	0.57	0.02	0.06	8.92	38.8	0.09	238	7.42	12.7	1.61	7.14	1.38	0.36	1.24	0.17	1.22	0.25	0.71	0.10	0.66	0.10	1.62	0.12	0.15	0.04	93.8	0.02	1.14	0.99
**BM 1986,0406.16**	colourless	bust & inscription		1.14	17.2	6th - 7th	17.2	0.89	2.14	69.1	0.04	0.86	0.45	6.53	0.11	1.78	0.69	4.15	173	25.8	9.76	4.17	15.3	24.9	24.9	3.87	4.50	6.12	578	6.16	60.2	1.81	5.58	0.06	0.11	0.00	0.89		0.07	241	6.45	10.3	1.45	6.28	1.32	0.31	1.17	0.18	1.06	0.21	0.59	0.08	0.58	0.07	1.52	0.11	0.12	0.01	6.03		0.93	0.91
**BM 1986,0406.17**	blue-green	bust & inscription	Symeonos	1.93	21.3	6th - 7th	16.3	1.28	2.48	67.0	0.22	0.72	1.00	9.29	0.13	0.41	0.92	8.58	140	20.0	15.6	9.09	10.7	48.0	22.9	3.77	6.12	8.80	582	6.85	71.5	2.18	1.38	0.50	0.04	0.04	9.01	33.8	0.11	244	7.14	12.5	1.60	7.02	1.39	0.35	1.24	0.19	1.22	0.25	0.67	0.09	0.66	0.09	1.83	0.13	0.13	0.06	68.5	0.02	1.14	1.10
**BM 1986,0406.18**	amber	bust, inscription* & cruciform monogram**	Iustinianus* & Sergios**	4.24	24.7	538-565	19.7	0.97	2.38	63.8	0.09	0.96	0.55	8.09	0.17	1.63	1.40	5.53	129	38.2	10.5	8.30	20.3	51.3	27.7	3.96	8.10	6.27	694	8.59	91.9	2.52	5.36	0.13	0.09	0.03	2.89	86.2	0.08	229	8.52	12.0	1.90	8.47	1.76	0.43	1.64	0.25	1.48	0.31	0.85	0.11	0.83	0.11	2.22	0.14	0.18	0.01	52.4	0.02	1.21	1.08
**BM 1986,0406.21**	blue-green	cross & letter	Konos / Sitonikos	1.24	16.5	6th - 7th	15.7	1.00	2.61	64.5	0.17	0.66	0.69	12.6	0.12	0.26	0.90	5.67	118	16.2	16.3	23.5	10.4	1463	66.3	3.99	11.2	8.03	575	6.60	58.6	2.38	0.97	1.25	0.04	0.76	193	1944	0.14	214	7.04	12.7	1.59	6.83	1.37	0.37	1.29	0.21	1.08	0.23	0.64	0.09	0.63	0.09	1.41	0.13	0.25	1.71	2225	0.21	1.18	1.23
**BM 1987,0703.2**	olive green translucent	box monogram	Andronikos	2.09	19.4	6th	15.9	1.22	2.76	65.6	0.11	0.80	0.72	10.1	0.17	1.31	1.05	7.47	168	26.5	19.5	8.09	10.0	44.8	23.0	4.80	5.46	8.52	748	8.18	98.8	3.02	1.93	0.41	0.13	0.05	9.16	360	0.14	253	8.46	15.0	1.91	8.46	1.66	0.39	1.62	0.22	1.37	0.28	0.80	0.11	0.81	0.12	2.36	0.17	0.17	0.07	57.5	0.02	1.49	1.22
**BM 1987,0703.4**	mauve	box monogram	Thomas	3.78	22.9	6th	16.8	0.60	2.10	70.1	0.04	0.87	0.43	6.05	0.10	2.21	0.55	4.15	152	23.5	15.8	4.33	6.40	21.1	12.2	3.76	2.80	5.81	500	5.40	60.8	1.70	3.12	0.07	0.06	0.01	0.66	3.05	0.05	266	5.43	9.19	1.23	5.41	1.15	0.28	1.10	0.15	0.92	0.19	0.53	0.08	0.53	0.07	1.51	0.10	0.13		4.99	0.01	0.88	1.18
**BM 1987,0703.6**	amber	box monogram	Paulos	1.85	18.8	6th	15.9	0.96	2.47	66.8	0.09	0.80	0.58	9.45	0.15	1.75	0.88	6.39	118	27.5	14.0	5.59	9.24	30.1	17.6	4.34	4.45	7.65	707	7.76	89.4	2.61	1.87	0.13	0.04	0.03	4.18	210	0.13	268	7.62	13.0	1.74	7.49	1.66	0.36	1.51	0.21	1.28	0.26	0.77	0.10	0.69	0.10	2.21	0.15	0.15	0.01	36.7	0.02	1.34	1.12
**BM 1987,0703.11**	colourless	cruciform monogram	Ioulianos	0.97	18.3	580	17.1	1.23	2.67	66.2	0.18	0.84	0.92	7.57	0.15	1.96	1.00	7.50	174	32.1	17.7	10.3	19.6	66.6	34.4	4.64	4.52	8.08	687	7.01	85.3	2.50	5.89	0.13	0.08	0.03	3.71	16.3	0.12	298	7.29	12.0	1.67	7.29	1.47	0.36	1.46	0.20	1.22	0.25	0.73	0.10	0.69	0.10	2.17	0.14	0.19	0.01	63.5	0.02	1.28	1.06
**BM 1987,0703.12**	colourless	cruciform monogram		1.15	18.3	6th - 7th	17.2	1.20	2.58	66.2	0.16	0.81	0.79	8.06	0.16	1.58	1.01	7.09	190	31.5	11.2	8.13	16.9	55.9	29.0	4.56	4.93	7.46	694	7.25	90.3	2.72	4.73	0.11	0.07	0.03	4.01	97.9	0.12	255	7.58	12.8	1.70	7.34	1.51	0.36	1.39	0.21	1.21	0.25	0.72	0.10	0.74	0.10	2.20	0.15	0.24	0.01	42.9	0.02	1.35	1.32

S1 Table: LA-ICP-MS data of the Byzantine glass weights. Major and minor oxides [wt%], including chlorine, and trace elements [ppm]; where the monograms are ambiguous, multiple names are given (Tobias, forthcoming).

Sample Number	colour	type	names	weight [g]	Ø [mm]	Date	Na_2O	MgO	Al_2O_3	SiO_2	P_2O_5	Cl	K_2O	CaO	TiO_2	MnO	Fe_2O_3	Li	B	V	Cr	Co	Ni	Cu	Zn	Ga	As	Rb	Sr	Y	Zr	Nb	Mo	Ag	Cd	In	Sn	Sb	Cs	Ba	La	Ce	Pr	Nd	Sm	Eu	Gd	Tb	Dy	Ho	Er	Tm	Yb	Lu	Hf	Ta	W	Au	Pb	Bi	Th	U
**BM 1987,0703.16**	colourless greenish	cruciform monogram	Prokopios	2.00	20.5	6th - 7th	17.0	1.24	2.71	66.6	0.20	0.77	0.94	7.50	0.15	1.71	1.02	6.81	159	34.3	9.46	10.9	18.4	60.5	27.3	4.51	4.59	8.31	645	7.24	83.9	2.52	4.49	0.35	0.05	0.03	4.25	34.8	0.13	397	7.55	12.3	1.68	7.19	1.43	0.39	1.37	0.21	1.24	0.26	0.68	0.10	0.72	0.10	2.03	0.14	0.26	0.01	46.5	0.03	1.27	1.01
**BM 1987,0703.18**	green	bust & inscription	Flavios Zimarchos	1.64	18.9	562/565	16.3	1.19	2.74	66.1	0.26	0.72	1.08	7.74	0.16	1.45	2.01	7.45	140	54.4	15.1	14.7	23.6	81.0	39.0	4.51	9.13	9.67	635	8.81	77.8	2.56	3.53	0.13	0.09	0.05	6.47	47.6	0.15	334	9.20	12.8	2.07	8.80	1.84	0.46	1.62	0.25	1.46	0.30	0.86	0.12	0.87	0.11	1.77	0.14	0.25	0.01	118	0.03	1.22	1.06
**BM 1987,0703.19**	green	bust & inscription	Ioannos	3.63	24.8	538-541	19.2	1.14	2.46	64.4	0.15	0.89	0.72	8.52	0.15	1.23	0.86	6.08	147	25.9	8.28	5.21	13.9	36.3	23.7	3.91	4.62	7.57	675	7.44	86.8	2.70	4.02	0.12	0.10	0.04	5.88	148	0.10	214	7.28	12.7	1.66	7.11	1.42	0.34	1.22	0.20	1.23	0.26	0.71	0.10	0.70	0.10	2.06	0.15	0.16		43.1	0.02	1.31	1.00
**BM 1990,0601.13**	greenish tinge	box monogram	Sisinniolos	1.37		6th	16.9	0.74	2.25	66.9	0.07	0.95	0.62	8.78	0.11	1.57	0.85	7.16	147	34.0	13.5	7.46	11.6	25.1	18.9	3.77	4.10	7.00	636	7.07	66.3	2.03	2.01	0.09	0.06	0.02	2.34	0.26	0.10	331	6.83	11.0	1.52	6.73	1.43	0.35	1.29	0.19	1.18	0.25	0.69	0.10	0.68	0.10	1.62	0.11	0.32		12.0	0.01	1.09	1.26
**BM 1990,0601.17**	dark red	3 busts & cruciform monogram	Euthalios / Autheios	2.21	20.8	6th - 7th	16.9	1.10	2.68	63.9	0.14	0.76	0.76	8.79	0.15	1.46	2.56	8.91	155	32.6	17.3	798	74.7	1894	138	5.54	10.1	9.08	680	7.47	77.4	2.53	12.70	0.80	0.12	0.90	87.6	131	0.16	318	7.13	12.6	1.69	7.15	1.54	0.38	1.48	0.22	1.28	0.26	0.74	0.10	0.74	0.10	1.93	0.15	0.19	0.01	1940	0.16	1.28	1.11
**BM 1990,0601.18**	green	2 busts & box monogram	Paulos	2.04	21.4	565-578	16.5	1.14	2.51	66.6	0.12	0.75	0.67	8.74	0.15	1.57	1.01	10.6	180	32.2	2.73	8.18	15.1	62.5	24.3	4.31	6.17	8.13	686	7.77	84.4	2.64	4.03	0.15	0.04	0.05	11.5	254	0.15	337	8.06	13.7	1.79	7.62	1.57	0.39	1.28	0.21	1.31	0.27	0.72	0.10	0.72	0.10	2.04	0.15	0.25	0.01	86.1	0.03	1.31	1.19
**BM 1997,0218.1**	yellowish / olive green	bust, inscription* & cruciform monogram**	Iustinianus* & Sergios**	4.46	27.0	538-565	19.5	1.06	2.36	64.3	0.15	0.99	0.75	8.10	0.13	1.55	0.90	6.89	142	29.5	7.04	7.10	18.5	40.6	23.8	3.98	5.14	9.66	689	7.15	74.4	2.33	4.96	0.31	0.08	0.04	7.26	92.7	0.14	293	7.15	11.8	1.67	7.03	1.42	0.36	1.30	0.20	1.22	0.24	0.65	0.10	0.66	0.09	1.77	0.14	0.31	0.01	107	0.03	1.15	0.93
**BnF AA de Clercq 5**	opaque dark red	cruciform monogram	Heraklion	2.00	19.1	6th - 7th	16.8	0.78	2.57	68.3	0.05	0.99	0.40	8.22	0.13	0.89	0.73	4.11	161	26.1	18.4	5.94	8.84	14.5	16.4	4.30	1.66	6.33	493	7.80	85.1	2.49	1.11	0.06	0.03	0.01	1.02		0.08	334	7.82	13.3	1.65	6.94	1.42	0.36	1.31	0.21	1.28	0.26	0.71	0.10	0.72	0.11	1.98	0.14	0.38		7.15		1.35	1.43
**BnF AA de Clercq 6**	yellowish green	cruciform monogram	Megethios	4.25	25.9	6th - 7th	14.2	1.15	2.44	61.7	0.27	0.70	0.88	8.33	0.15	1.33	1.69	4.80	132	28.4	18.7	73.0	183	10325	5454	5.12	117	7.14	737	7.58	78.3	2.58	4.42	3.29	0.15	67.15	16866	274	0.11	247	7.62	12.8	1.64	6.85	1.37	0.36	1.31	0.20	1.25	0.25	0.70	0.10	0.69	0.11	1.87	0.16	0.57	0.02	26131	3.02	1.26	0.96
**BnF AA VA 16**	aqua bluish	cruciform monogram & inscription	Theodoros	1.47	19.1	6th - 7th	16.0	1.08	2.75	66.7	0.24	0.66	1.11	9.42	0.13	0.68	0.96	7.70	127	22.9	20.0	9.98	13.3	73.7	28.8	86.5	4.98	13.3	594	7.53	68.5	2.27	1.82	0.19	0.09	0.06	12.0	46.5	0.13	320	8.06	13.6	1.80	7.26	1.45	0.40	1.23	0.21	1.21	0.27	0.71	0.10	0.67	0.10	1.65	0.13	0.20	0.04	213	0.05	1.18	1.03
**BnF AA VA 18**	yellowish green	box monogram	Elia	3.94	24.0	541.00	16.1	0.97	2.56	67.2	0.08	0.81	0.60	9.05	0.17	1.27	0.91	6.08	135	26.8	21.1	4.83	8.10	25.6	18.5	64.2	5.14	8.02	689	8.28	101.7	3.06	1.40	0.09	0.05	0.02	4.27	433	0.12	234	8.66	14.7	1.88	7.81	1.54	0.39	1.31	0.23	1.34	0.28	0.77	0.11	0.75	0.11	2.51	0.18	0.13	0.01	58.7	0.02	1.48	1.21
**BnF AA VA 21**	green	cruciform monogram	Ioulianos	4.27	25.3	580	16.5	1.10	2.75	67.4	0.18	0.69	1.02	7.76	0.15	0.99	1.21	6.71	145	33.2	19.5	12.5	16.8	81.0	34.6	77.5	5.65	8.65	649	8.32	88.6	2.66	2.49	0.25	0.04	0.04	5.56	42.2	0.12	277	8.58	13.5	1.90	7.72	1.46	0.39	1.36	0.24	1.40	0.30	0.83	0.11	0.78	0.12	2.14	0.16	0.15	0.01	131	0.02	1.38	1.08
**BnF Froehner verre 01**	green	bust & inscription	Iustinus	2.03	19.9	565-578	15.8	1.31	2.87	66.5	0.23	0.69	0.91	9.11	0.18	0.94	1.31	6.64	170	30.0	21.6	16.0	20.5	57.3	36.9	67.3	4.87	9.34	740	8.35	96.7	3.16	3.22	0.13	0.09	0.03	5.64	53.4	0.17	223	8.72	14.5	1.98	8.06	1.64	0.45	1.49	0.24	1.40	0.30	0.82	0.12	0.78	0.12	2.31	0.18	0.19		66.6	0.02	1.48	1.13
**BnF Froehner verre 02**	amber	inscription	Anastasios	2.27	18.7	6th - 7th	18.6	0.86	2.05	65.5	0.09	1.01	0.68	8.63	0.11	1.50	0.74	4.28	133	23.9	15.7	5.29	10.8	74.7	17.9	101	4.36	6.25	709	7.34	62.0	2.01	3.38	0.09	0.08	0.02	4.69	149	0.08	337	7.00	11.4	1.60	6.66	1.31	0.33	1.26	0.20	1.21	0.25	0.65	0.09	0.69	0.10	1.49	0.12	0.17		46.6		1.02	0.91
**BnF Froehner verre 04**	green	bust & inscription	Zimarchos	2.18	19.0	562/565	17.0	1.26	2.77	65.8	0.17	0.76	0.81	8.27	0.17	1.71	1.00	5.66	166	31.4	20.6	6.41	15.1	63.0	29.3	75.3	2.84	8.81	724	7.69	95.4	3.03	5.36	3.17	0.16	0.03	6.30	110	0.12	249	7.90	13.4	1.79	7.48	1.59	0.37	1.35	0.22	1.28	0.26	0.75	0.11	0.70	0.11	2.31	0.18	0.20	0.06	59.9		1.42	1.13
**BnF Froehner verre 08**	greenish	bust & inscription	Ioannos	2.25	21.0	538-541	18.6	1.24	2.66	64.5	0.12	1.02	0.67	8.46	0.14	1.20	1.08	6.73	176	33.8	17.1	9.92	22.7	42.1	26.1	89.1	6.81	8.74	768	8.47	84.4	2.70	5.29	0.54	0.07	0.05	7.76	180	0.17	302	8.95	14.0	1.94	8.05	1.68	0.41	1.42	0.24	1.38	0.30	0.80	0.12	0.77	0.11	1.97	0.15	0.35	0.09	73.5	0.05	1.35	1.06
**BnF Froehner verre 12**	aqua	bust & inscription	Rogatos	1.95	21.0	610/612	16.4	1.31	2.61	66.8	0.24	0.72	1.00	9.26	0.13	0.39	0.95	6.52	143	20.3	21.4	9.80	11.2	46.3	21.6	70.7	3.73	8.16	581	6.99	68.2	2.30	1.30	1.04	0.04	0.04	6.96	32.3	0.09	243	7.16	12.5	1.60	6.67	1.32	0.36	1.19	0.20	1.14	0.23	0.66	0.09	0.63	0.09	1.65	0.13	0.13	0.52	77.2	0.02	1.12	1.09
**BnF Froehner verre 21**	dark purple	bust & inscription	Theodoros	3.62	22.5	6th - 7th	17.6	1.01	2.46	66.2	0.10	0.93	0.53	8.18	0.13	1.78	0.84	5.69	166	28.7	17.4	11.0	10.4	63.2	22.8	93.6	4.91	6.57	656	7.59	73.0	2.21	2.92	0.10	0.06	0.04	6.90	109	0.10	319	7.50	12.4	1.64	6.78	1.40	0.35	1.17	0.20	1.26	0.24	0.70	0.10	0.74	0.10	1.78	0.13	0.16	0.01	78.9	0.02	1.18	1.13
**BnF Froehner verre 23**	green	bust & box monogram	Aristomachos	1.42	18.0	after 582	17.9	1.39	3.20	65.7	0.21	0.82	1.01	6.90	0.16	0.72	1.75	7.60	165	37.4	20.4	16.3	24.9	52.8	34.0	75.5	6.56	12.6	567	11.41	83.5	2.98	2.77	0.77	0.04	0.05	6.81	140	0.47	256	12.6	18.9	2.88	11.81	2.39	0.61	2.14	0.34	1.99	0.41	1.10	0.15	1.03	0.16	2.05	0.17	0.48		80.0	0.10	1.78	1.37
**BnF Froehner verre 25**	aqua	2 busts & cruciform monogram	Genethlios	6.34	28.3	615-641	16.7	1.01	2.66	67.2	0.16	0.82	0.81	8.55	0.13	0.73	0.99	5.06	135	24.1	21.3	10.7	13.4	64.0	31.9	73.4	5.43	8.63	580	7.42	68.0	2.28	1.98	0.61	0.11	0.07	10.8	49.2	0.12	258	7.83	13.1	1.74	7.15	1.44	0.40	1.27	0.22	1.24	0.26	0.73	0.10	0.66	0.10	1.63	0.13	0.18	0.09	108	0.03	1.15	1.06
**BnF Froehner verre 31**	yellowish green	box monogram	Mounios / Mousilios	3.64	24.8	6th	17.7	1.23	2.80	65.1	0.11	0.91	0.62	8.92	0.15	1.10	1.10	6.88	159	33.1	21.0	11.5	21.8	40.3	35.2	85.6	6.57	7.84	780	9.32	87.6	2.82	4.34	0.15	0.05	0.05	7.63	188	0.14	293	9.77	15.0	2.10	8.68	1.77	0.43	1.52	0.26	1.54	0.33	0.87	0.14	0.86	0.13	2.08	0.18	0.32	0.01	104	0.05	1.50	1.03
**BnF Froehner verre 32**	greenish	box monogram	Diakonos / Andronikos	1.93	18.3	6th	15.4	0.99	2.73	67.4	0.08	0.79	0.59	9.30	0.21	1.26	0.99	7.33	125	28.6	26.7	5.26	9.39	43.4	27.5	74.0	4.67	8.77	725	8.89	120.5	3.43	1.70	8.41	0.10	0.03	5.39	198	0.13	261	9.12	15.7	2.02	8.32	1.73	0.42	1.38	0.24	1.51	0.31	0.87	0.13	0.80	0.12	2.91	0.21	0.17	0.21	45.7	0.16	1.69	1.29
**BnF Froehner verre 33**	aqua / greenish	cruciform monogram	Prokopios	4.21	27.0	1.00	14.8	0.84	2.09	71.6	0.12	1.24	0.74	6.19	0.13	1.14	0.82	11.8	235	30.0	2.95	8.10	15.7	36.6	31.5	87.9		8.01	522	6.57	80.9	2.42	4.20	0.16	0.20	0.01	3.88	37.3	0.12	311	7.34	11.4	1.55	6.17	1.29	0.25	1.01	0.17	1.04	0.26	0.67	0.10	0.63	0.11	1.79	0.15	0.26		13.1		1.23	1.16
**BnF Froehner verre 35**	amber	box monogram	Oreios / Euresios	4.43	23.2	6th	17.6	0.97	2.40	66.2	0.12	0.87	0.69	8.36	0.13	1.44	0.90	8.41	143	28.3	17.2	5.71	12.1	63.4	22.9	79.9	5.61	8.36	721	7.45	77.1	2.57	3.73	0.09	0.07	0.04	8.14	251	0.13	284	7.74	13.0	1.68	6.86	1.37	0.36	1.21	0.20	1.19	0.27	0.71	0.11	0.67	0.10	1.88	0.13	0.16		69.9	0.03	1.30	1.06
**BnF Froehner verre 37**	yellowish green	box monogram	Addaeos / Aidesios	2.94	21.1	6th	19.0	0.89	2.14	65.6	0.10	0.89	0.61	8.42	0.12	1.17	0.88	6.91	154	23.8	16.5	5.49	8.44	40.3	18.7	67.7	6.55	6.84	640	7.64	72.0	2.30	1.87	0.42	0.04	0.04	8.34	210	0.09	246	7.98	12.8	1.76	7.23	1.43	0.38	1.20	0.21	1.26	0.27	0.75	0.10	0.70	0.11	1.79	0.12	0.15	0.01	70.4	0.03	1.19	1.18
**BnF Froehner verre 40**	amberish	cruciform monogram	Hypatos	1.29	17.5	6th - 7th	16.4	1.09	2.68	67.6	0.16	0.78	0.82	7.46	0.16	1.54	0.99	6.37	165	37.4	19.4	10.0	19.3	43.1	27.8	121	5.56	8.78	661	8.25	93.4	2.81	3.96	0.10	0.08	0.02	4.58	65.8	0.15	442	9.13	14.4	1.94	7.79	1.58	0.41	1.30	0.22	1.36	0.28	0.79	0.11	0.77	0.12	2.22	0.16	0.31	0.01	43.4	0.03	1.50	1.08
**BnF Froehner verre 42**	amberish	bust		3.55	23.7	6th - 7th	18.4	1.04	2.38	66.4	0.14	0.83	0.69	7.58	0.14	1.42	0.82	5.49	145	26.4	14.8	7.15	13.1	52.5	23.2	72.3	5.14	7.83	659	7.15	83.4	2.53	4.68	0.10	0.09	0.04	6.43	104	0.11	263	7.79	13.0	1.68	6.82	1.36	0.37	1.13	0.22	1.18	0.24	0.67	0.10	0.65	0.10	2.00	0.15	0.21		89.6	0.02	1.28	1.03
**BnF inv 65.5417**	aqua bluish	bust & inscription	Theodoros	2.13	21.3	6th - 7th	16.2	1.28	2.65	67.0	0.21	0.78	1.01	9.29	0.12	0.47	0.85	7.72	133	18.5	20.0	9.71	10.8	53.1	18.4	74.9	3.29	7.96	587	6.83	61.3	2.00	1.44	0.38	0.03	0.06	13.6	31.8	0.10	251	6.39	11.1	1.47	6.12	1.25	0.36	1.20	0.19	1.13	0.23	0.64	0.09	0.64	0.09	1.53	0.12	0.13	0.03	99.7	0.02	1.00	0.96
**BnF reg L 4096**	colourless with amber tinge	cruciform monogram	Ioulianos	1.91	21.4	580.00	16.8	1.19	2.72	67.0	0.18	0.75	0.86	7.61	0.15	1.48	1.03	5.37	158	31.4	16.3	9.61	17.8	64.3	26.3	74.1	2.31	8.56	670	7.84	86.9	2.67	4.62	0.52	0.04	0.05	11.0	141	0.10	254	7.95	13.2	1.82	7.34	1.50	0.37	1.30	0.22	1.26	0.28	0.75	0.11	0.75	0.11	2.08	0.15	0.20	0.01	69.0		1.36	1.04
**BnF reg L 4098**	pinkish amber	cruciform monogram	Akakios	4.09	26.0	6th - 7th	17.0	1.41	2.70	65.3	0.22	0.77	0.93	8.07	0.15	2.07	1.12	6.34	155	31.3	17.8	26.3	33.0	63.8	53.4	81.5	5.12	8.53	832	8.34	85.8	2.75	7.17	0.13	0.12	0.04	5.82	56.3	0.14	275	8.73	13.4	1.91	7.81	1.58	0.41	1.42	0.24	1.38	0.30	0.85	0.12	0.83	0.12	2.10	0.15	0.30	0.01	75.1	0.03	1.35	0.96
**BnF reg M 1828**	greenish	box monogram	Paulos	3.77	23.2	565-578	18.4	0.94	2.39	66.3	0.09	0.85	0.58	7.78	0.12	1.56	0.80	6.50	155	28.1	15.6	11.5	13.2	65.9	20.4	98.4	7.59	7.06	637	7.29	69.3	2.22	3.19	0.30	0.07	0.05	7.40	122	0.09	335	6.90	11.4	1.55	6.49	1.37	0.36	1.23	0.20	1.18	0.25	0.68	0.10	0.65	0.09	1.63	0.13	0.19	0.02	112	0.09	1.09	1.01
**BnF reg N 4263 bis**	amber (pinkish)	cruciform monogram	Theophilos	3.70	22.3	6th - 7th	18.9	0.89	2.36	64.8	0.09	0.77	0.52	8.84	0.14	1.48	0.93	5.34	170	37.1	18.1	7.57	9.97	44.4	19.5	103	6.26	6.45	680	8.47	83.8	2.48	2.50	1.40	0.07	0.03	5.88	88.8	0.10	358	8.10	12.9	1.80	7.61	1.55	0.41	1.40	0.22	1.34	0.29	0.78	0.11	0.75	0.12	1.99	0.15	0.15	0.07	39.4	0.03	1.27	1.45
**BnF Schlumberger 3981**	aqua greenish	cruciform monogram	Nikaios / Anikios	4.10	26.7	6th - 7th	17.8	1.37	3.05	66.4	0.22	0.84	0.94	6.90	0.16	0.54	1.60	7.19	169	34.7	19.7	12.5	21.4	51.8	32.5	5.32	6.55	11.5	570	11.56	87.3	2.97	2.72	0.47	0.04	0.05	5.66	138	0.42	224	12.6	18.8	2.83	11.5	2.31	0.63	1.98	0.35	2.02	0.41	1.11	0.16	1.01	0.15	2.07	0.17	0.41	0.02	80.8	0.11	1.85	1.37
**BnF Schlumberger 3985**	aqua greenish	bust & box monogram	Aristomachos	4.17	26.2	after 582	17.2	1.44	2.93	64.2	0.30	0.74	1.06	9.78	0.17	0.43	1.46	8.19	165	29.7	34.7	21.9	19.2	105	61.2	4.79	7.06	9.22	850	9.19	97.5	3.01	1.41	1.71	0.09	0.28	63.3	137	0.12	217	9.49	15.1	2.03	8.54	1.80	0.46	1.57	0.26	1.56	0.32	0.86	0.13	0.87	0.12	2.30	0.17	0.23	0.04	710	0.15	1.59	1.34
**BnF Schlumberger 3986**	amberish	box monogram	Areobindos	1.83	20.1	0.98	16.1	1.14	2.52	68.1	0.18	0.82	0.84	7.54	0.13	1.54	0.90	5.17	158	30.7	27.1	11.9	20.0	76.0	31.1	4.43	3.39	8.43	666	7.15	79.6	2.42	5.14	0.08	0.08	0.03	4.60	71.4	0.11	248	7.35	12.3	1.60	6.76	1.38	0.38	1.21	0.21	1.20	0.26	0.69	0.09	0.67	0.10	1.93	0.15	0.20		107	0.01	1.19	0.99
**BnF Schlumberger 3989**	colourless (pinkish)	cruciform monogram	Addaeos	1.42	20.3	0.98	17.5	1.15	2.51	66.9	0.18	0.87	0.78	7.42	0.14	1.46	0.78	3.21	200	26.5	32.5	5.61	13.3	37.7	25.8	4.47	0.93	6.78	658	6.81	83.3	2.36	4.47	10.22	0.13	0.02	3.28	14.9	0.04	233	6.97	12.0	1.52	6.48	1.34	0.34	1.11	0.18	1.11	0.24	0.66	0.09	0.69	0.09	2.03	0.13	0.11	0.01	43.0		1.20	1.29
**BnF Schlumberger 3990**	colourless	box monogram	Paeitos	1.68	18.6	6th - 7th	18.2	0.68	1.90	68.8	0.04	0.97	0.42	6.58	0.09	1.53	0.62	2.50	154	21.5	25.2	4.18	6.94	22.2	12.4	3.54	0.94	5.61	510	6.36	54.5	1.49	2.61	0.01	0.02	0.00	0.52		0.03	241	6.36	9.90	1.36	5.73	1.15	0.31	1.05	0.19	1.07	0.21	0.62	0.08	0.61	0.09	1.27	0.08	0.14		4.43		0.89	1.01
**BnF Schlumberger 3991**	colourless with amber tinge	box monogram	Ioulianos	1.17	18.8	580.00	16.0	1.19	2.49	68.1	0.20	0.82	0.90	7.02	0.14	1.97	0.91	3.43	172	32.4	34.3	10.3	20.3	67.2	35.5	5.00		7.73	671	7.04	83.0	2.50	6.31	0.24	0.08	0.02	3.59	9.05	0.06	278	7.15	11.9	1.56	6.68	1.30	0.32	1.16	0.19	1.24	0.24	0.65	0.09	0.69	0.10	1.91	0.14	0.19		64.1		1.22	1.00
**BnF Schlumberger 3992**	colourless with amber tinge	box monogram	Dikoros / Dioskoros	1.30	16.7	6th	19.5	0.45	1.94	68.9	0.03	0.95	0.35	6.35	0.08	0.81	0.45	0.86	137	17.9	25.8	3.66	6.38	15.1	12.0	3.36		4.92	451	6.06	51.9	1.43	1.37	0.29	0.03		0.63			312	5.98	9.76	1.26	5.43	1.14	0.25	1.08	0.16	1.01	0.21	0.57	0.08	0.58	0.08	1.24	0.08	0.27		5.66		0.93	1.48
**BnF Schlumberger 3993**	colourless with amber tinge	box monogram	Sisinniolos	1.42	18.6	6th	17.8	0.88	2.35	65.8	0.07	1.12	0.59	9.01	0.13	1.21	0.82	4.18	181	31.2	28.8	7.33	10.3	29.6	22.6	4.57		7.11	681	7.87	82.5	2.40	1.49	0.12	0.07	0.01	4.33	4.59	0.03	270	7.52	12.6	1.61	6.76	1.48	0.37	1.31	0.21	1.26	0.27	0.71	0.11	0.75	0.10	1.93	0.14	0.17		28.6		1.24	1.21
**BnF Schlumberger 3996**	amberish green	box monogram	Addaeos / Aidesios	3.38	20.6	6th	17.6	0.79	2.29	67.6	0.05	0.85	0.46	7.77	0.12	1.54	0.71	4.84	175	23.8	17.3	4.76	7.56	26.0	15.2	3.99	2.87	6.20	543	7.32	71.1	2.12	2.23	0.06	0.05	0.01	2.27	75.6	0.08	256	7.22	12.0	1.57	6.46	1.44	0.33	1.19	0.20	1.11	0.25	0.66	0.10	0.64	0.10	1.61	0.12	0.12	0.01	16.9	0.01	1.12	1.29
**BnF Schlumberger 3998**	amberish	bust & inscription	Iustinianus	1.88	20.4	538-565	16.1	1.17	2.55	66.3	0.11	0.96	0.61	8.82	0.15	1.72	1.18	5.58	170	35.6	20.3	19.4	25.4	86.7	40.1	5.13	7.21	6.65	774	8.69	82.4	2.69	4.01	0.07	0.11	0.07	4.79	309	0.12	244	9.05	13.9	1.89	7.75	1.66	0.39	1.56	0.23	1.46	0.28	0.82	0.11	0.77	0.12	1.95	0.16	0.12		223	0.02	1.30	1.17
**Foy-2 cobalt blue**																																																														
**BM 1882,0510.20**	cobalt blue	bust & inscription	Damianos / Domianos	4.41	24.6	610/630	19.2	1.08	2.27	65.5	0.08	0.95	0.53	8.73	0.14	0.33	0.91	6.78	200	19.2	10.3	109	12.6	162	17.1	3.64	3.96	6.47	663	7.17	80.0	2.54	1.18	0.10	0.02	0.11	9.46	94.9	0.08	185	7.00	12.6	1.65	6.95	1.28	0.35	1.24	0.20	1.18	0.25	0.67	0.09	0.67	0.10	1.89	0.13	0.07		256	0.02	1.13	1.19
**BM 1884,0509.13**	cobalt blue	bust & inscritpion & cusped border	Demosthenos	4.03	23.6	6th - 7th	16.2	1.09	2.40	67.8	0.12	0.82	0.60	8.55	0.15	0.21	1.37	6.35	168	19.2	9.53	883	76.7	1219	35.7	4.19	14.6	7.02	622	7.43	83.9	2.64	2.89	3.86	0.05	0.62	24.2	158	0.10	178	7.44	12.9	1.71	7.46	1.47	0.34	1.30	0.21	1.21	0.24	0.69	0.10	0.66	0.09	2.00	0.14	0.07	0.26	2078	0.10	1.25	1.21

S1 Table: LA-ICP-MS data of the Byzantine glass weights. Major and minor oxides [wt%], including chlorine, and trace elements [ppm]; where the monograms are ambiguous, multiple names are given (Tobias, forthcoming).

Sample Number	colour	type	names	weight [g]	Ø [mm]	Date	Na_2O	MgO	Al_2O_3	SiO_2	P_2O_5	Cl	K_2O	CaO	TiO_2	MnO	Fe_2O_3	Li	B	V	Cr	Co	Ni	Cu	Zn	Ga	As	Rb	Sr	Y	Zr	Nb	Mo	Ag	Cd	In	Sn	Sb	Cs	Ba	La	Ce	Pr	Nd	Sm	Eu	Gd	Tb	Dy	Ho	Er	Tm	Yb	Lu	Hf	Ta	W	Au	Pb	Bi	Th	U
**BM 1884,0509.15**	cobalt blue	bust & box monogram	Akakios	4.03	25.2	6th	18.0	0.95	2.43	65.5	0.09	0.83	0.60	8.00	0.13	1.34	1.35	7.67	144	25.3	16.2	762	100	1205	34.1	5.49	16.9	7.40	610	6.90	66.6	2.14	4.53	1.57	0.09	0.90	19.9	122	0.11	326	6.59	11.3	1.53	6.71	1.34	0.35	1.43	0.20	1.20	0.24	0.67	0.09	0.67	0.09	1.65	0.12	0.20	0.47	2733	0.11	1.07	1.02
**BM 1893,0205.76**	cobalt blue	box monogram* & inscription**	Theodoros / Dorotheos* & Eupraxios**	4.22	24.3	6th - 7th	17.1	1.16	2.40	67.1	0.09	0.97	0.62	8.65	0.14	0.39	1.08	5.86	171	19.2	14.0	367	27.4	262	33.1	4.12	5.36	7.20	628	6.85	74.3	2.41	2.04	0.52	0.11	0.24	19.1	79.3	0.13	183	6.57	11.7	1.55	6.60	1.40	0.36	1.50	0.21	1.17	0.22	0.69	0.09	0.71	0.08	1.83	0.15	0.12	0.04	556	0.04	1.14	1.11
**BM 1893,0409.2**	cobalt blue	3 busts & cruciform monogram	Euthalios / Autheios	2.01	20.0	6th - 7th	17.9	0.94	2.46	64.8	0.09	0.77	0.60	7.73	0.13	1.30	1.42	8.50	149	26.7	18.8	900	225	2619	55.6	5.85	46.0	7.68	573	6.73	66.2	2.19	3.35	0.19	0.05	4.35	18.9	125	0.12	310	6.49	11.4	1.57	6.80	1.35	0.36	1.33	0.19	1.13	0.23	0.66	0.09	0.66	0.09	1.68	0.13	0.19	0.01	10394	0.17	1.04	1.03
**BM 1980,0611.3**	cobalt blue	bust & box monogram	Arkadios / Dakios	1.61	19.6	6th	17.9	0.98	2.50	65.3	0.09	0.82	0.62	8.12	0.13	1.36	1.37	7.80	141	25.6	16.2	822	109	1302	37.2	5.68	17.1	7.51	622	7.15	70.0	2.20	4.70	2.08	0.12	0.96	19.3	120	0.11	329	6.79	11.5	1.57	6.90	1.46	0.37	1.45	0.22	1.18	0.24	0.70	0.10	0.73	0.10	1.77	0.14	0.19	0.64	2873	0.12	1.12	1.04
**BM 1980,0611.9**	cobalt blue	box monogram	Andronikos	4.18	23.7	6th	18.7	0.85	2.35	65.7	0.08	0.74	0.60	7.54	0.18	0.48	2.06	6.22	141	24.5	21.5	1040	110	1395	49.7	6.92	12.6	6.35	593	6.92	99.7	2.57	12.1	2.31	0.06	0.91	40.9	29.8	0.07	202	7.06	12.4	1.55	6.66	1.31	0.33	1.15	0.18	1.13	0.24	0.68	0.09	0.68	0.10	2.35	0.14	0.13	0.09	1672	0.23	1.31	1.37
**BM 1980,0611.10**	cobalt blue	box monogram	Eudaimonos	4.32	24.0	6th	17.3	1.09	2.50	63.9	0.13	0.78	0.68	8.80	0.15	1.44	2.30	8.65	155	30.1	18.2	1098	121	2163	207	6.01	12.0	7.85	687	7.80	82.7	2.66	11.3	0.41	0.05	1.14	91.4	134	0.13	306	8.06	13.6	1.71	7.50	1.53	0.36	1.29	0.22	1.32	0.26	0.75	0.09	0.73	0.10	2.06	0.15	0.18	0.02	2202	0.21	1.39	1.14
**BM 1980,0611.11**	cobalt blue	box monogram	Pelagios / Paeitos	3.22	21.7	6th	19.5	1.15	2.33	63.3	0.12	0.73	0.56	9.22	0.15	0.61	1.32	7.00	178	22.4	12.3	524	73.9	855	29.8	4.21	18.0	5.49	695	7.45	86.7	2.54	3.15	0.10	0.03	0.85	21.0	68.2	0.08	207	7.35	12.6	1.65	7.20	1.47	0.36	1.43	0.20	1.22	0.26	0.73	0.10	0.67	0.10	2.08	0.14	0.08	0.01	5426	0.20	1.26	1.38
**BM 1980,0611.16**	cobalt blue	box monogram	Andronikos	1.47	15.8	6th	17.5	1.14	2.46	64.2	0.15	0.79	0.75	9.43	0.17	0.57	2.28	7.27	135	25.3	19.0	659	65.4	959	60.9	7.32	12.7	7.14	731	7.73	94.1	2.71	11.4	0.32	0.02	1.03	84.2	81.6	0.09	217	7.96	13.7	1.77	7.47	1.54	0.38	1.28	0.20	1.32	0.26	0.74	0.10	0.73	0.11	2.17	0.17	0.14	0.01	1370	0.38	1.45	1.26
**BM 1980,0611.20**	cobalt blue	box monogram		1.31	17.6	6th	15.2	1.26	3.08	58.8	0.10	0.63	0.74	8.19	0.16	1.30	4.09	10.1	143	37.1	25.0	5955	1122	5463	339	19.0	286	14.1	596	7.67	72.7	2.66	16.1	0.78	0.32	22.19	303	191	0.50	308	7.98	13.7	1.80	7.54	1.56	0.39	1.49	0.22	1.34	0.26	0.76	0.11	0.75	0.10	1.81	0.16	0.22	0.03	41650	0.69	1.44	1.03
**BM 1980,0611.25**	cobalt blue	cruciform monogram	Neophytos	4.52	25.0	6th - 7th	15.8	1.27	2.97	67.2	0.19	0.81	0.92	8.59	0.18	0.23	1.23	6.67	161	21.3	20.5	165	50.9	733	26.0	5.21	12.3	8.65	612	7.35	100.9	3.03	1.98	1.67	0.04	0.51	44.8	24.7	0.13	194	7.36	13.2	1.68	7.38	1.54	0.37	1.51	0.21	1.32	0.24	0.73	0.10	0.73	0.10	2.52	0.18	0.10		2389	0.05	1.43	1.03
**BM 1980,0611.26**	cobalt blue	cruciform monogram	Neophytos	4.46	24.5	6th - 7th	16.0	0.68	2.61	71.7	0.05	0.85	0.62	5.71	0.15	0.07	1.03	4.98	164	16.4	14.0	240	53.2	776	20.8	5.27	10.2	8.03	395	6.21	86.4	2.34	2.95	0.57	0.03	0.62	51.1	2.38	0.10	156	6.27	11.3	1.45	6.35	1.33	0.33	1.19	0.19	1.10	0.22	0.68	0.08	0.61	0.09	2.09	0.12	0.07	0.02	2610	0.04	1.16	0.94
**BM 1980,0611.30**	cobalt blue	3 busts & cruciform monogram	Euthalios / Autheios	3.81	24.0	6th - 7th	17.7	0.94	2.51	64.7	0.09	0.80	0.59	7.81	0.13	1.29	1.47	8.17	148	26.8	17.5	948	237	2798	56.6	6.01	50.8	7.65	578	7.00	68.0	2.21	3.52	0.10	0.06	4.59	19.1	126	0.12	310	6.69	11.4	1.60	6.60	1.34	0.37	1.35	0.21	1.17	0.23	0.67	0.09	0.66	0.10	1.73	0.13	0.19	0.01	11951	0.19	1.09	1.01
**BM 1980,0611.31**	cobalt blue	cruciform monogram	Sergios	4.50	22.8	6th - 7th	19.6	1.05	2.70	64.4	0.12	0.91	1.15	7.14	0.14	0.22	1.51	5.63	175	22.8	5.81	580	189	1055	38.8	6.61	19.6	7.31	562	7.24	84.5	2.45	6.93	0.25	0.01	0.88	50.6	69.7	0.13	175	7.42	12.6	1.69	7.51	1.56	0.39	1.38	0.21	1.27	0.25	0.73	0.10	0.69	0.11	2.11	0.13	0.16		6481	0.10	1.32	1.26
**BM 1980,0611.37**	cobalt blue	cruciform monogram	Ioannos	1.52	18.5	6th - 7th	15.4	1.26	3.02	67.8	0.18	0.76	0.95	8.46	0.20	0.20	1.20	6.58	158	22.2	12.5	154	48.9	636	28.0	5.19	11.7	9.31	587	7.42	103.9	3.19	1.95	0.75	0.04	0.48	42.7	17.5	0.12	195	7.67	14.0	1.76	7.57	1.59	0.34	1.46	0.20	1.34	0.26	0.77	0.11	0.80	0.12	2.54	0.18	0.11	0.03	2208	0.05	1.52	1.05
**BM 1980,0611.38**	cobalt blue	cruciform monogram	Ioulianos	1.57	17.8	6th - 7th	15.4	0.72	2.69	71.9	0.06	0.63	0.63	5.87	0.15	0.08	1.15	4.07	161	16.9	17.0	267	59.3	1265	48.4	5.51	9.53	8.30	392	6.11	84.1	2.33	2.95	0.09	0.12	0.66	57.6	1.47	0.20	158	6.33	11.0	1.44	6.43	1.30	0.32	1.26	0.18	1.06	0.21	0.63	0.08	0.59	0.08	2.07	0.14	0.07		2807	0.13	1.21	0.98
**BM 1980,0611.39**	cobalt blue	cruciform monogram	Ioulianos	1.51	17.1	6th - 7th	15.5	0.75	2.70	71.1	0.05	0.84	0.59	6.06	0.16	0.08	1.31	5.23	160	17.7	16.6	444	88.3	1326	35.6	7.00	14.7	7.31	394	6.46	87.0	2.43	5.52	4.33	0.11	1.15	93.9	3.30	0.09	159	6.51	11.6	1.50	6.54	1.38	0.33	1.29	0.19	1.09	0.22	0.64	0.09	0.67	0.09	2.17	0.15	0.08	0.96	4607	0.07	1.19	0.93
**BM 1980,0611.41**	cobalt blue	cruciform monogram	Sergios	1.44	19.8	6th - 7th	20.1	0.95	2.67	65.2	0.08	0.87	0.55	7.13	0.16	0.13	1.33	5.62	173	18.8	10.1	396	136	1556	31.0	5.95	18.9	6.39	497	6.93	90.4	2.56	4.79	0.19		0.83	58.2	12.6	0.10	157	7.00	12.2	1.58	6.77	1.42	0.37	1.28	0.20	1.21	0.25	0.70	0.09	0.67	0.10	2.21	0.14	0.10	0.03	3868	0.07	1.30	1.32
**BM 1980,0611.42**	cobalt blue	cruciform monogram	Ioannos	2.25	19.7	6th - 7th	16.2	1.21	2.88	67.6	0.17	0.78	0.84	8.12	0.18	0.22	1.21	6.29	159	20.9	19.5	198	57.9	827	24.4	5.18	9.33	8.25	565	6.90	96.9	2.94	2.17	0.12	0.03	0.51	44.2	27.7	0.12	189	7.22	13.2	1.67	7.13	1.47	0.35	1.35	0.20	1.24	0.24	0.67	0.09	0.73	0.10	2.39	0.16	0.11		2521	0.04	1.41	1.02
**BM 1980,0611.45**	cobalt blue	cruciform monogram	Hypatos	1.51	17.4	6th - 7th	19.5	0.74	2.29	68.1	0.05	0.98	0.55	6.11	0.12	0.10	0.85	4.85	182	14.8	11.0	270	69.8	1105	20.4	5.30	11.5	6.79	441	5.79	76.2	2.02	3.02	0.07	0.04	0.91	69.8	4.34	0.08	149	6.04	10.6	1.34	5.83	1.18	0.29	1.03	0.17	0.96	0.19	0.55	0.08	0.58	0.08	1.90	0.11	0.06		3221	0.05	1.06	1.04
**BM 1980,0611.51**	cobalt blue	bust & inscription	possibly Cufic	4.18	23.0	650 - 700	17.4	1.08	2.52	63.5	0.13	0.76	0.68	8.65	0.15	1.42	2.74	8.78	158	28.5	16.0	1868	221	1558	168	7.40	12.3	8.03	669	7.31	76.2	2.49	11.4	0.15	0.04	1.38	90.8	133	0.16	304	7.36	12.8	1.65	7.15	1.45	0.36	1.43	0.19	1.23	0.25	0.72	0.09	0.74	0.10	1.81	0.13	0.18	0.01	2622	0.23	1.30	1.09
**BM 1980,0611.52**	cobalt blue	bust & inscription	possibly Cufic	4.27	23.0	650 - 700	18.1	0.98	2.45	64.8	0.10	0.78	0.58	8.31	0.14	1.25	1.54	7.55	158	27.1	14.0	512	90.4	2501	35.6	6.46	13.5	6.79	616	7.23	74.1	2.17	6.38	1.06	0.07	0.56	53.3	94.3	0.10	303	7.03	11.9	1.56	6.87	1.40	0.34	1.33	0.19	1.27	0.25	0.71	0.09	0.68	0.10	1.80	0.14	0.17	0.09	3289	0.17	1.18	1.10
**BM 1980,0611.57**	cobalt blue	bust & inscription		1.16	19.5	6th - 7th	19.1	1.26	2.72	64.8	0.19	0.84	0.91	7.79	0.15	0.21	1.27	5.62	169	21.3	13.8	488	154	1466	32.6	5.21	18.0	6.81	593	7.10	86.8	2.53	3.46	0.15	0.03	0.64	39.4	59.7	0.12	178	7.16	12.3	1.62	7.11	1.51	0.35	1.31	0.21	1.19	0.25	0.68	0.10	0.66	0.10	2.12	0.15	0.14	0.01	2983	0.06	1.27	1.25
**BM 1980,0611.59**	cobalt blue	bust		0.37	12.3	650 - 700	18.0	0.92	2.35	65.9	0.09	0.84	0.60	7.88	0.13	1.31	1.29	8.03	147	25.2	10.9	760	102	1242	35.8	5.46	35.2	7.45	605	6.92	69.1	2.21	4.67	0.98	0.07	0.95	19.0	124	0.12	314	6.98	12.0	1.54	6.90	1.38	0.33	1.35	0.19	1.12	0.24	0.66	0.09	0.65	0.10	1.60	0.12	0.19	0.47	2794	0.11	1.15	1.00
**BM 1980,0611.65**	cobalt blue	bust		4.03	23.7	650 - 700	18.9	0.97	2.38	64.6	0.10	0.90	0.54	8.72	0.13	0.75	1.43	6.18	164	21.3	15.0	917	66.1	518	37.8	4.88	8.71	5.93	694	7.03	72.5	2.26	3.89	0.16	0.06	0.60	18.0	92.3	0.09	223	6.82	11.8	1.57	7.00	1.41	0.36	1.30	0.20	1.17	0.24	0.69	0.10	0.69	0.09	1.81	0.13	0.10	0.01	1239	0.11	1.11	1.11
**BM 1980,1204.1**	cobalt blue	2 busts & box monogram	Sophronios / Photeinos	1.40	18.2	6th	18.0	1.00	2.43	64.9	0.11	0.79	0.60	8.10	0.14	1.25	1.59	8.01	156	27.6	12.9	811	135	1997	76.2	5.65	26.9	7.50	610	7.06	72.1	2.35	6.09	0.31	0.04	2.06	46.8	112	0.11	297	6.80	11.8	1.61	6.84	1.43	0.34	1.36	0.20	1.20	0.24	0.68	0.10	0.64	0.10	1.76	0.13	0.20	0.04	4733	0.16	1.14	1.10
**BM 1983,1108.1**	cobalt blue	bust & inscription	Damianos / Domianos	4.41	24.8	0.97	20.1	1.14	2.52	64.3	0.09	1.06	0.55	8.69	0.14	0.25	0.90	5.49	217	17.8	29.0	104	12.8	158	14.9	3.81	5.31	6.35	636	6.69	75.7	2.37	0.83	0.23	0.04	0.10	8.66	131	0.07	174	6.27	11.2	1.48	6.58	1.39	0.37	1.59	0.21	1.18	0.23	0.69	0.09	0.65	0.09	1.92	0.14	0.06	0.01	250	0.03	1.08	1.06
**BM 1986,0406.3**	cobalt blue	box monogram		2.13	20.1	6th	18.1	1.15	2.55	66.2	0.06	0.93	0.59	8.53	0.14	0.18	1.06	6.44	187	18.3	18.1	169	60.5	858	24.8	4.76	25.3	7.50	618	7.16	81.2	2.56	1.50	0.77	0.04	0.73	5.39	278	0.11	159	7.14	12.4	1.63	7.14	1.48	0.35	1.42	0.20	1.31	0.25	0.72	0.10	0.72	0.09	2.08	0.16	0.08	0.01	1436	0.08	1.28	1.09
**BM 1986,0406.5**	cobalt blue	cruciform monogram		1.50	17.5	6th - 7th	16.1	1.07	2.80	68.6	0.13	0.67	0.93	7.96	0.18	0.16	1.03	6.83	156	19.9	8.96	90	30.2	370	31.3	4.60	7.58	7.72	580	7.22	100.0	2.96	1.35	15.35	0.04	0.31	24.8	21.6	0.11	184	7.74	13.6	1.69	7.40	1.55	0.34	1.24	0.20	1.18	0.24	0.68	0.09	0.74	0.09	2.43	0.16	0.09	0.22	1229	0.03	1.39	1.01
**BM 1986,0406.11**	cobalt blue	box monogram & inscription	Eupraxios	3.92	22.5	6th - 7th	18.2	1.06	2.33	65.0	0.09	0.82	0.59	8.42	0.14	1.42	1.37	6.79	177	25.8	15.4	891	50.2	1015	46.7	5.47	7.31	6.63	675	7.02	74.4	2.32	3.83	0.16	0.04	0.41	11.7	136	0.11	345	6.74	11.6	1.59	6.91	1.36	0.37	1.44	0.20	1.16	0.24	0.66	0.09	0.71	0.09	1.89	0.13	0.15	0.01	1070	0.06	1.10	1.13
**BM 1986,0406.14**	cobalt blue	bust & inscription		3.27	23.2	6th - 7th	17.9	0.99	2.51	65.4	0.10	0.76	0.58	8.00	0.13	1.33	1.07	8.41	159	26.3	16.7	864	185	1640	48.2	5.44	54.7	7.33	596	6.91	69.7	2.20	3.34	0.51	0.06	4.54	20.6	110	0.14	299	6.80	11.7	1.58	6.99	1.31	0.36	1.41	0.21	1.22	0.25	0.70	0.09	0.66	0.09	1.80	0.13	0.16	0.06	6648	0.12	1.10	1.07
**BM 1986,0406.19**	cobalt blue	3 busts & cruciform monogram		0.61	19.1	6th - 7th	18.7	1.13	2.39	65.6	0.09	0.98	0.53	8.62	0.14	0.40	1.05	6.31	169	19.2	11.0	315	24.4	292	46.5	3.86	4.80	6.94	621	7.15	78.0	2.47	1.79	0.59	0.05	0.20	15.4	83.9	0.11	185	6.83	12.1	1.62	6.99	1.42	0.35	1.39	0.20	1.18	0.25	0.67	0.09	0.69	0.10	1.96	0.14	0.12	0.01	539	0.03	1.22	1.16
**BM 1987,0703.1**	cobalt blue	box	Andronikos	1.92	18.9	6th	17.3	1.10	2.73	64.2	0.14	0.78	0.72	9.67	0.18	0.57	2.10	7.58	130	26.2	20.4	568	56.2	648	63.4	6.86	11.1	8.02	751	8.19	98.4	2.93	9.98	2.23	0.10	0.93	66.3	90.1	0.15	442	8.47	14.6	1.83	7.87	1.66	0.39	1.38	0.23	1.34	0.27	0.77	0.10	0.79	0.11	2.39	0.17	0.14	0.34	1033	0.27	1.56	1.23
**BM 1987,0703.7**	cobalt blue	box monogram* & inscription**	Theodoros / Dorotheos* & Eupraxios**	4.25	24.9	6th - 7th	16.7	0.97	2.38	66.1	0.10	0.92	0.69	8.73	0.13	1.36	1.30	4.34	180	24.2	24.5	830	46.4	893	51.9	6.10	7.71	7.48	643	6.74	75.5	2.34	3.48	0.18	0.09	0.44	12.3	143	0.13	316	6.38	10.8	1.48	6.52	1.30	0.29	1.48	0.20	1.12	0.23	0.66	0.09	0.60	0.10	1.83	0.14	0.20	0.01	1200	0.07	1.12	1.15
**BM 1987,0703.20**	cobalt blue	bust & inscritpion & cusped border	Demosthenos	3.95	24.7	6th - 7th	16.2	1.12	2.28	67.6	0.14	0.83	0.63	8.20	0.14	0.22	1.69	6.10	169	19.1	13.3	1482	122	1617	43.8	4.46	10.7	7.06	592	6.94	78.1	2.53	4.57	0.14	0.03	0.84	26.2	164	0.10	168	7.04	12.4	1.64	6.93	1.40	0.34	1.32	0.20	1.20	0.23	0.67	0.09	0.67	0.10	1.92	0.14	0.08	0.01	3079	0.11	1.22	1.19
**BM 1987,0703.21**	cobalt blue	2 busts & box monogram	Sophronios / Photeinos	3.48	26.3	6th	19.2	0.99	2.53	63.7	0.09	0.70	0.61	8.09	0.14	1.29	1.52	8.50	186	27.4	21.8	842	137	1961	68.8	5.47	27.3	7.71	663	6.95	74.3	2.29	5.67	0.37	0.02	2.18	58.8	108	0.12	315	7.15	12.6	1.57	6.90	1.43	0.37	1.30	0.19	1.25	0.26	0.74	0.10	0.74	0.11	1.87	0.12	0.15	0.05	5269	0.17	1.31	1.19
**BM 1990,0601.12**	cobalt blue	box monogram square die	Konstantinos	1.75	18.1	6th	17.7	1.15	2.61	64.2	0.15	0.79	0.66	9.27	0.16	0.57	2.18	6.71	138	24.0	18.0	632	62.2	821	55.1	7.00	11.2	6.74	714	7.61	90.8	2.64	9.56	0.31	0.07	0.92	77.7	82.6	0.09	210	7.19	12.4	1.65	7.20	1.50	0.35	1.39	0.21	1.32	0.25	0.75	0.10	0.73	0.10	2.19	0.15	0.14	0.02	1226	0.36	1.30	1.19
**BM 1997,0218.2**	cobalt blue	bust & inscritpion & cusped border	Theodoros	4.48		6th - 7th	17.7	1.06	2.33	64.7	0.32	0.84	0.71	8.82	0.15	1.41	1.13	6.37	179	27.6	16.6	731	70.7	1628	64.6	4.69	13.8	7.59	687	7.74	86.3	2.59	4.29	0.16	0.06	1.28	86.3	137	0.11	352	8.08	13.4	1.75	7.67	1.53	0.36	1.37	0.21	1.25	0.25	0.71	0.09	0.69	0.10	2.02	0.15	0.20	0.04	3363	0.24	1.33	1.29
**BM 1997,0218.3**	cobalt blue	bust & box monogram	Arkadios / Dakios	4.13	23.2	6th	17.0	0.92	2.46	67.1	0.10	0.98	0.86	7.92	0.12	0.51	1.26	6.70	125	18.7	17.9	330	106	1053	75.5	4.59	25.2	7.46	611	6.43	67.0	2.14	1.69	6.18	0.05	2.22	175	141	0.08	193	6.03	10.7	1.44	6.38	1.43	0.32	1.47	0.19	1.07	0.23	0.60	0.09	0.63	0.10	1.72	0.11	0.14	0.01	3336	0.09	1.07	0.88
**BM 1997,0218.4**	cobalt blue	3 busts & cruciform monogram	Euthalios / Autheios	4.59	24.5	6th - 7th	17.8	0.92	2.42	64.8	0.09	0.80	0.63	7.71	0.13	1.28	1.44	8.05	148	26.9	11.6	939	234	2122	59.1	5.96	51.8	8.11	577	7.08	68.4	2.27	3.63	0.08	0.03	4.59	18.4	128	0.13	308	6.90	11.9	1.59	6.77	1.39	0.37	1.32	0.20	1.17	0.23	0.67	0.09	0.70	0.09	1.70	0.12	0.19	0.01	12730	0.19	1.12	1.05
**BnF AA VA 14**	cobalt blue	2 busts		1.37	16.7	6th - 7th	15.8	0.67	2.58	71.5	0.06	0.86	0.54	5.27	0.15	0.11	1.42	4.98	169	18.5	20.4	460	110	1379	30.2	51.2	18.0	7.41	360	6.76	88.7	2.45	9.82	0.60	0.01	1.55	124	18.2	0.08	172	6.94	12.3	1.55	6.50	1.26	0.35	1.12	0.20	1.14	0.23	0.67	0.09	0.62	0.10	2.19	0.15	0.11	0.06	5984	0.07	1.23	1.02
**BnF AA VA 57**	cobalt blue	bust & inscription	Theodoros	2.14	20.0	6th - 7th	16.0	0.96	2.23	67.4	0.11	0.75	0.59	7.68	0.13	1.29	1.94	6.99	152	27.2	17.4	1019	112	1998	232	5.84	9.49	7.40	633	7.29	75.4	2.49	9.76	0.30	0.09	1.02	90.6	141	0.11	273	7.47	12.8	1.62	6.85	1.34	0.34	1.14	0.20	1.22	0.25	0.70	0.10	0.68	0.10	1.81	0.14	0.18	0.01	2224	0.19	1.33	1.12
**BnF Froehner verre 14**	cobalt blue	box monogram* & inscription**	Theodoros / Dorotheos* & Eupraxios**	3.75	22.0	6th	17.8	1.06	2.37	65.2	0.10	0.95	0.56	8.44	0.14	1.35	1.35	7.69	195	24.9	16.8	993	56.4	1197	61.4	98.8	7.48	6.46	697	7.73	79.9	2.40	4.00	0.19	0.10	0.53	13.5	154	0.06	335	7.87	13.0	1.70	7.06	1.42	0.37	1.25	0.22	1.19	0.26	0.71	0.10	0.71	0.10	1.88	0.14	0.14	0.01	1355	0.07	1.28	1.15
**BnF Froehner verre 15**	cobalt blue	box monogram* & inscription**	Theodoros / Dorotheos* & Eupraxios**	1.97	19.0	6th	17.8	1.09	2.38	65.5	0.09	0.85	0.57	8.49	0.14	1.41	1.21	7.38	184	25.6	17.6	680	41.3	832	42.3	102	6.41	6.56	709	7.59	80.7	2.58	3.73	0.19	0.05	0.31	11.6	149	0.08	352	7.77	13.0	1.73	7.09	1.41	0.36	1.22	0.21	1.27	0.27	0.72	0.10	0.71	0.10	1.93	0.14	0.18	0.06	848	0.05	1.29	1.18
**BnF Froehner verre 16**	cobalt blue	bust & inscritpion & cusped border	Demosthenos	4.44	23.0	6th - 7th	16.1	1.07	2.52	67.7	0.10	0.81	0.68	8.35	0.15	0.19	1.54	6.12	166	19.3	20.2	1084	89.3	892	45.4	49.5	12.8	7.66	607	7.42	83.3	2.73	3.59	15.15	0.04	0.96	22.3	158	0.11	164	7.80	13.8	1.77	7.30	1.47	0.38	1.30	0.21	1.21	0.26	0.69	0.10	0.69	0.10	2.02	0.15	0.09	0.54	2578	0.09	1.36	1.22
**BnF Froehner verre 17**	cobalt blue	bust & inscritpion & cusped border	Demosthenos	4.14	24.0	6th - 7th	16.0	1.12	2.48	67.7	0.13	0.81	0.71	8.25	0.14	0.21	1.54	6.82	164	19.2	19.1	1269	109	1393	39.9	50.2	11.5	7.79	615	7.72	82.6	2.65	3.65	7.91	0.03	0.83	22.3	165	0.30	171	9.62	17.5	2.21	8.91	1.92	0.38	1.54	0.24	1.32	0.27	0.70	0.10	0.70	0.10	2.00	0.16	0.09	0.93	2759	0.13	1.41	1.25

S1 Table: LA-ICP-MS data of the Byzantine glass weights. Major and minor oxides [wt%], including chlorine, and trace elements [ppm]; where the monograms are ambiguous, multiple names are given (Tobias, forthcoming).

Sample Number	colour	type	names	weight [g]	Ø [mm]	Date	Na_2O	MgO	Al_2O_3	SiO_2	P_2O_5	Cl	K_2O	CaO	TiO_2	MnO	Fe_2O_3	Li	B	V	Cr	Co	Ni	Cu	Zn	Ga	As	Rb	Sr
**BnF Froehner verre 18**	cobalt blue	bust & inscritpion & cusped border	Demosthenos	2.05	20.0	6th - 7th	17.4	1.01	2.44	64.9	0.11	0.75	0.56	8.38	0.14	1.28	1.95	6.49	159	26.7	21.6	1699	174	1903	66.5	99.5	15.0	6.89	658
**BnF Froehner verre 22**	cobalt blue	bust & box monogram	Arkadios / Dakios	4.11	23.8	6th	17.8	0.90	2.48	65.8	0.10	0.77	0.60	7.84	0.13	1.25	1.47	6.76	140	27.5	17.3	587	113	1625	38.1	91.3	20.9	7.64	605
**BnF Froehner verre 24**	cobalt blue	3 busts & cruciform monogram	Euthalios / Autheios	1.42	17.2	6th - 7th	17.7	0.90	2.43	65.1	0.10	0.86	0.94	7.34	0.13	1.23	1.35	8.15	158	25.9	20.8	897	226	2443	58.8	87.6	53.8	7.91	564
**BnF Froehner verre 38**	cobalt blue	box monogram square die	Konstantinos	4.38	23.4	6th	17.7	1.08	2.55	64.2	0.15	0.78	0.72	9.33	0.17	0.56	2.21	6.75	128	26.2	20.8	692	66.2	798	57.6	64.2	12.5	7.23	746
**BnF Froehner verre 39**	cobalt blue	box monogram	Konstantinos	3.54	22.1	6th - 7th	17.8	0.99	2.36	65.6	0.17	0.66	0.72	8.89	0.16	1.05	1.18	6.10	146	26.8	20.2	261	36.5	986	43.8	73.8	8.46	6.16	742
**BnF Froehner verre 41**	cobalt blue	bust		3.50	23.5	6th - 7th	17.8	0.93	2.40	66.5	0.09	0.97	0.58	7.84	0.13	0.41	1.42	6.15	119	18.2	16.6	469	131	1095	49.1	54.9	31.4	7.96	556
**BnF Schlumberger 3958**	cobalt blue	S	mark S	1.03	15.7	6th - 7th	16.5	0.90	2.19	67.5	0.09	0.73	0.53	7.54	0.13	1.15	1.81	6.54	152	25.1	16.4	1750	174	1834	58.9	5.91	15.9	6.87	606
**BnF Schlumberger 3977**	cobalt blue	bust & inscription	Damianos / Domianos	4.54	25.0	610/630	20.3	0.71	2.08	67.1	0.07	1.02	0.54	6.96	0.11	0.13	0.65	5.03	128	13.2	12.5	95	26.2	381	11.8	3.58	7.02	6.14	511
**BnF Schlumberger 3982**	cobalt blue	cruciform monogram	Andreos	4.52	26.9	6th - 7th	19.5	0.54	1.99	69.1	0.04	1.09	0.48	5.63	0.11	0.04	0.68	4.05	165	12.5	14.5	280	85.1	1291	19.2	5.15	17.1	6.51	400
**BnF Schlumberger 3984**	cobalt blue	box monogram* & inscription**	Theodoros / Dorotheos* & Eupraxios**	2.23	20.4	6th - 7th	17.7	1.04	2.27	65.7	0.11	0.85	0.58	8.28	0.14	1.37	1.33	7.75	178	26.0	16.0	1030	56.5	1183	58.2	6.32	9.22	6.97	708
**BnF Schlumberger 3988**	cobalt blue	cruciform monogram	Ioannos	1.52	17.8	6th - 7th	16.2	0.70	2.44	71.6	0.06	0.85	0.58	5.83	0.14	0.09	0.94	4.53	171	16.7	26.7	194	46.6	779	21.4	4.96	7.73	6.95	424
**Foy-2 high Fe**																													
**BM 1874,0311.4**	olive green	bust & inscription		1.93	19.8	6th - 7th	18.1	1.32	2.70	64.1	0.22	0.88	0.86	8.09	0.15	1.01	2.32	5.70	172	46.5	16.0	16.0	25.8	75.8	39.2	4.86	12.1	7.76	660
**BM 1882,0510.34**	olive green	cruciform monogram	Timotheos	1.48	21.0	6th - 7th	17.4	1.47	3.05	62.3	0.28	0.81	0.96	8.32	0.17	1.31	3.63	7.04	160	73.2	13.5	19.9	29.8	99.1	52.7	5.90	20.5	9.02	677
**BM 1891,0512.11**	olive green	bust & inscription	Theodotos	1.17	19.4	541/542 546/547	17.5	1.08	2.70	65.7	0.18	0.83	0.73	6.72	0.15	1.12	3.06	6.09	174	68.2	13.9	18.3	28.0	90.8	47.4	5.03	19.1	8.91	549
**BM 1923,1107.1**	olive green	cruciform monogram	Timotheos	2.02	21.2	6th - 7th	18.1	1.28	2.75	64.7	0.21	0.89	0.85	7.10	0.16	1.02	2.66	6.18	173	58.8	11.1	16.0	26.2	79.5	38.3	4.89	13.4	8.12	559
**BM 1980,0611.33**	olive green	cruciform monogram	Timotheos	2.19	20.4	6th - 7th	19.0	1.30	2.84	63.9	0.19	0.92	0.82	7.18	0.16	0.95	2.55	6.72	197	53.3	11.2	16.1	22.9	79.1	40.2	5.09	14.2	8.60	574
**BM 1980,0611.48**	olive green	bust & inscription	Theodotos	2.22	22.0	541/542 546/547	17.7	0.89	2.63	67.8	0.12	0.81	0.66	5.60	0.17	0.96	2.56	6.14	182	57.8	14.7	15.5	23.8	53.8	38.8	4.74	16.7	8.80	461
**BM 1980,0611.55**	olive green	bust & inscription		2.06	19.2	6th - 7th	16.7	1.57	2.62	63.8	0.33	0.77	1.25	7.78	0.22	1.66	2.92	6.99	158	68.1	25.2	17.9	31.6	105	48.0	5.28	13.2	8.64	658
**BM 1980,0611.75**	olive green	S	mark S	1.11	16.9	6th - 7th	17.2	1.36	2.91	64.7	0.20	0.77	0.77	7.68	0.18	0.95	3.02	7.08	197	58.4	18.1	16.6	24.4	98.4	42.7	5.33	16.9	8.45	591
**BM 1986,0406.12**	olive green	bust & inscription	Theodotos	1.98	20.5	541/542 546/547	16.1	1.52	2.88	63.2	0.28	0.68	1.18	9.15	0.18	1.26	3.23	11.1	179	68.0	16.3	69.9	40.3	269	55.2	5.85	19.7	9.45	855
**BM 1986,0406.13**	olive green	bust & inscription	Kyrionymos	2.16	20.0	6th - 7th	15.4	1.23	2.73	66.7	0.27	0.74	0.99	7.42	0.18	0.87	3.28	6.17	164	66.2	10.9	12.0	19.8	82.3	41.0	4.82	18.7	8.74	547
**BM 1987,0703.8**	olive green	cruciform monogram	Timotheos	2.21	20.7	6th - 7th	17.5	1.13	2.64	65.5	0.21	0.89	0.83	6.65	0.15	1.12	3.13	5.96	182	72.9	8.20	25.7	29.2	136	43.8	5.17	18.7	8.33	546
**BM 1987,0703.9**	olive green	cruciform monogram	Timotheos	1.22	20.0	6th - 7th	18.1	1.26	2.67	64.6	0.20	0.93	0.84	7.23	0.16	1.01	2.73	5.88	177	61.5	8.89	16.3	26.9	90.0	39.9	4.96	14.9	8.38	591
**BM 1987,0703.10**	olive green	cruciform monogram	Akakios	3.84	26.2	6th - 7th	19.1	1.25	2.70	63.6	0.20	0.85	0.81	7.46	0.14	1.36	2.28	6.31	165	51.0	17.3	17.9	34.0	77.0	43.6	4.83	10.6	8.28	643
**BM 1987,0703.13**	olive green	cruciform monogram	Theodoros / Dorotheos	1.30	18.0	600-625	19.0	1.12	2.66	64.3	0.17	0.89	0.75	7.19	0.16	0.98	2.53	5.81	172	49.2	15.8	14.4	23.7	66.4	39.4	4.84	13.7	7.71	560
**BM 1990,0601.16**	blue-green	bust & box monogram	Aristomachos	1.35	18.5	after 582	18.3	1.28	3.05	64.3	0.22	0.90	0.96	7.20	0.16	0.85	2.48	7.34	176	48.3	16.1	15.2	25.5	69.5	39.4	5.13	10.6	11.3	592
**BM 1990,0601.19**	olive green	bust & inscription	Ioustinos	2.01	19.2	6th - 7th	18.5	1.23	2.65	63.3	0.23	0.80	0.89	8.03	0.16	1.09	2.83	6.44	167	55.9	15.9	16.1	25.6	83.4	42.0	4.73	14.9	7.80	642
**BM 1990,0601.20**	olive green	bust & inscription	Kyrionymos	3.69	26.8	6th - 7th	18.5	1.30	2.58	64.1	0.23	0.87	0.88	7.80	0.15	1.04	2.37	8.08	174	48.5	7.75	16.0	26.5	77.7	43.5	4.83	13.3	7.91	632
**BnF AA de Clercq 7**	aqua bluish	box monogram	Pakianos	1.49	18.4	6th	16.6	1.43	3.58	64.5	0.19	0.85	0.83	7.81	0.18	1.09	2.64	6.74	161	57.4	21.2	18.9	33.6	72.6	47.8	6.15	12.0	12.6	672
**BnF AA VA 17**	yellowish green	cruciform monogram	Megethios	2.07	20.2	6th - 7th	16.0	1.44	3.32	64.5	0.23	0.91	1.53	8.23	0.17	0.98	2.35	12.7	167	51.6	19.9	17.0	28.3	39.3	53.6	104	12.6	11.8	755
**BnF Froehner verre 13**	amber	bust & inscription	Theodotos	2.18	21.0	541/542 546/547	15.1	1.30	2.76	65.9	0.28	0.66	1.00	7.78	0.17	1.84	3.00	7.98	185	60.7	24.3	25.1	34.4	135	59.9	69.6	16.3	9.20	748
**BnF Froehner verre 26**	yellowish green	cruciform monogram	Theodoros / Dorotheos	4.07	25.0	600-625	19.0	1.09	2.70	65.0	0.17	0.87	0.79	7.26	0.15	0.70	2.02	5.38	170	43.2	20.8	11.0	18.3	52.7	31.6	58.3	12.8	7.55	560
**BnF Froehner verre 29**	yellowish green	cruciform monogram	Timotheos	3.92	24.7	6th - 7th	18.1	1.31	2.65	64.2	0.24	0.91	0.88	7.81	0.15	1.09	2.39	6.09	175	50.3	19.5	17.1	27.9	80.1	43.4	72.6	13.8	8.27	658
**BnF Froehner verre 30**	yellowish green	cruciform monogram	Timotheos	2.10	22.0	6th - 7th	17.4	1.12	2.63	66.0	0.22	0.88	0.81	6.49	0.15	1.09	2.93	5.92	177	72.2	19.7	25.2	28.6	140	41.8	63.2	18.1	8.40	531
**BnF Froehner verre 7**	yellowish green	bust & inscription	Kyrionymos	3.12	23.9	6th - 7th	17.8	1.09	2.48	66.8	0.18	0.86	0.70	6.74	0.15	0.97	2.03	4.73	163	44.4	17.8	14.1	23.6	59.2	114	4.85	12.0	7.15	566
**BnF reg L 3725**	yellowish green	bust & inscription	Theodotos	1.46	19.3	541/542 546/547	17.7	0.92	2.73	67.5	0.12	0.84	0.63	5.15	0.16	1.26	2.75	3.95	193	64.8	19.6	20.9	31.0	65.5	43.0	60.8	12.9	8.96	455
**BnF reg M 4416**	yellowish green	bust & inscription	Kyrionymos	4.48	26.1	6th - 7th	16.8	1.15	2.70	65.8	0.23	0.84	0.89	6.81	0.15	1.29	3.07	7.51	180	80.8	15.8	27.5	32.1	145	44.7	69.0	19.0	9.78	586
**BnF reg N 4260**	yellowish green	bust & inscription	Theodotos	2.18	20.8	6th - 7th	17.8	0.98	2.86	67.2	0.12	0.84	0.66	5.51	0.18	1.09	2.59	6.54	190	62.4	18.7	17.7	28.9	77.4	45.8	59.2	16.0	8.81	464
**BnF reg N 4262**	yellowish green	bust & inscription	Kyrionymos	2.21	20.6	6th - 7th	18.1	1.33	2.71	64.0	0.23	0.85	0.90	8.06	0.16	1.08	2.31	6.68	172	51.6	15.6	17.6	27.9	75.1	41.2	71.2	13.1	8.71	688
**BnF Schlumberger 3979**	yellowish green	cruciform monogram	Theodoros / Dorotheos	4.37	25.2	600-625	16.2	1.36	2.79	65.2	0.28	0.89	0.94	7.54	0.16	1.44	2.92	6.17	179	67.7	18.8	25.6	31.3	100	57.4	5.99	19.3	8.88	672
**BnF Schlumberger 3983**	yellowish green	cruciform monogram	Theodoros / Dorotheos	2.14	21.4	612.00	18.7	1.34	2.81	63.9	0.24	0.96	0.91	7.09	0.15	1.37	2.21	6.62	173	50.2	17.6	19.6	33.3	66.7	46.6	5.51	13.0	9.77	688
**BnF Schlumberger 3994**	yellowish green	bust & inscription	Ioustinos	1.42	17.2	565-578	16.8	1.37	2.83	64.4	0.26	0.93	1.07	7.81	0.16	0.92	3.26	6.96	158	68.6	19.9	16.5	22.3	126	46.1	5.36	20.7	7.85	599
**Egypt I**																													
**BM 1893,1111.86**	blue-green	box monogram	Hadrianos	4.61	24.2	6th	15.4	0.53	2.88	75.2	0.03	1.19	0.44	3.12	0.21	0.03	0.87	3.79	70.2	14.5	14.4	2.65	6.15	19.5	17.5	4.32	1.63	4.88	148
**BM 1983,1108.3**	green	box monogram		3.73	25.6	6th - 7th	17.6	0.81	4.08	70.6	0.10	1.08	0.45	3.09	0.46	0.04	1.64	2.39	71.4	39.1	57.9	4.97	9.99	3.96	26.0	5.62	1.31	6.92	193
**BM 1986,0406.1**	blue-green	box monogram	Hadrianos	1.58	18.3	6th	16.9	0.53	2.97	73.8	0.03	1.36	0.60	2.57	0.21	0.03	0.91	6.56	103	13.7		2.85	6.02	5.50	14.8	4.44	1.77	4.50	141

Sample Number	Y	Zr	Nb	Mo	Ag	Cd	In	Sn	Sb	Cs	Ba	La	Ce	Pr	Nd	Sm	Eu	Gd	Tb	Dy	Ho	Er	Tm	Yb	Lu	Hf	Ta	W	Au	Pb	Bi	Th	U
**BnF Froehner verre 18**	7.61	78.6	2.45	9.55	0.16	0.08	1.05	30.3	119	0.11	339	7.82	13.2	1.71	7.10	1.48	0.38	1.25	0.21	1.23	0.27	0.75	0.11	0.70	0.10	1.89	0.14	0.20	0.01	2547	0.13	1.28	1.21
**BnF Froehner verre 22**	7.45	72.6	2.35	7.19	0.31	0.09	1.14	16.8	139	0.11	318	7.57	12.5	1.64	6.91	1.34	0.36	1.20	0.21	1.20	0.25	0.69	0.09	0.68	0.10	1.76	0.13	0.20	0.04	3537	0.57	1.23	1.07
**BnF Froehner verre 24**	7.01	68.3	2.22	3.77	0.05	0.04	4.82	19.0	138	0.12	304	7.22	12.2	1.56	6.35	1.46	0.34	1.17	0.19	1.11	0.24	0.72	0.10	0.62	0.11	1.65	0.16	0.17	0.01	11982	0.18	1.16	1.10
**BnF Froehner verre 38**	7.95	94.9	2.90	11.5	0.45	1.06	1.07	73.7	93.4	0.10	229	8.34	14.7	1.87	7.53	1.54	0.40	1.27	0.22	1.31	0.27	0.77	0.11	0.75	0.11	2.29	0.16	0.15	0.04	1278	0.32	1.47	1.34
**BnF Froehner verre 39**	7.69	87.4	2.59	2.47	0.44	0.08	0.50	71.5	62.1	0.08	265	7.96	13.4	1.75	7.10	1.42	0.38	1.17	0.22	1.26	0.27	0.73	0.10	0.72	0.10	2.12	0.15	0.16	0.03	868	0.46	1.33	1.37
**BnF Froehner verre 41**	7.23	72.0	2.49	1.67	0.10	0.04	2.58	88.4	205	0.13	195	7.69	13.3	1.68	6.75	1.32	0.36	1.11	0.20	1.21	0.25	0.69	0.09	0.66	0.10	1.81	0.14	0.15		4276	0.09	1.27	0.92
**BnF Schlumberger 3958**	7.39	76.6	2.47	11.2	1.04	0.06	1.22	33.1	110	0.09	302	7.70	12.8	1.70	6.77	1.38	0.35	1.11	0.20	1.19	0.24	0.70	0.10	0.68	0.10	1.90	0.14	0.21	0.06	2582	0.13	1.31	1.15
**BnF Schlumberger 3977**	6.49	67.6	2.03	0.90	0.07	0.01	0.29	5.58	28.3	0.07	149	6.57	11.4	1.47	5.97	1.17	0.32	1.01	0.18	1.05	0.22	0.61	0.09	0.56	0.09	1.68	0.11	0.06		963	0.04	1.10	1.03
**BnF Schlumberger 3982**	6.04	70.8	1.84	3.20	0.39	0.03	0.80	24.4	0.58	0.07	131	6.32	11.0	1.41	5.67	1.13	0.31	0.93	0.17	0.98	0.21	0.55	0.08	0.54	0.08	1.72	0.11	0.05	0.27	3805	0.05	1.05	1.03
**BnF Schlumberger 3984**	7.95	83.8	2.60	4.18	0.73	0.07	0.51	13.4	147	0.09	349	8.41	14.0	1.81	7.32	1.45	0.38	1.23	0.23	1.29	0.27	0.74	0.10	0.69	0.11	2.04	0.15	0.19	0.02	1353	0.10	1.41	1.29
**BnF Schlumberger 3988**	6.31	84.5	2.40	2.27	0.07	0.01	0.57	43.6	0.07	0.07	158	6.68	12.1	1.48	6.11	1.25	0.33	1.12	0.18	1.08	0.22	0.63	0.09	0.61	0.09	2.01	0.14	0.05		2559	0.02	1.19	1.05
**Foy-2 high Fe**																																	
**BM 1874,0311.4**	10.07	84.2	2.62	2.20	0.21	0.04	0.05	7.56	113	0.17	238	10.8	13.5	2.38	10.9	2.29	0.55	2.20	0.31	1.88	0.36	0.99	0.14	1.05	0.14	2.14	0.15	0.28	0.01	103	0.03	1.29	1.29
**BM 1882,0510.34**	12.50	92.2	3.16	2.55	0.37	0.05	0.06	6.84	164	0.22	230	13.9	15.5	3.14	13.6	2.82	0.69	2.64	0.41	2.41	0.47	1.31	0.18	1.21	0.18	2.18	0.18	0.34	0.02	69.0	0.05	1.53	1.21
**BM 1891,0512.11**	11.66	85.6	2.69	2.23	0.21	0.05	0.05	7.29	36.4	0.18	218	13.7	14.7	2.90	12.6	2.58	0.58	2.29	0.35	2.08	0.41	1.22	0.15	1.12	0.15	1.89	0.14	0.27	0.01	61.7	0.05	1.38	1.23
**BM 1923,1107.1**	10.35	90.5	2.74	2.23	0.22	0.05	0.04	3.66	37.0	0.18	211	11.3	13.7	2.52	11.1	2.18	0.57	2.12	0.33	1.91	0.37	1.10	0.14	0.99	0.15	2.17	0.15	0.24	0.01	43.8	0.05	1.40	1.14
**BM 1980,0611.33**	10.04	86.1	2.74	2.15	0.13	0.10	0.04	3.93	90.2	0.16	211	11.0	13.7	2.48	10.6	2.26	0.54	1.93	0.32	1.78	0.36	1.06	0.14	0.99	0.13	2.09	0.16	0.23		64.9	0.04	1.39	1.24
**BM 1980,0611.48**	11.08	98.3	2.78	2.38	0.13	0.02	0.02	0.69	1.30	0.15	195	12.5	14.8	2.68	11.6	2.31	0.58	2.09	0.33	2.08	0.40	1.14	0.15	1.07	0.14	2.30	0.15	0.23	0.01	3.82	0.04	1.40	1.20
**BM 1980,0611.55**	11.39	111.6	3.19	2.74	0.16	0.04	0.04	5.16	140	0.26	597	11.9	14.6	2.62	11.5	2.36	0.59	2.27	0.36	2.06	0.40	1.13	0.15	1.14	0.17	2.63	0.18	0.91		78.0	0.06	1.50	1.18
**BM 1980,0611.75**	11.11	93.9	3.15	1.79	0.38	0.04	0.06	8.93	175	0.17	193	12.4	15.2	2.73	12.0	2.38	0.60	2.37	0.36	2.03	0.41	1.14	0.15	1.07	0.15	2.26	0.18	0.97	0.02	99.9	0.05	1.50	1.35
**BM 1986,0406.12**	12.15	97.3	3.17	3.59	0.17	0.06	0.16	13.5	72.9	0.21	242	13.5	16.2	2.96	13.1	2.67	0.64	2.49	0.38	2.24	0.46	1.26	0.17	1.19	0.18	2.37	0.19	0.34	0.02	681	0.06	1.60	1.30
**BM 1986,0406.13**	11.83	100.8	3.09	1.30	0.11	0.04	0.04	4.20	143	0.16	189	13.1	14.9	2.86	12.3	2.52	0.58	2.27	0.35	2.14	0.42	1.17	0.16	1.09	0.15	2.33	0.16	0.30	0.01	37.0	0.04	1.45	1.11
**BM 1987,0703.8**	11.12	83.4	2.57	2.33	0.16	0.02	0.07	5.86	37.8	0.18	213	12.0	12.9	2.67	11.6	2.45	0.61	2.28	0.34	2.11	0.42	1.12	0.15	1.13	0.14	1.97	0.15	0.28		198	0.05	1.27	1.12
**BM 1987,0703.9**	10.56	88.8	2.76	2.11	0.11	0.02	0.03	4.15	37.7	0.17	213	11.7	14.0	2.59	11.2	2.31	0.58	2.04	0.32	1.93	0.39	1.07	0.14	1.01	0.15	2.09	0.16	0.25		59.0	0.04	1.40	1.16
**BM 1987,0703.10**	10.39	79.1	2.39	3.76	0.29	0.05	0.04	5.66	23.9	0.21	293	11.4	13.6	2.46	11.0	2.36	0.55	2.23	0.32	1.94	0.38	1.05	0.14	1.03	0.14	1.89	0.13	0.33	0.06	53.6	0.08	1.31	1.29
**BM 1987,0703.13**	9.95	82.7	2.62	2.05	0.15	0.03	0.04	6.30	127	0.17	216	10.8	13.3	2.45	10.9	2.21	0.54	2.21	0.32	1.95	0.38	1.06	0.15	0.99	0.13	1.99	0.16	0.36		72.8	0.03	1.35	1.27
**BM 1990,0601.16**	11.06	79.5	2.82	2.02	0.41	0.07	0.04	5.88	201	0.35	316	11.5	16.3	2.71	11.6	2.45	0.60	2.38	0.35	1.98	0.39	1.08	0.15	1.06	0.15	1.93	0.16	0.54		76.3	0.09	1.48	1.35
**BM 1990,0601.19**	11.35	91.5	2.71	2.18	0.15	0.05	0.05	5.93	28.9	0.16	225	12.1	13.8	2.75	12.1	2.47	0.60	2.29	0.36	2.12	0.40	1.12	0.16	1.05	0.15	2.09	0.15	0.28		68.2	0.05	1.32	1.34
**BM 1990,0601.20**	10.22	84.5	2.62	2.39	0.15	0.03	0.05	6.35	120	0.19	241	11.5	14.0	2.54	10.9	2.16	0.53	1.99	0.30	1.82	0.38	1.04	0.14	0.98	0.14	1.98	0.14	0.28	0.01	96.3	0.04	1.34	1.30
**BnF AA de Clercq 7**	14.29	93.1	3.27	2.52	0.41	0.07	0.05	3.73	244	0.50	359	15.7	21.1	3.29	14.1	2.83	0.71	2.56	0.43	2.56	0.51	1.38	0.20	1.31	0.19	2.17	0.19	0.62		94.7	0.12	1.93	1.33
**BnF AA VA 17**	13.48	96.1	3.25	2.15	0.25	0.06	0.06	9.61	267	0.35	370	15.0	20.3	3.22	13.3	2.74	0.71	2.38	0.39	2.37	0.49	1.31	0.18	1.21	0.17	2.28	0.19	0.55	0.02	134	0.12	1.93	1.50
**BnF Froehner verre 13**	11.98	91.9	2.97	4.52	0.29	0.06	0.07	7.82	31.7	0.14	234	13.1	14.7	2.77	11.7	2.36	0.59	2.12	0.37	2.10	0.45	1.16	0.16	1.17	0.16	2.12	0.17	0.34		109	0.04	1.45	1.15
**BnF Froehner verre 26**	10.02	89.4	2.78	1.63	0.12	0.07	0.03	4.55	153	0.15	199	11.1	14.0	2.41	10.1	2.08	0.52	1.86	0.30	1.80	0.37	0.98	0.14	0.92	0.13	2.14	0.16	0.62		54.1	0.04	1.45	1.40
**BnF Froehner verre 29**	10.79	85.8	2.71	2.46	0.14	0.05	0.06	6.94	127	0.17	249	12.1	14.5	2.64	10.9	2.15	0.55	1.94	0.34	1.93	0.41	1.05	0.15	0.99	0.15	2.08	0.15	0.32	0.01	109	0.05	1.40	1.34
**BnF Froehner verre 30**	11.47	85.5	2.70	2.52	0.10	0.05	0.07	5.74	37.8	0.18	215	13.0	14.0	2.82	11.5	2.28	0.60	2.12	0.36	2.04	0.42	1.15	0.15	1.05	0.16	1.92	0.14	0.30	0.01	203	0.05	1.35	1.21
**BnF Froehner verre 7**	10.60	92.8	2.75	2.31	0.20	0.08	0.04	3.89	118	0.14	223	11.9	14.3	2.54	10.4	2.09	0.54	1.82	0.31	1.82	0.39	1.08	0.14	0.98	0.14	2.21	0.16	0.45	0.01	42.2	0.06	1.46	1.39
**BnF reg L 3725**	11.75	94.7	2.79	2.96	0.24	0.04	0.02	0.88	3.11	0.14	201	12.7	13.9	2.80	11.5	2.32	0.59	2.09	0.36	2.00	0.42	1.20	0.16	1.09	0.16	2.20	0.14	0.31	0.01	15.0	0.02	1.35	1.13
**BnF reg M 4416**	12.29	87.5	2.77	2.61	0.12	0.09	0.07	5.66	36.4	0.19	240	13.4	13.9	2.95	12.2	2.52	0.61	2.25	0.38	2.18	0.45	1.18	0.17	1.10	0.17	2.05	0.15	0.31		199	0.04	1.35	1.19
**BnF reg N 4260**	12.07	101.9	3.36	2.35	2.20	0.08	0.03	1.73		0.16	205	13.2	14.9	2.88	11.9	2.47	0.62	2.16	0.37	2.21	0.44	1.20	0.18	1.14	0.17	2.41	0.19	0.27	0.10	27.3	0.07	1.49	1.23
**BnF reg N 4262**	10.98	90.6	2.92	2.42	12.9	0.06	0.06	8.15	131	0.18	255	12.4	15.0	2.70	11.1	2.26	0.59	1.92	0.34	1.97	0.41	1.11	0.15	1.06	0.15	2.17	0.16	0.32	0.13	127	0.05	1.45	1.35
**BnF Schlumberger 3979**	13.08	97.6	3.04	3.69	0.42	0.05	0.06	5.68	25.7	0.19	243	14.8	16.6	3.17	13.1	2.69	0.67	2.22	0.39	2.36	0.48	1.33	0.19	1.20	0.18	2.35	0.17	0.31	0.01	311	0.07	1.67	1.33
**BnF Schlumberger 3983**	12.01	81.4	2.92	3.83	0.31	0.05	0.06	5.23	162	0.29	357	14.0	16.9	2.95	12.1	2.40	0.64	2.01	0.36	2.12	0.42	1.17	0.17	1.09	0.16	1.97	0.17	0.64	0.03	32.6	0.07	1.62	1.45
**BnF Schlumberger 3994**	12.50	90.4	2.93	1.27	0.27	0.05	0.06	7.55	133	0.15	197	14.0	15.2	3.04	12.6	2.69	0.66	2.49	0.39	2.22	0.47	1.28	0.19	1.22	0.18	2.10	0.17	0.22		109	0.05	1.47	1.17
**Egypt I**																																	
**BM 1893,1111.86**	6.77	98.6	2.89	0.12	0.33	0.08	0.01	0.78	0.49	0.04	159	7.05	13.7	1.67	7.01	1.44	0.40	1.28	0.20	1.24	0.23	0.70	0.10	0.72	0.10	2.25	0.16	0.05	0.01	8.04	0.01	1.22	0.69
**BM 1983,1108.3**	8.14	149.1	4.22	0.07	0.10	0.05	0.01	0.49		0.06	186	8.01	15.9	2.00	8.64	1.86	0.50	1.59	0.26	1.49	0.31	0.83	0.12	0.87	0.12	3.54	0.23	0.07		0.86		1.53	0.78
**BM 1986,0406.1**	7.12	99.9	2.83	0.15	0.09		0.01	0.83	0.40	0.05	167	7.55	14.7	1.74	7.59	1.56	0.38	1.35	0.21	1.26	0.26	0.72	0.10	0.72	0.11	2.43	0.17	0.03	0.02	7.33	0.01	1.29	0.82

S1 Table: LA-ICP-MS data of the Byzantine glass weights. Major and minor oxides [wt%], including chlorine, and trace elements [ppm]; where the monograms are ambiguous, multiple names are given (Tobias, forthcoming).

Sample Number	colour	type	names	weight [g]	Ø [mm]	Date	Na_2O	MgO	Al_2O_3	SiO_2	P_2O_5	Cl	K_2O	CaO	TiO_2	MnO	Fe_2O_3	Li	B	V	Cr	Co	Ni	Cu	Zn	Ga	As	Rb	Sr	Y	Zr	Nb	Mo	Ag	Cd	In	Sn	Sb	Cs	Ba	La	Ce	Pr	Nd	Sm	Eu	Gd	Tb	Dy	Ho	Er	Tm	Yb	Lu	Hf	Ta	W	Au	Pb	Bi	Th	U
**BM 1986,0406.2**	blue-green	box monogram	Hadrianos	3.96	23.6	6th	13.4	0.53	3.01	77.6	0.04	1.09	0.52	2.72	0.19	0.03	0.86	3.56	59.7	14.3	9.92	2.67	5.27	6.36	13.4	4.53	1.83	3.89	144	6.88	87.2	2.59	0.07	0.13		0.00	0.66	1.05	0.03	168	7.03	13.6	1.70	7.07	1.56	0.37	1.36	0.22	1.20	0.26	0.73	0.09	0.77	0.09	2.16	0.17	0.04	0.01	3.74	0.01	1.12	0.56
**BM 1990,0601.15**	purple	cruciform monogram	Agrestios	2.35	20.7	6th - 7th	17.0	0.62	3.53	69.9	0.10	1.01	1.06	3.62	0.25	1.61	1.13	5.11	61.7	41.8	22.2	42.7	25.0	313	50.9	5.68	3.76	8.99	285	7.33	91.0	2.85	4.23	0.23	0.12	0.09	21.1	8.08	0.10	746	7.04	14.0	1.67	7.53	1.55	0.45	1.62	0.21	1.30	0.26	0.78	0.10	0.80	0.12	2.09	0.17	0.49	0.01	99.4	0.05	1.14	0.94
**BnF AA VA 19**	purple	cruciform monogram	Agrestios	2.30	18.8	6th - 7th	16.7	0.62	3.46	71.0	0.10	0.97	0.70	3.40	0.25	1.45	1.08	4.79	58.9	39.2	39.2	35.6	20.9	193	42.9	212	3.64	9.72	301	7.54	93.5	3.06	3.51	0.31	0.10	0.11	25.5	8.52	0.10	775	7.66	15.1	1.75	7.32	1.47	0.43	1.27	0.21	1.29	0.27	0.71	0.11	0.76	0.11	2.25	0.17	0.39	0.01	123	0.05	1.20	0.92
**BnF Froehner verre 34**	aqua / bluish	cruciform monogram	Belliados	4.49	26.5	6th - 7th	17.1	0.69	4.10	72.4	0.05	0.97	0.64	1.85	0.32	0.03	1.79	3.27	51.9	31.2	39.2	7.52	13.5	10.1	17.4	5.83	0.94	7.84	149	8.17	98.8	3.99	0.08	0.04	0.04	0.01	0.15		0.08	224	9.50	18.9	2.19	9.28	1.87	0.53	1.65	0.25	1.49	0.30	0.79	0.12	0.77	0.12	2.39	0.23	0.05		2.31	0.01	1.40	0.57
**HIMT / HIT**																																																														
**BM 1980,0611.15**	cobalt blue	box monogram	Dikoros / Dioskoros	1.42	17.2	6th.	18.1	1.04	3.05	65.3	0.05	1.05	0.44	5.24	0.30	0.13	3.64	4.85	148	29.4	37.7	4155	135	4109	133	11.2	12.7	6.71	373	7.26	136.8	3.42	15.6	0.26	0.06	2.39	40.1	3.13	0.10	142	6.80	12.4	1.58	7.00	1.51	0.37	1.40	0.22	1.23	0.25	0.70	0.10	0.76	0.11	3.26	0.21	0.09		3531	0.08	1.36	0.86
**BM 1986,0406.4**	olive green	cruciform monogram	Ioulianos	2.19	19.8	6th - 7th	18.3	1.36	3.53	62.8	0.07	0.97	0.61	7.34	0.72	1.59	2.40	7.51	164	51.1	83.0	13.5	16.8	213	44.5	6.65	6.94	8.11	561	11.85	341.8	7.12	2.31	34.17	0.37	0.12	25.1	2.22	0.80	454	11.4	22.2	2.70	11.7	2.41	0.57	2.24	0.35	2.08	0.42	1.27	0.18	1.34	0.20	7.71	0.41	0.51	1.75	80.5	0.23	2.83	1.30
**BM OA.4049**	olive green	Latin monogram	possibly vessel bottom	2.13	21.6	6th	18.6	1.10	2.51	65.7	0.06	1.05	0.37	6.63	0.38	1.86	1.53	4.76	198	38.3	45.8	6.95	16.9	39.7	27.5	4.73	4.77	4.98	492	9.32	203.7	4.16	3.37	0.30	0.07	0.02	2.65	2.77	0.06	222	9.41	14.2	2.12	9.20	1.88	0.48	1.87	0.28	1.64	0.33	0.98	0.14	0.98	0.14	4.70	0.24	0.21	0.01	31.7	0.08	1.67	1.19
**High Mg**																																																														
**BM 1872,0320.13**	blue-green	cruciform monogram	Kyros	4.56	21.0	6th - 7th	15.9	1.67	1.70	68.7	0.28	0.96	1.36	8.24	0.15	0.09	0.80	3.80	156	16.6	12.2	2.82	5.08	33.7	23.3	2.90	1.86	5.18	555	5.28	81.5	2.23	0.37	2.57	0.07	0.02	2.79		0.03	132	5.12	9.27	1.15	4.97	1.05	0.23	0.99	0.15	0.88	0.17	0.52	0.08	0.55	0.07	2.02	0.14	0.03	0.12	32.9	0.01	1.10	1.50
**BM 1892,0613.65**	cobalt blue	cruciform monogram	Andreas / Rena	1.23	19.1	6th - 7th	15.2	1.82	1.99	66.4	0.43	0.68	1.76	9.56	0.17	0.22	1.27	6.55	143	20.4	21.0	429	99.8	832	37.1	4.13	8.99	7.00	723	5.92	88.2	2.59	2.18	0.39	0.05	0.47	26.1	42.2	0.09	193	5.99	10.9	1.39	6.10	1.24	0.27	1.16	0.17	1.01	0.20	0.60	0.08	0.62	0.09	2.13	0.16	0.11	0.03	1958	0.05	1.22	0.96
**BM 1980,0611.34**	blue-green	cruciform monogram	Gregorios	2.03	20.0	6th - 7th	14.2	2.12	2.47	67.1	0.46	0.66	1.84	9.39	0.19	0.16	1.16	7.86	152	21.2	23.6	5.12	10.4	53.3	23.0	3.71	3.18	9.53	623	6.14	89.2	2.75	0.64	2.13	0.03	0.02	4.11	32.3	0.26	200	6.26	11.8	1.48	6.58	1.34	0.32	1.31	0.18	1.11	0.22	0.63	0.09	0.72	0.09	2.16	0.18	0.16	0.07	75.6	0.06	1.46	1.05
**BM 1980,0611.47**	blue-green	bust & inscription	Hesychios	3.73	25.4	6th - 7th	16.0	1.22	2.33	67.0	0.24	0.79	1.97	8.69	0.15	0.32	1.03	8.13	131	20.8	19.5	12.3	11.0	53.4	28.1	3.71	5.73	7.82	633	6.59	81.7	2.37	0.85	0.12	0.07	0.04	6.72	63.0	0.12	212	6.38	11.0	1.51	6.50	1.32	0.35	1.30	0.18	1.08	0.23	0.65	0.09	0.67	0.09	2.04	0.14	0.21	0.01	131	0.02	1.14	1.25
**BM 1987,0703.22**	blue-green	mark s		0.56	14.0	6th - 7th	18.2	1.39	2.00	67.3	0.28	0.89	0.99	7.41	0.22	0.12	1.04	5.74	159	19.9	15.3	4.38	6.61	22.1	16.3	3.41	4.42	5.47	502	6.35	119.6	3.06	0.55	0.32	0.03	0.02	2.78	84.7	0.09	152	6.72	12.6	1.51	6.43	1.27	0.27	1.11	0.18	1.13	0.22	0.62	0.10	0.67	0.11	2.87	0.18	0.08	0.02	52.8	0.02	1.50	1.18
**BnF AA de Clercq 4**	amber tinge	box monogram	Dikoros / Dioskoros	0.92	17.3	6th	13.0	2.62	2.10	66.3	0.49	0.89	2.52	9.19	0.13	1.14	0.77	6.79	96.0	17.6	19.6	10.9	11.6	298	98.4	4.15	6.18	14.3	548	6.96	56.1	2.32	3.33	1.99	0.07	0.30	72.4	37.5	0.17	248	7.22	13.1	1.59	6.73	1.38	0.38	1.25	0.19	1.12	0.23	0.60	0.09	0.60	0.08	1.35	0.12	0.42	0.24	5559	0.25	1.12	0.60
**BnF AA VA 20**	aqua bluish	cruciform monogram		1.68	22.0	6th - 7th	18.4	1.27	1.84	66.9	0.25	1.02	1.20	7.94	0.17	0.08	0.83	4.95	152	14.9	20.1	2.96	5.36	10.9	25.7	41.5	1.81	4.87	540	5.74	80.3	2.58	0.37	0.09	0.03	0.01	1.41	5.07	0.07	148	6.03	11.2	1.38	5.46	1.10	0.27	0.92	0.16	0.98	0.20	0.56	0.08	0.56	0.09	1.93	0.15	0.07		25.7	0.01	1.23	0.78
**BnF Froehner verre 05**	amber	bust & inscription	Ioannos	4.51	25.0	6th - 7th	15.8	2.07	2.20	62.9	0.44	0.83	1.36	9.46	0.19	2.40	2.05	6.85	140	60.5	24.2	19.1	39.1	66.8	62.1	5.04	9.54	6.62	892	10.69	93.3	2.79	4.88	0.08	0.08	0.03	2.05	30.0	0.15	553	11.6	14.0	2.32	9.71	1.94	0.51	1.87	0.31	1.89	0.38	1.05	0.16	1.03	0.15	2.14	0.15	0.55	0.01	34.9	0.04	1.42	0.92
**BnF Froehner verre 06**	green	bust & inscription	Ioannos	4.34	25.4	6th - 7th	17.8	1.82	1.84	65.6	0.40	1.11	1.44	8.59	0.14	0.11	0.91	2.84	166	15.5	16.7	3.17	5.61	13.9	13.6	50.7		5.24	565	5.34	70.7	2.37	0.59	0.45	0.03	0.01	2.34	0.74	0.05	171	5.48	10.0	1.23	5.03	1.00	0.24	0.88	0.14	0.85	0.18	0.51	0.08	0.50	0.08	1.71	0.13	0.07		24.8		1.08	0.72
**BnF Froehner verre 10**	aqua	bust & inscription	Ioannos	2.05	20.0	6th - 7th	16.2	1.51	1.50	69.6	0.31	0.93	1.31	7.58	0.16	0.07	0.72	4.15	136	14.1	17.2	2.42	4.46	75.7	28.4	42.9		4.27	486	4.97	62.9	2.19	0.37	15.42	0.04	0.04	8.13		0.01	144	5.01	9.40	1.15	4.75	1.03	0.21	0.78	0.13	0.81	0.17	0.50	0.07	0.51	0.07	1.50	0.11	0.04	0.15	15.0	0.02	1.00	0.69
**BnF Froehner verre 19**	aqua	bust & inscription	Neilos	2.15	21.0	6th - 7th	18.2	1.80	1.96	66.0	0.34	0.65	1.50	8.10	0.19	0.13	0.95	4.90	153	19.7	24.8	4.69	6.63	16.8	16.8	51.8	2.75	6.08	553	5.73	105.9	2.86	0.58	0.11	0.04	0.02	1.84	11.3	0.08	179	6.10	11.2	1.38	5.55	1.13	0.26	0.97	0.16	0.96	0.20	0.57	0.08	0.58	0.09	2.51	0.16	0.06	0.01	21.7	0.01	1.28	1.24
**BnF Froehner verre 20**	amber	bust & inscription	Neilos	1.51	19.0	6th - 7th	14.9	1.68	2.08	66.3	0.32	0.65	1.33	7.97	0.18	2.24	2.14	5.53	138	61.1	23.7	21.4	50.3	74.2	64.3	115	11.7	8.48	835	10.88	98.2	2.92	6.42	0.28	0.10	0.04	3.51	23.5	0.22	402	12.6	13.9	2.57	10.6	2.21	0.53	1.90	0.34	1.93	0.41	1.09	0.15	1.05	0.15	2.29	0.15	0.57	0.01	37.9	0.05	1.42	1.18
**BnF Froehner verre 27**	aqua	cruciform monogram	Theognios	3.43	26.7	6th - 7th	18.0	1.43	2.24	66.5	0.30	0.88	1.09	7.86	0.22	0.14	1.14	4.75	158	21.8	26.9	6.08	7.59	28.1	21.2	46.8	3.54	6.21	567	6.79	118.7	3.38	0.53	0.43	0.05	0.03	4.17	169	0.09	165	7.28	13.6	1.65	6.57	1.37	0.32	1.13	0.20	1.13	0.24	0.67	0.10	0.69	0.11	2.77	0.19	0.08		65.1	0.02	1.53	1.22
**BnF Froehner verre 28**	aqua	cruciform monogram	Gregorios	3.53	24.5	6th - 7th	14.5	2.04	1.78	67.7	0.46	0.64	1.80	9.50	0.18	0.16	1.00	5.06	144	19.7	21.5	4.79	6.23	20.3	20.3	56.5	2.59	7.05	649	5.52	92.4	2.75	0.71	0.89	0.02	0.02	4.49	37.1	0.10	207	6.07	11.6	1.36	5.48	1.12	0.26	0.91	0.16	0.96	0.20	0.56	0.08	0.60	0.09	2.23	0.16	0.08	0.02	36.5	0.01	1.25	1.13
**BnF reg L 4097**	aqua bluish	cruciform monogram	Staurakios	2.00	20.0	End 6th / Beginning 7th	16.6	2.35	2.16	65.3	0.40	0.53	1.61	9.24	0.16	0.31	1.17	5.37	196	24.1	18.1	9.93	11.4	20.2	20.3	59.2	0.72	5.97	649	6.18	84.0	2.77	1.17	0.12	0.00	0.01	1.76	15.8	0.05	205	6.64	11.5	1.52	6.18	1.20	0.31	1.06	0.17	1.07	0.22	0.61	0.09	0.59	0.09	2.02	0.16	0.08	0.01	18.3		1.27	1.45
**BnF reg M 2903**	aqua bluish	bust & inscription	Hesychios	1.50	19.3	602-610	17.0	1.95	1.97	66.0	0.37	0.60	1.63	8.94	0.19	0.11	0.99	5.98	156	20.3	22.1	3.47	6.24	9.63	17.3	53.1	1.76	6.58	599	5.84	104.3	2.92	0.52	0.16	0.03	0.01	1.15	7.09	0.09	191	6.21	11.8	1.43	5.77	1.15	0.29	0.95	0.16	0.98	0.21	0.60	0.08	0.60	0.09	2.47	0.17	0.08	0.01	9.67	0.01	1.32	1.24
**BnF reg N 4261**	aqua bluish	bust & inscription	Hesychios	2.05	21.4	602-610	15.8	2.05	2.47	64.7	0.43	0.57	1.60	10.3	0.23	0.27	1.34	8.36	147	25.1	25.8	7.45	11.4	45.5	34.3	58.7	3.31	7.58	748	7.66	131.9	3.71	0.98	0.19	0.06	0.06	13.5	39.3	0.11	204	8.03	14.2	1.80	7.30	1.49	0.35	1.27	0.22	1.33	0.27	0.75	0.12	0.77	0.12	3.13	0.21	0.17	0.01	213	0.03	1.71	1.30
**BnF Schlumberger 3980**	yellowish green	box monogram	Theodoros / Dorotheos	3.71	26.7	600-625	13.3	2.01	1.73	68.7	0.78	0.57	2.62	8.42	0.16	0.34	1.19	4.48	154	18.2	19.1	5.48	7.58	28.4	25.1	3.31	1.91	6.51	662	5.68	77.8	2.78	1.63	0.08		0.02	3.28	29.0	0.05	203	5.90	10.9	1.31	5.27	1.16	0.27	0.88	0.16	0.93	0.20	0.55	0.08	0.57	0.08	1.93	0.15	0.12	0.01	37.6	0.01	1.23	0.90
**Large Weights**																																																														
**BM 2002 1001 1 (Lev I)**	green	denominal mark	Gamma & Alpha	26.48	32.5x32.5	< 540 CE	12.4	0.45	2.73	73.3	0.00	1.24	0.49	8.33	0.05	0.04	0.35	26.5	105	6.15	26.0	2.82	6.03	7.96	8.72	2.99	2.63	7.59	396	5.49	30.3	1.04	0.45	0.11	0.17	0.05	10.8	2.07	0.11	198	4.83	9.06	1.10	4.93	0.96	0.34	1.03	0.16	0.87	0.18	0.50	0.06	0.45	0.05	0.71	0.05	0.05	0.02	25.6		0.58	0.41
**BM 1946 0406 2 (Foy-2)**	green	box monogram		80.23	50.5x54.1	650 - 700	17.5	0.83	2.20	66.5	0.00	0.92	1.03	8.18	0.13	1.36	0.91	14.5	172	28.8	35.1	8.29	10.1	56.3	26.2	3.25	5.42	7.97	634	6.67	70.3	2.07	2.35	0.39	0.09	0.07	18.6	130	0.08	315	6.60	10.9	1.39	6.23	1.32	0.35	1.22	0.18	1.14	0.23	0.63	0.08	0.62	0.09	1.59	0.13	0.14	0.01	73.3	0.03	1.08	1.10
**BM 1980 0611 1 (Foy-2)**	yellowish / olive green	bust & box monogram	Mauritius Tiberius	74.59	58.8	6th	18.1	0.75	2.05	66.8	0.01	0.65	0.54	8.49	0.10	1.50	0.68	6.95	187	28.8	34.6	5.84	8.18	33.0	17.3	2.83	4.12	6.19	632	6.36	60.4	1.75	1.93	0.20	0.10	0.06	14.0	110	0.04	355	6.49	10.7	1.38	6.44	1.20	0.33	1.23	0.18	1.14	0.23	0.63	0.08	0.65	0.09	1.50	0.10	0.09		29.8	0.01	1.02	1.37
**BM 1982 1003 6 (Foy-2)**	variegated green	box monogram		82.96	61.2	650 - 700	17.5	1.13	2.57	65.0	0.14	0.84	1.04	8.74	0.15	1.51	1.10	9.10	162	29.9	21.0	9.06	17.2	64.9	34.3	4.64	6.32	7.86	719	7.24	76.7	2.53	4.03	0.27	0.07	0.04	8.83	161	0.12	282	7.50	12.8	1.69	7.54	1.54	0.39	1.50	0.22	1.30	0.27	0.73	0.10	0.75	0.10	2.00	0.15	0.17	0.01	135	0.06	1.21	1.09
**BM 1986 0602 1 (Foy-2)**	green	5 box monograms		314.00	103.7	6th	15.0	0.68	2.61	71.4	0.00	0.94	0.82	7.46	0.11	0.18	0.55	10.9	144	12.9	47.5	2.25	6.78	9.67	10.8	3.00	2.34	11.7	368	6.13	70.1	1.71	1.03	0.21	0.12	0.05	13.9	1.48	0.10	184	5.88	10.9	1.31	5.92	1.18	0.33	1.02	0.17	1.03	0.22	0.62	0.07	0.59	0.09	1.63	0.10	0.09		13.9		0.99	0.72
**BM 1990 0601 10 (Foy-2)**	green	box monogram		97.27	79.5	6th - 7th	19.8	1.03	2.23	64.0	0.09	0.63	0.60	9.07	0.12	1.48	0.76	4.87	210	32.3	19.7	6.33	7.99	22.0	15.4	3.22	4.00	6.29	757	6.70	70.1	2.18	2.51	0.18	0.11	0.06	15.5	73.3	0.06	389	6.73	11.4	1.46	6.63	1.26	0.32	1.23	0.19	1.14	0.23	0.69	0.08	0.65	0.10	1.71	0.12	0.11		20.9	0.01	1.22	1.50
**BM 1990 0601 11 (Foy-2)**	green	box monogram		12.92	30.0	6th	19.5	1.12	2.60	64.0	0.13	0.76	0.60	9.41	0.14	0.66	0.86	5.34	179	24.1	19.5	4.28	9.50	14.6	17.3	3.27	2.81	7.27	800	7.16	84.8	2.56	1.56	0.39		0.05	13.0	14.2	0.07	258	7.35	12.8	1.60	7.18	1.46	0.37	1.33	0.20	1.27	0.27	0.71	0.12	0.77	0.10	2.08	0.16	0.12		15.3	0.02	1.43	1.40
**BM 1946 0406 1 (HIMT)**	green	cross with letters / symbols		146.27	67x66.2	650 - 700	17.2	1.11	3.44	64.5	0.20	0.73	1.23	7.32	0.30	1.53	2.22	5.82	144	59.7	41.9	12.7	26.5	88.2	237	5.70	9.91	9.90	562	9.74	123.1	3.64	4.17	0.71	0.08	0.05	7.54	86.4	0.12	286	9.45	14.2	2.19	9.81	2.08	0.53	2.28	0.32	1.73	0.35	1.02	0.14	1.01	0.14	2.90	0.21	0.31	0.02	109	0.07	1.41	1.09
**Outliers**																																																														
**BM 1980,0611.35**	prussian blue	cruciform monogram	Theognios	2.29	19.5	6th - 7th	10.0	0.64	3.47	68.1	0.12	1.04	5.95	8.99	0.08	0.27	0.78	7.86	67.7	12.9	17.4	270	91.9	267	25.3	4.89	8.14	178	491	7.06	37.2	1.42	1.53	1.99	0.05	0.87	183	2.19	3.16	282	6.39	11.4	1.48	6.78	1.40	0.41	1.46	0.21	1.24	0.24	0.68	0.10	0.65	0.09	0.97	0.09	1.88	0.11	2903	0.31	0.77	0.55
**BM 1980,0611.40**	cobalt blue	cruciform monogram	Megethios	1.48	19.6	6th - 7th	13.8	0.73	3.45	63.6	0.09	0.79	4.25	11.0	0.09	0.35	0.98	4.58	92.1	13.1	9.20	726	223	1050	28.8	8.13	27.5	12.0	575	8.23	47.0	1.59	5.06	0.12	0.09	1.27	67.0	6.96	0.10	250	7.12	13.4	1.68	7.63	1.59	0.42	1.40	0.22	1.32	0.26	0.73	0.10	0.71	0.10	1.23	0.10	0.12	0.01	4817	0.04	0.92	1.02
**BM 1980,1204.2**	green	H	mark H	1.47	14.9	6th - 7th	0.1	0.05	0.21	26.3	0.03	0.14	0.03	0.32	0.03	0.00	0.17	1.78	0.85	1.9		0.42	6.00	3138	5.01	0.50	216	0.91	8	0.89	7.2	0.66	0.06	153	0.33	6.86	1867	942	0.04	14	1.00	1.88	0.23	1.08	0.26	0.04	0.21	0.03	0.17	0.03	0.10	0.01	0.09	0.01	0.18	0.04	0.02	1.00	667288	316.68	0.14	0.04
**BM 1981,0601.6**	green	bust		4.10	24.3	6th - 7th	11.0	3.61	1.63	69.2	0.27	0.84	3.65	8.57	0.13	0.30	0.71	15.1	102	14.6	62.0	3.07	23.0	10.5	25.2	2.70	2.40	9.41	381	4.11	115.5	2.06	1.73	0.32	0.09	0.00	0.54	0.11	0.12	102	4.12	7.56	0.93	3.67	0.78	0.17	0.75	0.12	0.71	0.14	0.44	0.07	0.46	0.07	2.79	0.14	0.05		1.68	0.02	1.43	0.63
**BM 1983,1108.4**	green	box monogram		1.50	15.2	650 - 700	14.4	0.52	2.49	70.3	0.07	1.16	0.34	9.46	0.26	0.02	0.92	4.04	84.3	19.7	24.6	2.74	6.32	6.79	15.0	3.80	2.10	4.82	166	6.06	179.2	3.30	0.08	0.25	0.02	0.01	0.53		0.06	153	6.18	11.7	1.49	6.37	1.31	0.32	1.18	0.18	1.02	0.21	0.60	0.10	0.72	0.10	4.28	0.20	0.05		48.3	0.01	1.37	0.86
**BM 1986,0406.20**	green	III		3.08	26.2		11.8	3.29	1.49	67.3	0.37	0.70	2.50	10.5	0.10	0.94	0.77	13.9	106	10.7	2.73	15.0	9.80	235	77.6	3.08	11.6	21.2	571	5.10	116.8	2.09	1.12	0.37	0.05	0.76	50.6	11.2	0.40	314	7.19	12.8	1.52	6.24	1.24	0.24	1.07	0.15	0.87	0.17	0.50	0.07	0.53	0.07	2.63	0.14	0.59	0.06	141	0.04	1.62	0.64
**BnF Froehner verre 44**	greenish	cross & dots in the corners		4.40	22.5		16.0	0.19	9.06	67.2	0.12	0.91	2.60	2.60	0.14	0.01	0.69	13.7	735	20.4	10.4	2.11	5.25	31.3	29.5	249	133	61.4	239	7.41	69.2	3.60	1.66	4.46	0.09	0.06	14.1	0.18	3.12	949	34.2	56.6	5.45	18.1	2.73	0.59	1.84	0.26	1.32	0.26	0.70	0.10	0.70	0.10	1.78	0.37	0.68	0.59	39.7	0.17	12.14	4.64
**BnF Schlumberger 3997**	turquoise	letters		1.04	16.8		15.4	0.47	2.21	68.9	0.06	0.77	0.52	7.16	0.05	0.05	0.47	3.18	102	6.40	11.2	2.04	14.6	10179	12.2	2.85	7.78	7.85	360	6.01	36.3	1.06	0.30	4.27	0.03	2.41	611	112	0.07	169	5.52	9.51	1.21	5.30	1.08	0.29	1.02	0.16	0.92	0.20	0.54	0.08	0.51	0.07	0.93	0.06	0.02	0.10	22974	0.56	0.70	0.79
**Modern**																																																														
**BM 1980,0611.60**	amber	2 standing figures	Phocas and Leontia	3.48	30.5		12.6	3.02	2.16	71.8	0.02	0.06	1.08	6.56	0.08	0.01	0.21	9.08	13.1	7.11		1.11	13.3	31.0	8228	4.16	3.24	35.4	215	6.66	172.9	1.86	0.21	0.16	0.02	0.31	9.99	1.14	0.80	11547	11.7	21.8	2.31	8.66	1.38	0.47	1.46	0.15	0.87	0.17	0.48	0.07	0.54	0.08	4.01	0.12	0.26		10.4		4.42	0.70
**BM 1980,1204.3°**	pink			3.58	20.2		16.3	0.07	0.26	71.0	0.03	0.11	0.05	10.3	0.03	0.17	0.25	9.27	1411	2.68		3.27	1.74	13.9	1942	0.82	2065	1.67	151	2.83	20.4	0.49	0.16	0.32	2.81	0.04	3.85	1475	0.14	1752	2.36	3.00	0.41	1.78	0.35	0.13	0.43	0.05	0.33	0.07	0.21	0.02	0.19	0.03	0.51	0.04	0.17	0.01	523	0.35	0.38	0.23
**BM 1983,1108.7°**							8.7	0.18	1.11	53.3	0.11	1.11	4.22	4.81	0.03	0.07	0.17	9.09	41.2	4.10	1.42	29.9	21.6	7161	8823	1.51	18888	31.3	61	2.39	27.1	0.83	0.35	8.38	4.16	0.90	202	479	0.45	293	2.05	4.12	0.43	1.75	0.39	0.08	0.38	0.05	0.33	0.06	0.19	0.02	0.20	0.03	0.71	0.07	0.40	0.21	199868	19.63	0.62	0.54
**BM 1983,1108.8°**							7.8	0.11	1.32	49.6	0.05	1.35	3.51	4.02	0.03	0.06	0.15	8.71	28.9	3.44	0.62	1.51	10.4	324	1047	1.75	16695	25.1	50	3.71	23.1	0.94	0.28	3.59	2.55	0.13	19.0	230	0.56	362	2.47	4.43	0.52	2.20	0.45	0.12	0.48	0.07	0.46	0.10	0.25	0.04	0.30	0.04	0.64	0.17	1.65	13.89	273620	34.98	0.80	0.42
